# Supplementary material for: Federated knowledge retrieval elevates large language model performance on biomedical benchmarks
Source: Gigascience. 2026 Jan 19;15:giag007. doi: 10.1093/gigascience/giag007 (PMC12888809; doi:10.1093/gigascience/giag007)
Supplement: giag007_Supplemental_File [file giag007_supplemental_file.pdf]

Supplementary Figures & Tables

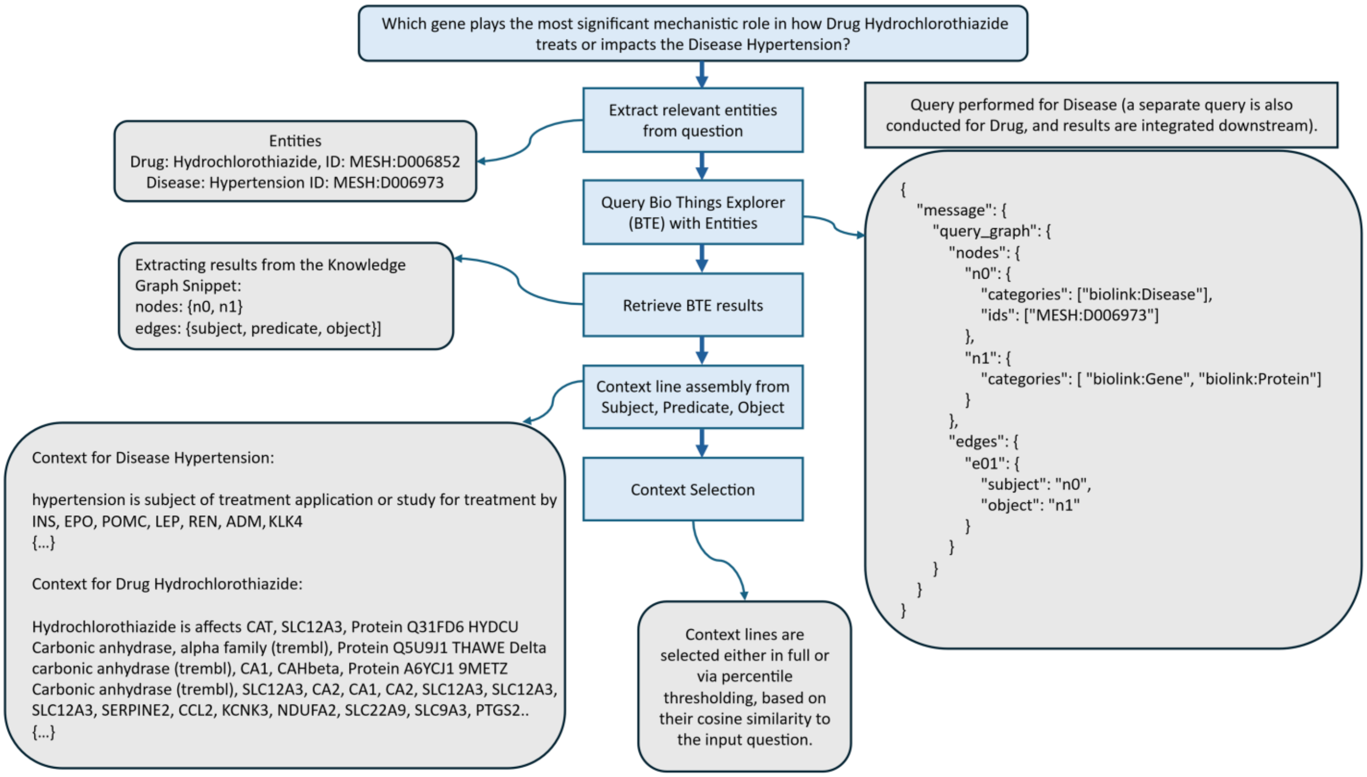

Figure S1: Detailed pipeline for BTE-RAG

Supplementary Figure S1 depicts the end-to-end workflow through which the BTE-RAG retrieval module converts a biomedical question into the evidence snippets ultimately supplied to the language-model reasoner. Beginning with an example query, “Which gene plays the most significant mechanistic role in how the drug *hydrochlorothiazide* treats or impacts the disease *hypertension*?”, the system first performs named-entity recognition, normalizing the detected concepts to controlled identifiers (Drug: MESH:D006852; Disease: MESH:D006973). Each entity is then submitted to BioThings Explorer (BTE) as part of a query graph that requests mechanistically relevant genes and proteins; independent queries are executed for the drug and for the disease. BTE returns knowledge-graph sub-graphs whose nodes and edges represent subject-predicate-object triples grounded in the biomedical literature. These triples are linearized into plain-text sentences, yielding two preliminary corpora (one for the disease, one for the drug) that list, for example, genes such as *INS*, *REN*, *SLC12A3* and *PTGS2* with their associated predicates. Finally, the complete set of sentences or a percentile-filtered subset is ranked by cosine similarity to the original question, and the highest-scoring lines are selected as the “retrieved context” passed forward for answer generation.

Benchmark	No. of Questions	Drug Precision/Recall/ F1 score	Disease Precision/Recall/F1 score	Biological-Process Precision/Recall/F1 score	Joint Accuracy
**Gene-Centric: Drug-Gene-Disease**	**798**	**0.993/0.995/0.994**	**0.985/0.990/0.988**	**~**	**0.985**
**Metabolite-Centric: Drug-Metabolite-Disease**	**201**	**0.995/1.000/0.998**	**0.975/0.985/0.980**	**~**	**0.985**
**Drug-Bio.Process-Centric: Drug-Biological Process-Disease**	**842**	**~**	**0.911/0.945/0.928**	**0.906/0.944/0.925**	**0.892**

Table S1: Zero-Shot Entity Recognition Performance

Performance of the zero-shot entity recognition module evaluated across all benchmark questions. Precision, recall, and F1 are averaged across all entity types required per benchmark. *Joint accuracy* reflects the proportion of questions for which all required entities were correctly extracted. These results demonstrate that automated entity extraction is feasible for end-to-end BTE-RAG deployment.

Model	Comparison	Method1_Acc (%)	Method2_Acc (%)	Difference	Newcombe CI	McNemar P-value
**gpt-4o-mini**	BTE-RAG vs LLM-only	77 [67.85%, 84.16%]	33 [24.56%, 42.69%]	44	[30.67, 55.06]	2.297590e-10
**gpt-4o-mini**	BTE-RAG vs GeneGPT-Full	77	29 [21.01%, 38.54%]	48	[34.78, 58.72]	2.995260e-11
**gpt-4o-mini**	BTE-RAG vs GeneGPT-Slim	77	32 [23.67%, 41.66%]	45	[31.69, 55.98]	3.263497e-10
**gpt-4o**	BTE-RAG vs LLM-only	80 [71.12%, 86.66%]	56 [46.23%, 65.33%]	24	[11.12, 35.83]	2.678522e-05
**gpt-4o**	BTE-RAG vs GeneGPT-Full	80	59 [49.20%, 68.13%]	21	[8.26, 32.85]	2.040840e-04
**gpt-4o**	BTE-RAG vs GeneGPT-Slim	80	64 [54.24%, 72.73%]	16	[3.55, 27.82]	3.263717e-03

Table S2: Statistical comparison of BTE-RAG with baseline methods on the GeneTuring gene–disease association benchmark.

Pairwise comparisons between BTE-RAG and baseline approaches (LLM-only prompting, GeneGPT-Full, and GeneGPT-Slim) evaluated using gpt-4o-mini and gpt-4o base models. For each comparison, the table reports method accuracies, absolute accuracy differences, 95% confidence intervals for the difference computed using the Newcombe hybrid score method, and McNemar's test p-values for paired proportions. All comparisons demonstrate statistically significant improvements for BTE-RAG relative to baseline methods.

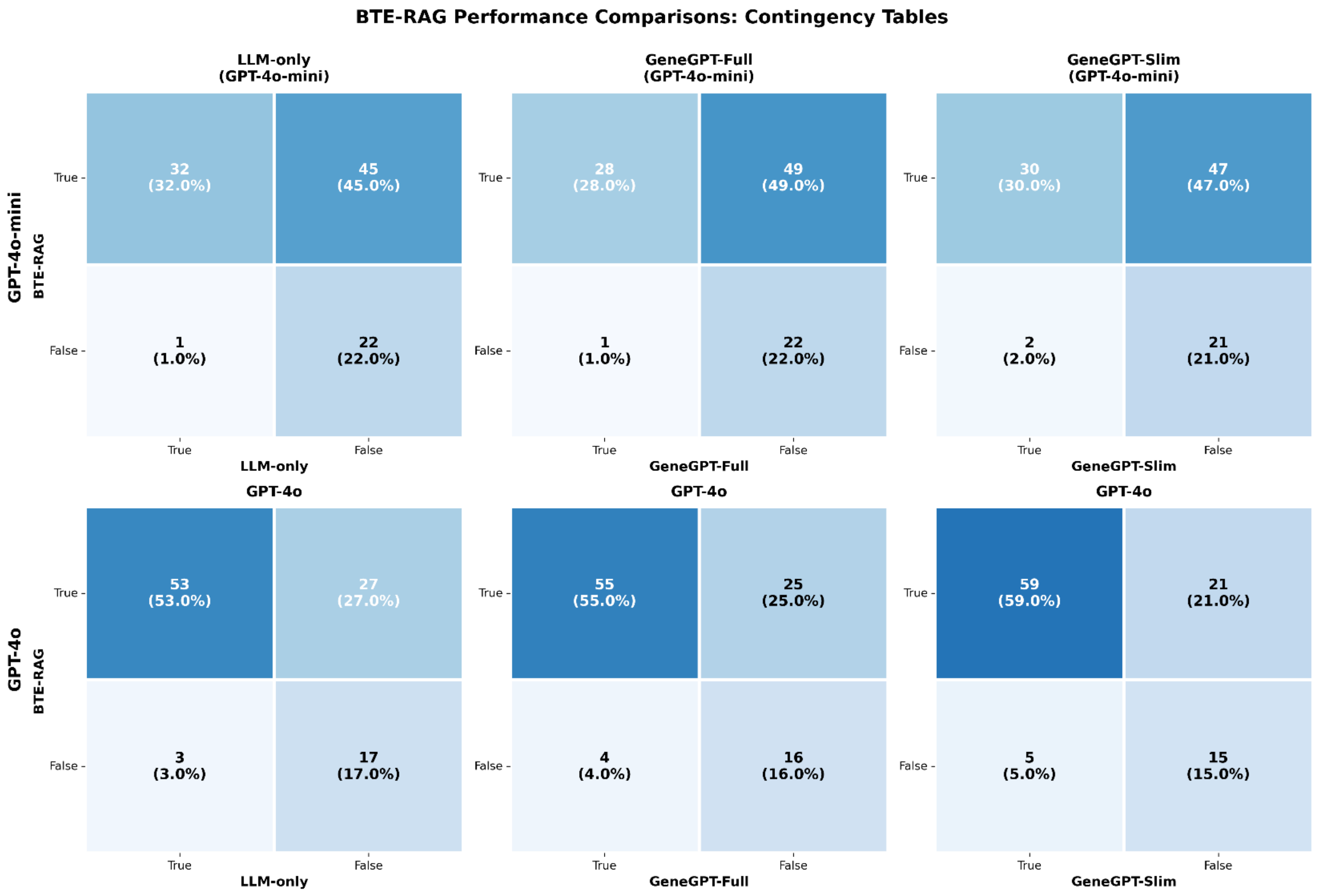

Figure S2: Contingency table analysis of BTE-RAG performance gains on the GeneTuring benchmark.

Contingency tables summarizing per-question agreement between **BTE-RAG** and baseline methods (LLM-only, GeneGPT-Full, and GeneGPT-Slim) for **gpt-4o-mini** (top row) and **gpt-4o** (bottom row). Each panel shows the number and proportion of questions where both methods answered correctly, both answered incorrectly, or where BTE-RAG corrected errors made by the baseline (upper-right cells) versus cases where baseline methods corrected errors made by BTE-RAG (lower-left cells). Across all comparisons, BTE-RAG exhibits strongly asymmetric error correction patterns, explaining the significant McNemar test results reported in Table S2.

Model	Comparison	Method1_Acc (%)	Method2_Acc (%)	Difference	Newcombe CI	McNemar P-value
**gpt-4o-mini**	BTE-RAG vs LLM-only	75.81 [72.72%, 78.66%]	51 [47.54%, 54.46%]	24.81	[20.18, 29.29]	9.472237e-31
**gpt-4o-mini**	BTE-RAG vs GeneGPT-Full	75.81	37.22 [33.93%, 40.63%]	38.59	[34.00, 42.94]	1.434960e-58
**gpt-4o-mini**	BTE-RAG vs GeneGPT-Slim	75.81	45.24 [41.82%, 48.71%]	30.57	[25.93, 35.03]	8.851814e-42
**gpt-4o**	BTE-RAG vs LLM-only	78.57 [75.59%, 81.28%]	69.80 [66.53%, 72.88%]	8.77	[4.48, 13.02]	1.018087e-07
**gpt-4o**	BTE-RAG vs GeneGPT-Full	78.57	42.73 [39.34%, 46.19%]	35.84	[31.27, 40.18]	3.364883e-54
**gpt-4o**	BTE-RAG vs GeneGPT-Slim	78.57	43.98 [40.58%, 47.45%]	34.59	[30.02, 38.94]	5.193384e-53

Table S3: Statistical comparison of BTE-RAG with baseline methods on the Mechanistic Gene benchmark.

Pairwise comparisons between BTE-RAG and baseline approaches (LLM-only prompting, GeneGPT-Full, and GeneGPT-Slim) evaluated using gpt-4o-mini and gpt-4o base models. For each comparison, the table reports method accuracies, absolute accuracy differences, 95% confidence intervals for the difference computed using the Newcombe hybrid score method, and McNemar's test p-values for paired proportions. All comparisons demonstrate statistically significant improvements for BTE-RAG relative to baseline methods.

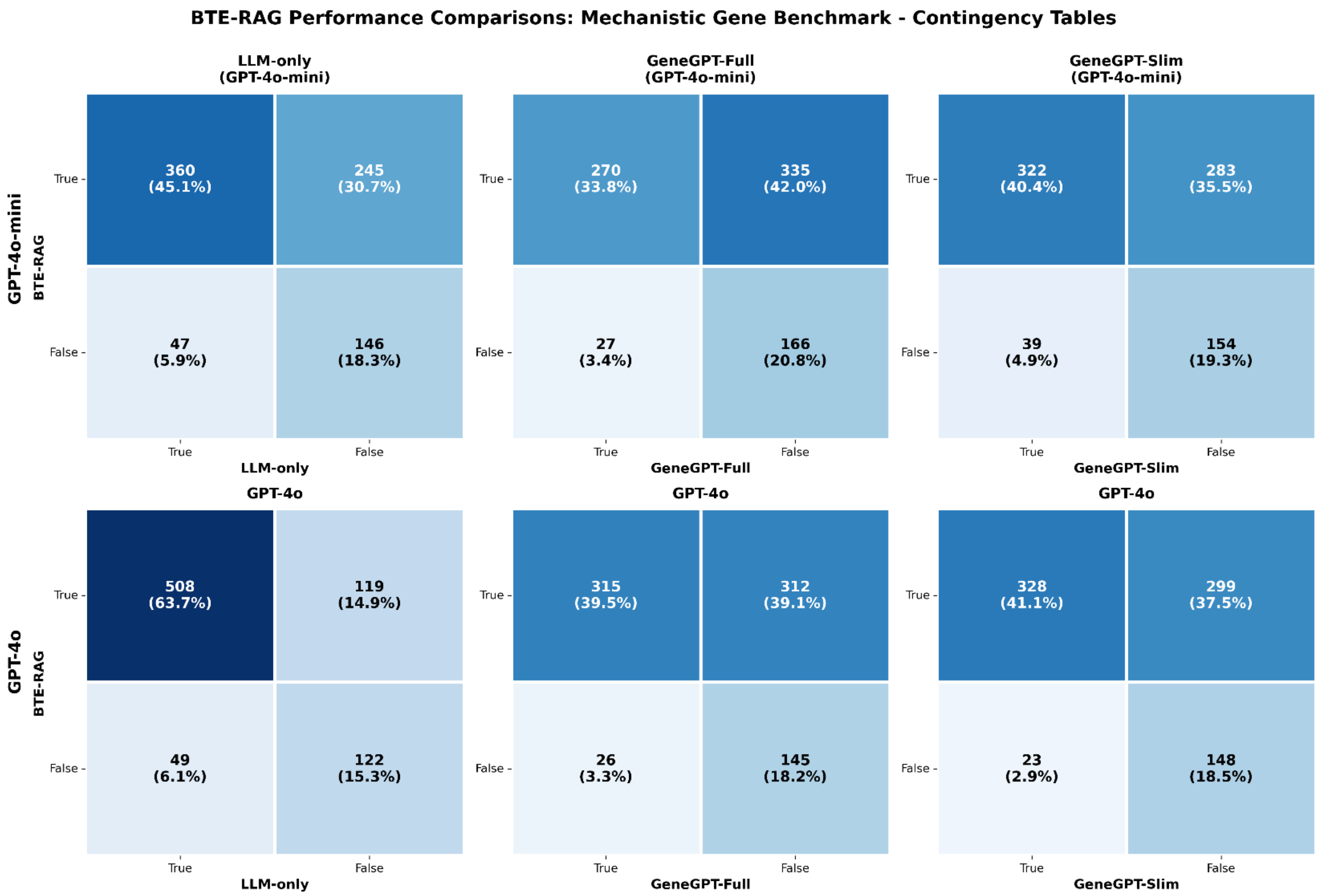

Figure S3: Contingency table analysis of BTE-RAG performance gains on the Mechanistic Gene benchmark.

Contingency tables summarizing per-question agreement between **BTE-RAG** and baseline methods (LLM-only, GeneGPT-Full, and GeneGPT-Slim) for **gpt-4o-mini** (top row) and **gpt-4o** (bottom row) for the mechanistic gene benchmark.

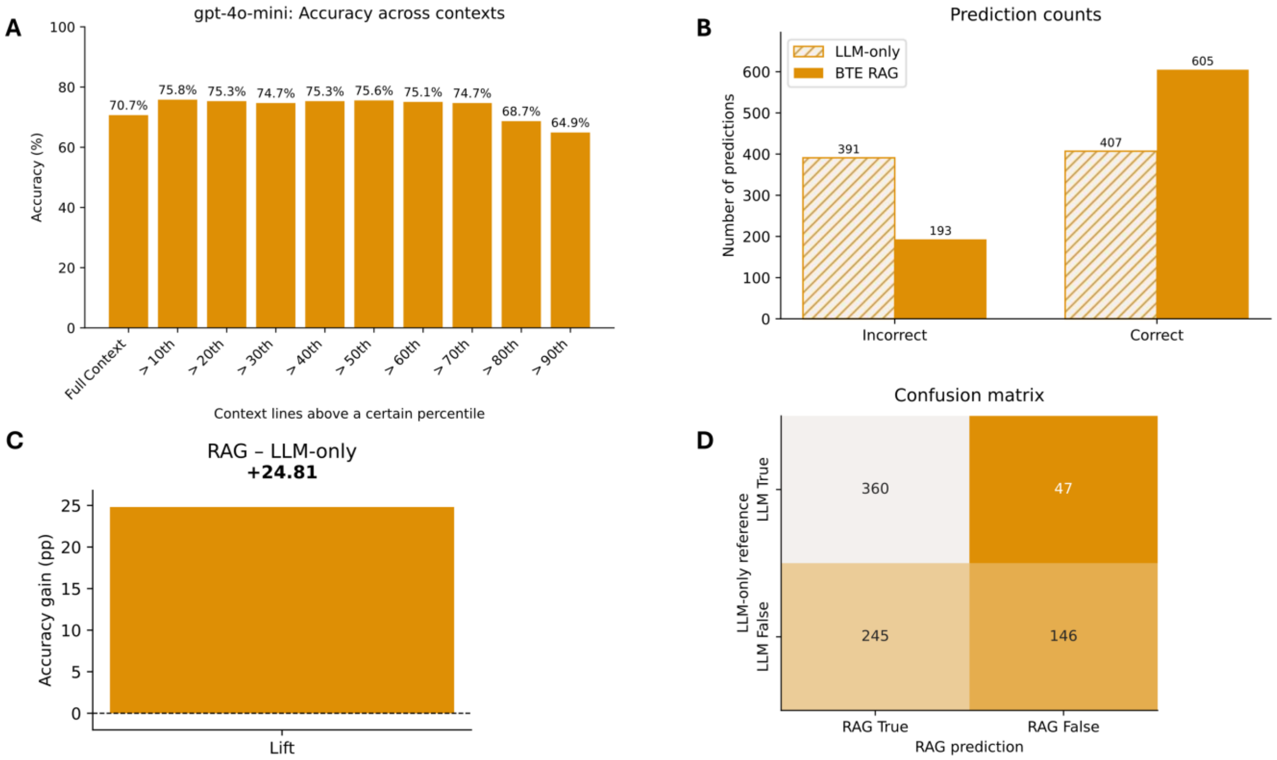

Figure S4: Performance of BTE-RAG versus an LLM-only baseline on the gene-centric benchmark using gpt-4o-mini.

(A) Overall accuracy as a function of how much of the retrieved context is retained. Bars show accuracy when only context lines above a given cosine-similarity percentile are supplied to the model (10 th–90 th) as well as when the full context is used.
(B) Breakdown of prediction counts for the 798 benchmark questions. The hatched bars represent the LLM-only condition; solid bars represent BTE-RAG.
(C) BTE-RAG outperforms the LLM-only run by +24.8 percentage points, confirming that targeted knowledge-graph snippets materially improve answer quality.
(D) Confusion matrix comparing the two methods. The upper-left cell (360 cases) denotes questions both methods answer correctly; the lower-left cell (245) highlights errors that BTE-RAG fixes; the upper-right cell (47) shows instances where retrieval introduces an error; and the lower-right cell (146) comprises questions neither approach resolves.

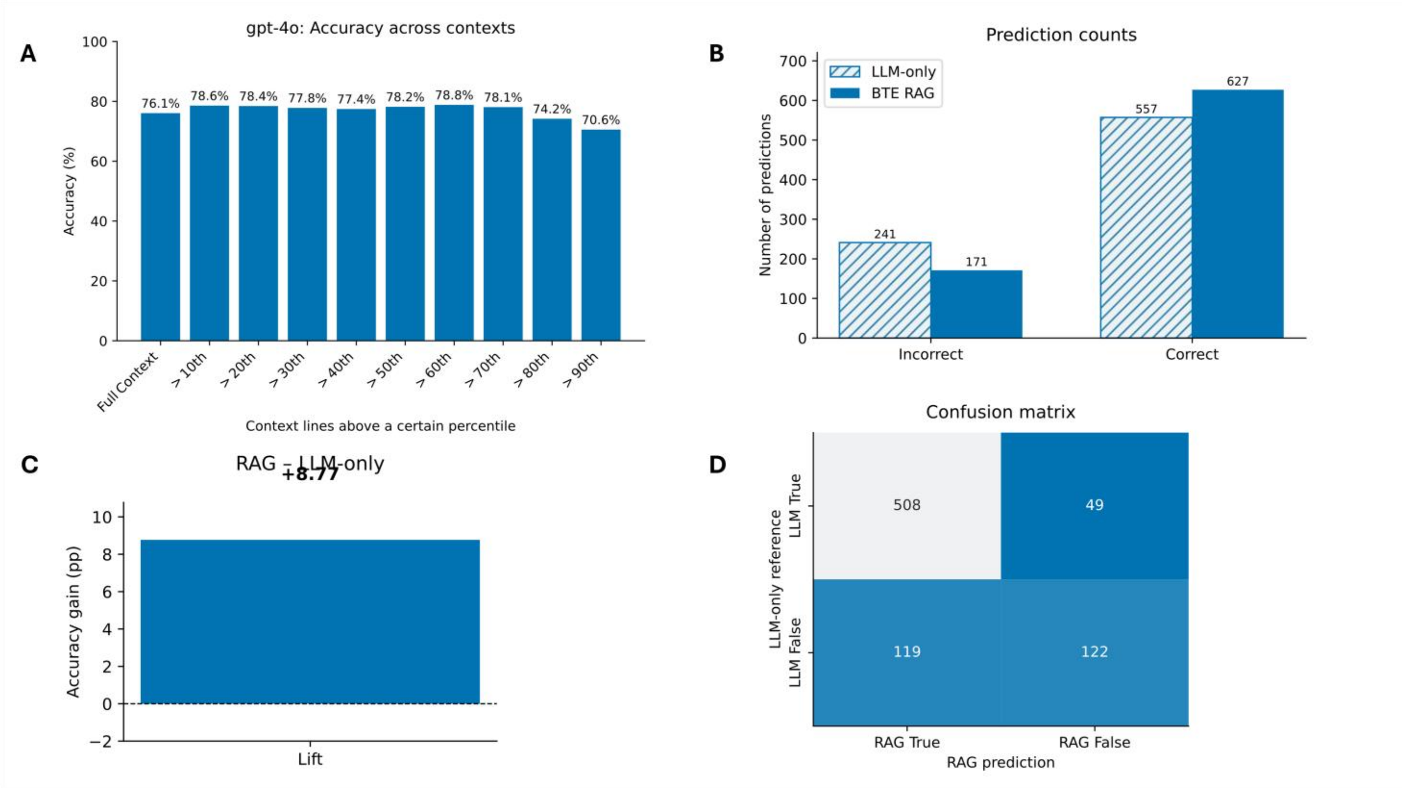

Figure S5: Performance of BTE-RAG versus an LLM-only baseline on the gene-centric benchmark using gpt-4o.

(A) Overall accuracy as a function of how much of the retrieved context is retained. Bars show accuracy when only context lines above a given cosine-similarity percentile are supplied to the model (10 th–90 th) as well as when the full context is used.
(B) Breakdown of prediction counts for the 798 benchmark questions. The hatched bars represent the LLM-only condition; solid bars represent BTE-RAG.
(C) BTE-RAG outperforms the LLM-only run by +8.8 percentage points, confirming that targeted knowledge-graph snippets materially improve answer quality.
(D) Confusion matrix comparing the two methods. The upper-left cell (508 cases) denotes questions both methods answer correctly; the lower-left cell (119) highlights errors that BTE-RAG fixes; the upper-right cell (49) shows instances where retrieval introduces an error; and the lower-right cell (122) comprises questions neither approach resolves.

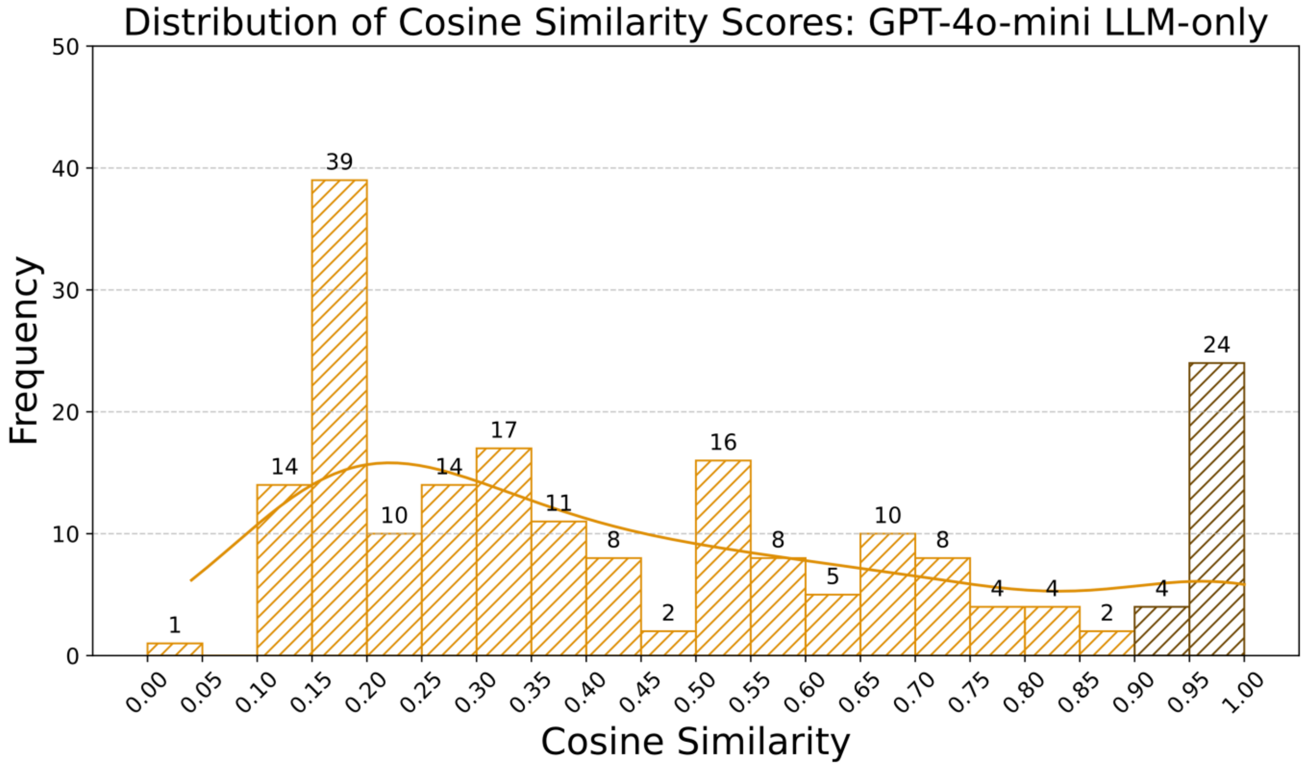

Figure S6: Cosine-similarity profile for the metabolite-centric benchmark using GPT-4o-mini in LLM-only mode.

Histogram shows the frequency of cosine-similarity scores (bin width = 0.05) between model answers and ground truth answers across 201 metabolite-related queries when using gpt-4o-mini. A smoothed kernel-density curve traces the overall score profile.

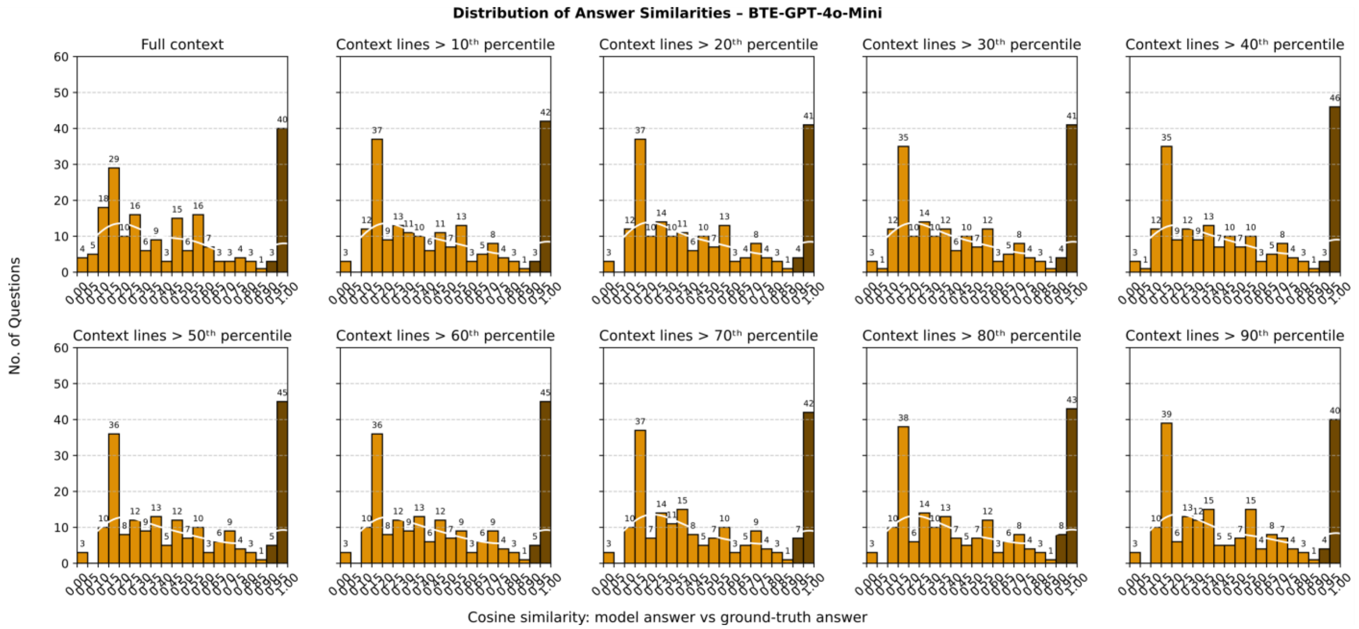

Figure S7: Distribution of answer similarities for the metabolite-centric benchmark using GPT-4o-mini in BTE-RAG mode.

Each panel shows the cosine similarity between model predictions and ground-truth answers when either the full retrieved context is used (top left) or when context lines are filtered above increasing cosine similarity percentiles (10th to 90th).

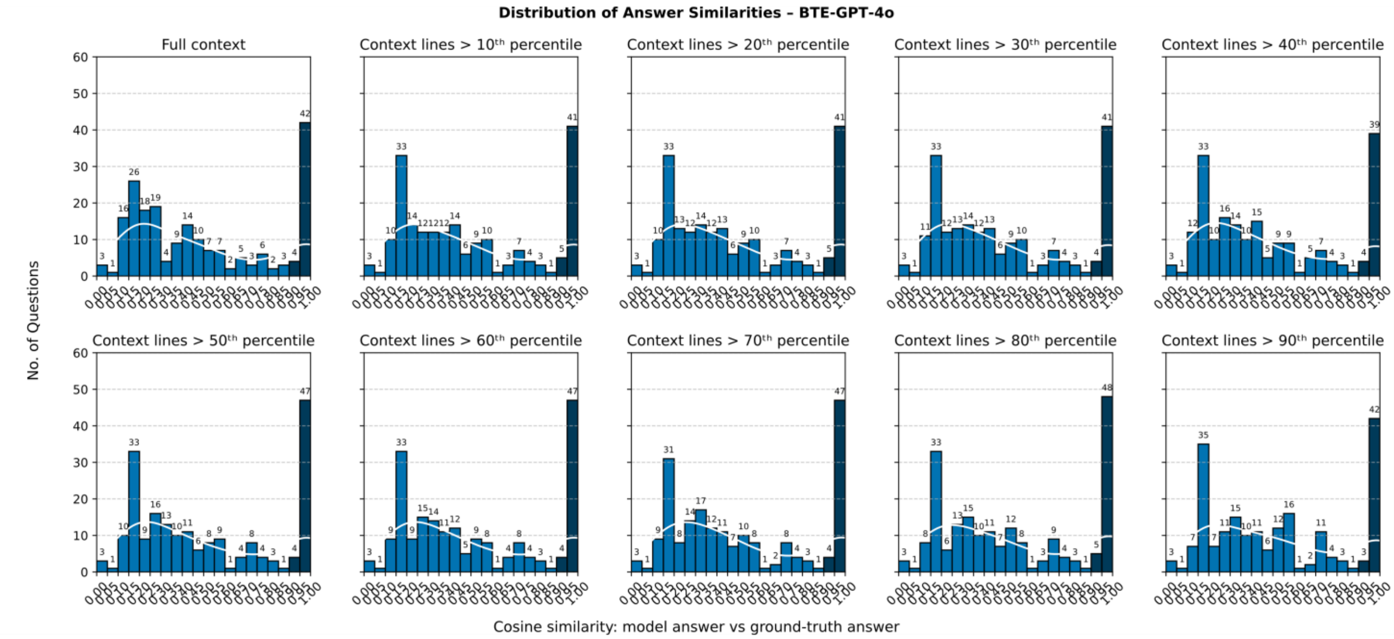

Figure S8: Distribution of answer similarities for the metabolite-centric benchmark using GPT-4o in BTE-RAG mode.

Each panel shows the cosine similarity between model predictions and ground-truth answers when either the full retrieved context is used (top left) or when context lines are filtered above increasing cosine similarity percentiles (10th to 90th).

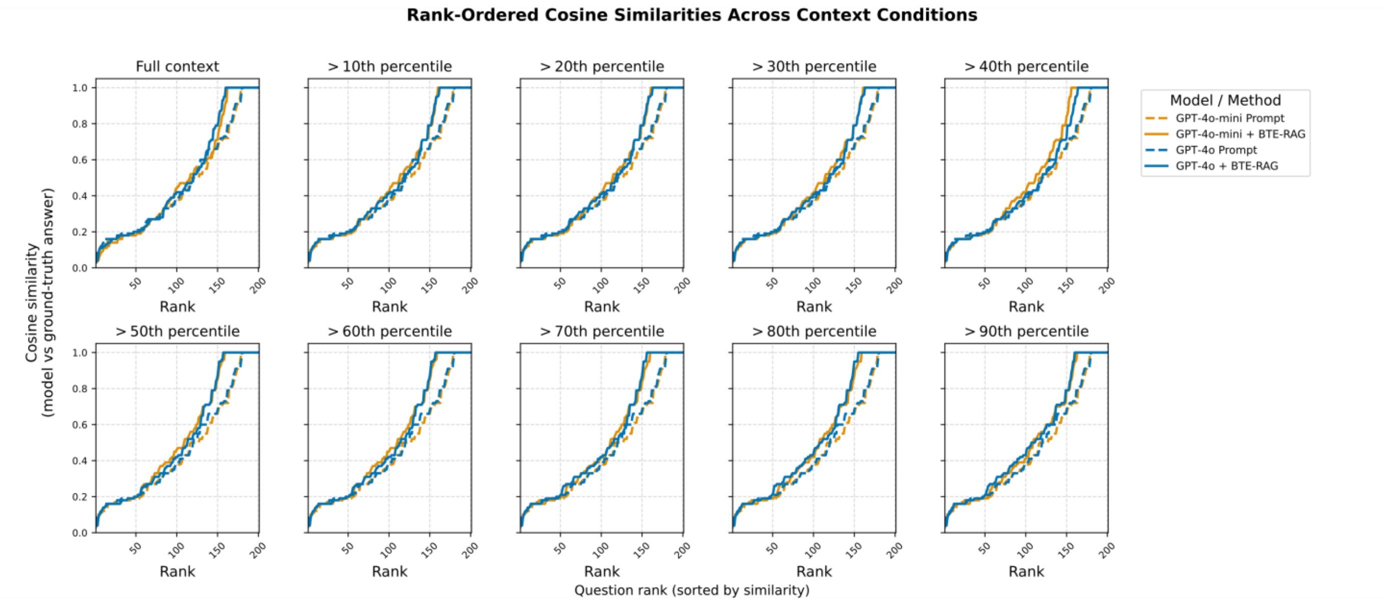

Figure S9: Rank-ordered cosine similarities between model predictions and ground-truth answers on the metabolite-centric benchmark, across context filtering thresholds.

Each panel displays results from four model–method combinations (GPT-4o-mini-Prompt (LLM-only), GPT-4o-mini + BTE-RAG, GPT-4o Prompt, GPT-4o + BTE-RAG) under either full context or filtered context lines exceeding the indicated cosine similarity percentile (10th to 90th). Question predictions are sorted by similarity, revealing how context filtering and model selection affect semantic alignment with ground truth.

Model	Similarity Threshold	LLM % Above Thresh old	RAG % Above Thresh old	Δ Percentage Points (RAG – LLM)	Cliff's Delta (Effect Size)	95% CI for Cliff's δ
GPT-4o-mini	0.85	15.4	26.4	10.9	0.109	[0.05, 0.16]
GPT-4o-mini	0.90	13.9	25.4	11.4	0.114	[0.06, 0.17]
GPT-4o-mini	0.95	11.9	23.4	11.4	0.114	[0.06, 0.17]
GPT-4o	0.85	16.4	27.4	10.9	0.109	[0.05, 0.16]
GPT-4o	0.90	14.9	26.4	11.4	0.114	[0.06, 0.17]
GPT-4o	0.95	11.4	25.9	14.4	0.144	[0.09, 0.19]

Table S4: Sensitivity analysis of similarity thresholds for high-fidelity drug–metabolite predictions.

Proportion of predictions exceeding cosine-similarity thresholds of 0.85, 0.90, and 0.95 for LLM-only and BTE-RAG conditions, evaluated using GPT-4o-mini and GPT-4o. For each threshold, the table reports the percentage of high-fidelity predictions, the absolute increase attributable to retrieval augmentation (Δ percentage points), and the corresponding Cliff's delta effect size with 95% confidence intervals.

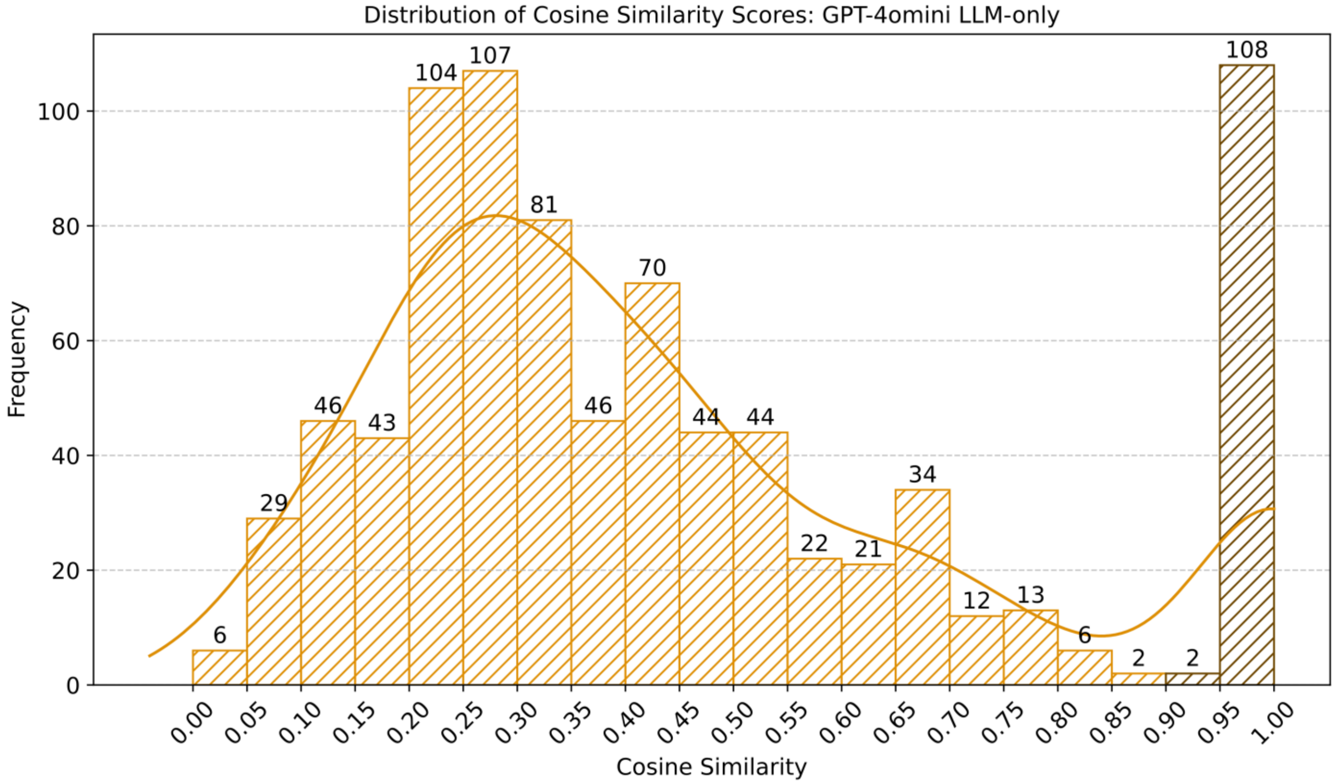

Figure S10: Cosine-similarity profile for the drug-centric benchmark using GPT-4o-mini in LLM-only mode.

Histogram shows the frequency of cosine-similarity scores (bin width = 0.05) between model answers and ground truth answers across 842 drug-biological process queries when using gpt-4o-mini. A smoothed kernel-density curve traces the overall score profile.

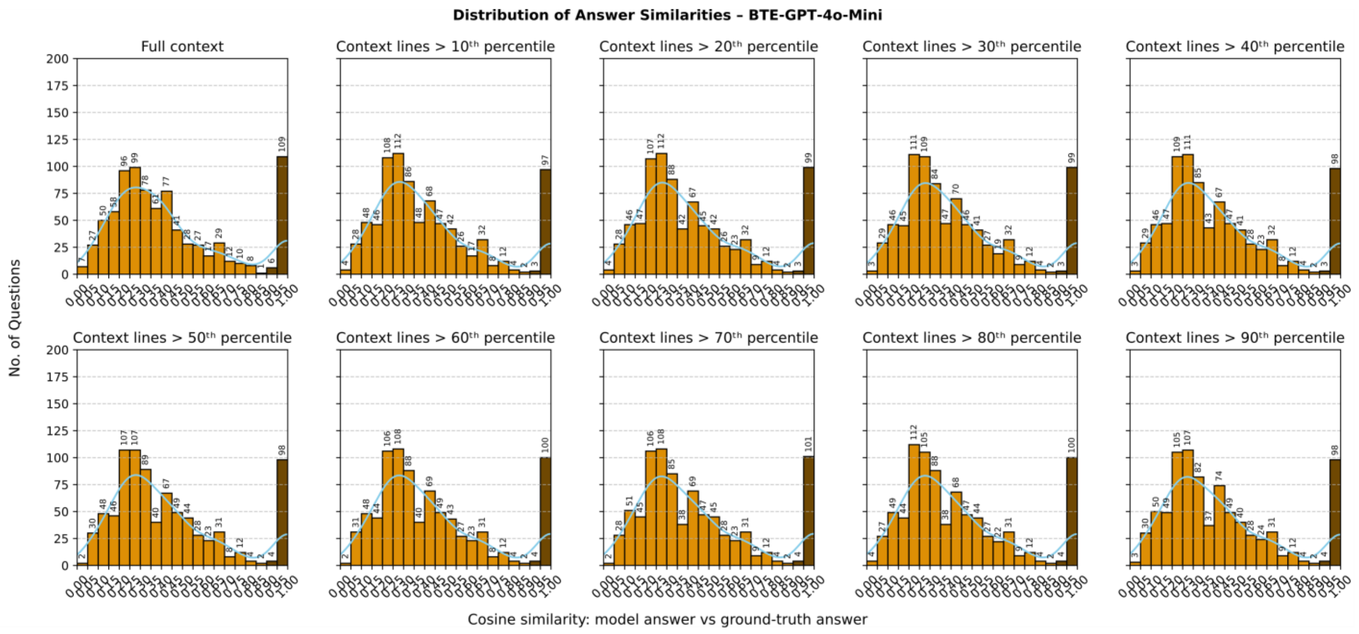

Figure S11: Distribution of answer similarities for the drug-centric benchmark using GPT-4o-mini in BTE-RAG mode.

Each panel shows the cosine similarity between model predictions and ground-truth answers when either the full retrieved context is used (top left) or when context lines are filtered above increasing cosine similarity percentiles (10th to 90th).

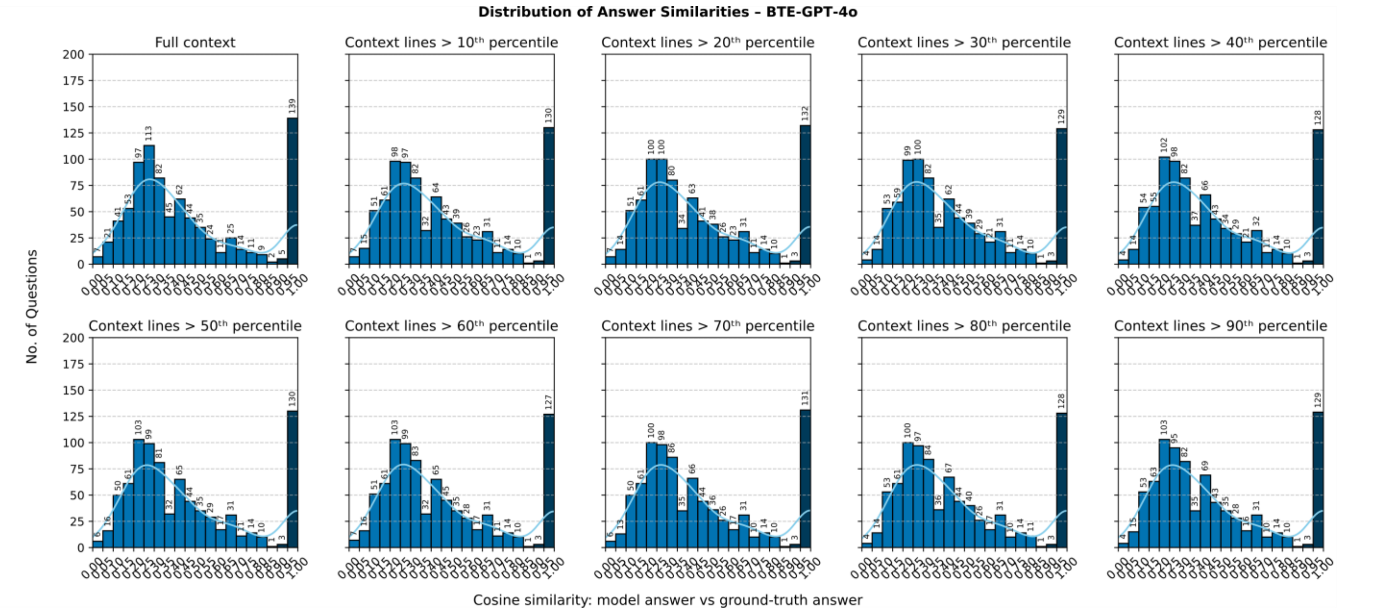

Figure S12: Distribution of answer similarities for the drug-centric benchmark using GPT-4o in BTE-RAG mode.

Each panel shows the cosine similarity between model predictions and ground-truth answers when either the full retrieved context is used (top left) or when context lines are filtered above increasing cosine similarity percentiles (10th to 90th).

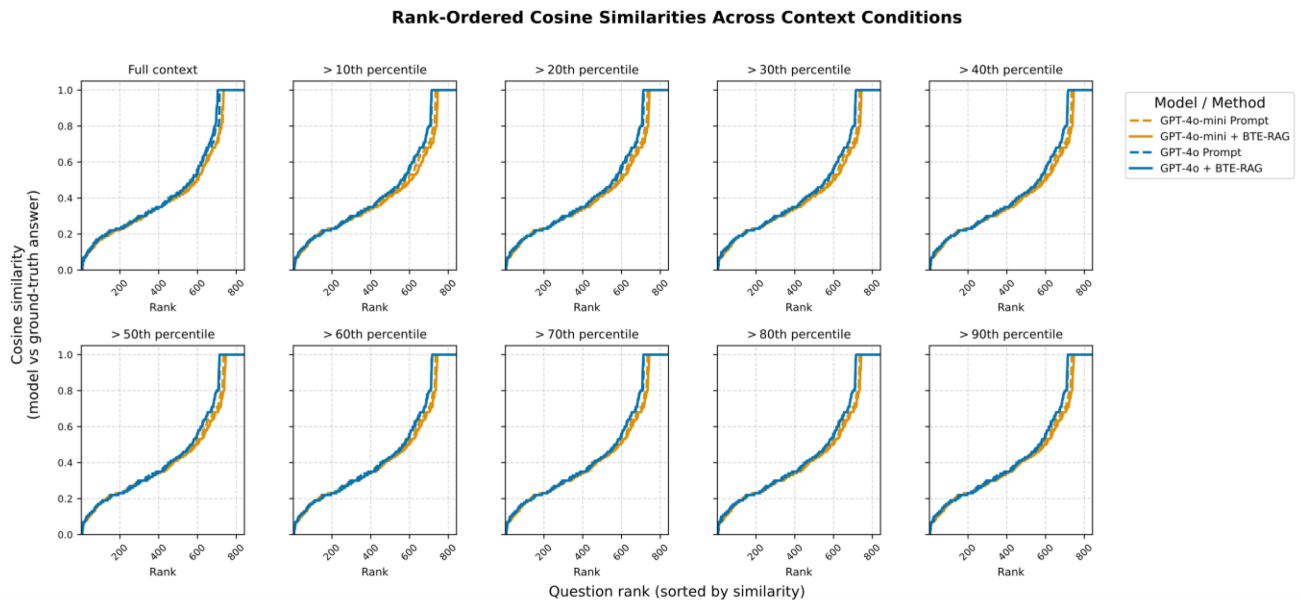

Figure S13: Rank-ordered cosine similarities between model predictions and ground-truth answers on the drug-centric benchmark, across context filtering thresholds.

Each panel displays results from four model–method combinations (GPT-4o-mini Prompt (LLM-only), GPT-4o-mini + BTE-RAG, GPT-4o Prompt, GPT-4o + BTE-RAG) under either full context or filtered context lines exceeding the indicated cosine similarity percentile (10th to 90th). Question predictions are sorted by similarity, revealing how context filtering and model selection affect semantic alignment with ground truth.

Model	Similarity Threshold	LLM % Above Threshold	RAG % Above Threshold	Δ Percentage Points (RAG – LLM)	Cliff's Delta (Effect Size)	95% CI for Cliff's δ
GPT-4o-mini	0.85	13.3	13.8	0.5	0.005	[-0.02, 0.03]
GPT-4o-mini	0.90	13.1	13.7	0.6	0.006	[-0.02, 0.03]
GPT-4o-mini	0.95	12.9	12.9	0.0	0.000	[-0.02, 0.02]
GPT-4o	0.85	15.6	17.3	1.8	0.018	[-0.00, 0.04]
GPT-4o	0.90	15.3	17.1	1.8	0.018	[-0.00, 0.04]
GPT-4o	0.95	15.3	16.5	1.2	0.012	[-0.01, 0.03]

Table S5: Sensitivity analysis of similarity thresholds for high-fidelity drug–biological process-disease predictions.

Proportion of predictions exceeding cosine-similarity thresholds of 0.85, 0.90, and 0.95 for LLM-only and BTE-RAG conditions, evaluated using GPT-4o-mini and GPT-4o. For each threshold, the table reports the percentage of high-fidelity predictions, the absolute increase attributable to retrieval augmentation (Δ percentage points), and the corresponding Cliff's delta effect size with 95% confidence intervals.

Metric	Gene Task	Metabolite Task	Drug-BP Task
**Degradation Cases**			
Total in dataset	47 (5.9%)	6	40 (4.7%)
Cases analyzed	20 (42.6%)	6 (100%)	20 (50%)
**Error Categories (%)**			
Context Dilution	18 (90.0%)	2 (33.3%)	18 (90.0%)
Coverage Gap	1 (5.0%)	4 (66.7%)	1 (5.0%)
Filtering Error	1 (5.0%)	—	—
Derivative Mismatch			1 (5.0%)
**Performance Scores**			
LLM baseline (mean)	—	0.96	1.00
BTE-RAG (mean)	—	0.36	0.39
**Similarity Drop**			
Mean ± SD	—	0.59 ± 0.16	0.60 ± 0.17
Median [Range]	—	0.56 [0.44–0.82]	0.61 [0.20–0.90]

Table S6: Analysis of Degradation Cases Across Gene, Metabolite, and Drug–Biological Process Benchmarks

This table summarizes all instances in which BTE-RAG performance decreased relative to the LLM-only baseline ("degradation cases") across the Gene, Metabolite, and Drug–Biological Process mechanistic reasoning tasks for the GPT-4o-mini model. For each task, we report the total number of degradation cases, the subset selected for manual review, and the distribution of underlying error modes: context dilution *(excess or weakly relevant retrieved evidence obscuring the gold entity)*, coverage gaps *(missing or incomplete mechanistic edges in the federated knowledge graph)*, filtering errors *(over-pruning of relevant evidence during context selection)*, and formulation/derivative mismatches *(e.g., chloramphenicol palmitate instead of chloramphenicol)*. Performance metrics include baseline LLM similarity, BTE-RAG similarity, and the corresponding similarity drops (mean ± SD and median range).

Table S7: System Prompts

Gene-centric benchmark

LLM-only	BTE-RAG
You are an expert biomedical researcher. Please provide your answer (only gene name) in the following JSON format for the Question asked:<br>{<br>"answer": <correct answer><br>}	You are an advanced biomedical researcher. Use your most recent knowledge in addition to the Context provided when needed to answer accurately.<br>*Answer Format*: Provide your answer (only the gene name) in the following JSON format:<br>{<br>"answer": <correct answer><br>}

Metabolite-centric benchmark

LLM-only	BTE-RAG
You are an expert biomedical researcher. Please provide your answer (only the biochemical entity name) in the following JSON format for the Question asked:<br>{{<br>"answer": <correct answer><br>}}	You are an advanced biochemistry researcher. Use your most recent knowledge in addition to the Context provided when needed to answer accurately.<br>*Answer Format*: Provide your answer (only the biochemical entity name) in the following JSON format:<br>{<br>"answer": <correct answer><br>}

Drug-centric benchmark

LLM-only	BTE-RAG
You are an expert biomedical researcher. Please provide your answer (only Drug names) in the following JSON format for the Question asked:<br>{{<br>"answer": <correct answer><br>}}	You are an **advanced biomedical research AI**, specializing in answering biological and biomedical questions with accuracy. Use the provided CONTEXT along with your most recent knowledge to answer the question.<br>*Answer Format*: Provide your answer (only Drug name) in the following JSON format:<br>{<br>"answer": <correct answer><br>}
