# Supplementary material for: Federated knowledge retrieval elevates large language model performance on biomedical benchmarks
Source: Gigascience. 2026 Jan 19;15:giag007. doi: 10.1093/gigascience/giag007 (PMC12888809; doi:10.1093/gigascience/giag007)
Supplement: giag007_GIGA-D-25-00307_Revision_1 [file giag007_giga-d-25-00307_revision_1.pdf]

GigaScience

Federated Knowledge Retrieval Elevates Large Language Model Performance on Biomedical Benchmarks

--Manuscript Draft--

**Manuscript Number:**	GIGA-D-25-00307R1
**Full Title:**	Federated Knowledge Retrieval Elevates Large Language Model Performance on Biomedical Benchmarks
**Article Type:**	Research
**Funding Information:**	National Institute on Aging (R01AG066750) — Dr. Andrew I. Su<br>National Center for Advancing Translational Sciences (1OT2TR003427) — Dr. Andrew I. Su<br>National Center for Advancing Translational Sciences (1OT2TR005710) — Dr. Andrew I. Su<br>National Center for Advancing Translational Sciences (UM1TR004407) — Dr. Andrew I. Su
**Abstract:**	Background<br>Large language models (LLMs) have significantly advanced natural language processing in biomedical research, however, their reliance on implicit, statistical representations often results in factual inaccuracies or hallucinations, posing significant concerns in high-stakes biomedical contexts.<br>Results<br>To overcome these limitations, we developed BTE-RAG, a retrieval-augmented generation framework that integrates the reasoning capabilities of advanced language models with explicit mechanistic evidence sourced from BioThings Explorer, an API federation of more than sixty authoritative biomedical knowledge sources. We systematically evaluated BTE-RAG in comparison to traditional LLM-only methods across three benchmark datasets that we created from DrugMechDB. These datasets specifically targeted gene-centric mechanisms (798 questions), metabolite effects (201 questions), and drug–biological process relationships (842 questions). On the gene-centric task, BTE-RAG increased accuracy from 51% to 75.8% for GPT-4o mini and from 69.8% to 78.6% for GPT-4o. In metabolite-focused questions, the proportion of responses with cosine similarity scores of at least 0.90 rose by 82% for GPT-4o mini and 77% for GPT-4o. While overall accuracy was consistent in the drug–biological process benchmark, the retrieval method enhanced response concordance, producing a greater than 10% increase in high-agreement answers (from 129 to 144) using GPT-4o. We additionally evaluated BTE-RAG alongside GeneGPT-based models on the GeneTuring gene–disease association benchmark and on our mechanistic gene benchmark, demonstrating that the BTE-RAG layer consistently improves accuracy relative to alternative approaches.<br>Conclusion<br>Federated knowledge retrieval provides transparent improvements in accuracy for large language models, establishing BTE-RAG as a valuable and practical tool for mechanistic exploration and translational biomedical research.
**Corresponding Author:**	Andrew Su<br>The Scripps Research Institute<br>La Jolla, CA UNITED STATES
**Corresponding Author Secondary Information:**	
**Corresponding Author's Institution:**	The Scripps Research Institute
**Corresponding Author's Secondary Institution:**	
**First Author:**	Janet Joy

Powered by Editorial Manager® and ProduXion Manager® from Aries Systems Corporation

**First Author Secondary Information:**	
**Order of Authors:**	Janet Joy
	Andrew I. Su, Ph.D.
**Order of Authors Secondary Information:**	
**Response to Reviewers:**	The response is also included in the joy_reviewer_response.pdf<br><br>-----------------<br><br>Thank you for the thoughtful comments and suggestions. We have addressed all points and incorporated the corresponding revisions into the manuscript, with changes highlighted in blue in the relevant sections.<br>Reviewer's Comments<br><br>Reviewer #1<br>Thank you for the opportunity to review "Federated Knowledge Retrieval Elevates Large Language Model Performance on Biomedical Benchmarks." The paper tackles a timely and important problem: grounding large language models in mechanistic evidence to reduce unsupported claims. It does so with a thoughtful design that layers BTE-RAG over a federation of approximately 60 biomedical APIs and evaluates three complementary DrugMechDB-derived benchmarks (gene, metabolite, drug to process). The manuscript is clearly written, the technical contribution is meaningful, and the experimental results are promising.<br><br>Recommendation: Major revision.<br><br>Below are concrete, actionable changes that would bring the work in line with GigaScience's standards for FAIR availability, licensing, documentation, testing, and reproducibility. Many are straightforward, but together they matter for long-term reuse and auditability.<br><br>1) Statistical rigor: paired inference, uncertainty, variance<br>The manuscript reports compelling descriptive gains. Because each benchmark item is answered under both conditions (LLM-only and BTE-RAG), the study is a paired design. In paired settings, descriptive plots and point estimates are not sufficient to establish that improvements exceed sampling noise or threshold tuning. Please add paired statistical evidence that quantifies: (i) whether the gains are reliable, (ii) how large they are in practical terms, and (iii) how stable they are under repeated runs or under a fully deterministic pipeline.<br><br>Gene task (binary): Report McNemar's test on the existing 2×2 tables, along with 95 percent Wilson confidence intervals for each condition and a Newcombe confidence interval for the accuracy difference. Keep the flip counts in the text.<br>Metabolite and drug-to-process tasks (similarity): Report paired bootstrap confidence intervals or Wilcoxon signed-rank tests on per-item similarity differences (BTE-RAG minus baseline). Include a nonparametric effect size such as Cliff's delta with its confidence interval.<br>Threshold validation: Treat the greater-than-or-equal-to 0.90 "high-fidelity" threshold as a choice that should be validated. Show sensitivity across nearby cutoffs such as 0.85, 0.90, and 0.95, and add a small blinded expert adjudication (about 50 to 100 items) to confirm that the high-cosine band corresponds to acceptable correctness.<br>Variance or determinism: Either document end-to-end determinism (frozen retrieval caches, fixed ordering, pinned embeddings) or run at least three replicates and report mean and standard deviation.<br><br>We thank the reviewer for this helpful comment on statistical rigor and have substantially revised the analysis to incorporate paired inference, uncertainty quantification, and robustness checks, with new results added to the Results section. For the gene task, these analyses demonstrate statistically significant and practically meaningful improvements for all models (updated results in Section 3.2, Figure 3, Supplementary Table S3).<br>The metabolite task showed statistically significant improvements for both models (small effect for GPT-4o-mini; negligible for GPT-4o), whereas the drug–biological

process task showed no significant improvements and negligible effects. Threshold sensitivity analyses yielded consistent conclusions across both tasks, supporting robustness to threshold choice (Sections 3.3–3.4; Supplementary Tables S4–S5). We acknowledge that blinded expert analysis would strengthen validation of the similarity-based thresholds but extend beyond the current revision scope and is a good suggestion for future analysis. Our pipeline is fully deterministic and reproducible (temperature = 0, cached retrievals, fixed embedding models, and saved intermediate outputs), ensuring all reported results are exactly reproducible. The evaluation can be reproduced by re-running analysis on these cached outputs without re-querying APIs or re-generating text. This deterministic design ensures that our reported improvements are not subject to stochastic variation and would be reproduced in independent verification.

2) Benchmark scope and generalizability
All three evaluations are derived from DrugMechDB, which makes the study internally consistent but also couples the tasks to a single curation philosophy and evidence distribution. Please acknowledge this limitation explicitly in the Discussion and, ideally, add an external validation on at least one independent source to demonstrate generalizability. Options include CTD (drug-gene-process links), Reactome or GO (pathway and process grounding), DisGeNET (gene-disease associations), or a lightweight question answering set sourced outside DrugMechDB. Even a modest external set of about 100 to 200 items, evaluated with the same paired protocols and identifier-based scoring, would strengthen the claim. If full external validation is not feasible for this revision, please include robustness checks such as a date-based split, entity-family holdouts, and per-source ablations.

Thank you for this suggestion, we have now added an additional evaluation using the GeneTuring gene–disease association benchmark and analyzed four approaches on this dataset: LLM-only prompting, BTE-RAG, GeneGPT-Full, and GeneGPT-Slim. These results are reported in Section 3.1 and Figure 2 and demonstrate that the relative performance trends observed on DMDB generalize to an independent dataset. We introduced the DrugMechDB (DMDB)–derived benchmarks to specifically probe mechanistic and nontrivial relationships between drug–disease pairs, where LLM-only prompting is often insufficient because it primarily captures surface-level associations rather than explicit mechanistic reasoning.

3) Licensing, attribution, and persistent identifiers
The project is MIT-licensed and adapts components from BaranziniLab/KG_RAG (Apache-2.0) and SuLab/DrugMechDB (CC0-1.0). To meet license obligations and align with FAIR and the Joint Declaration of Data Citation Principles, please:
(i) keep Apache-licensed code under Apache with the upstream LICENSE and NOTICE files, noting any modifications;
(ii) include the CC0 dedication text for any DrugMechDB artifacts and note that CC0 provides no patent grant;
(iii) archive with DOIs (GigaDB preferred?) the three benchmarks, the exact evaluation caches used in the paper, and a tagged software release of the repository;
(iv) license datasets under CC0 or CC BY while keeping the code MIT;
(v) add a short Data and Software Availability table listing artifact, DOI or URL, license, and version or date.

Thank you for these recommendations on licensing compliance and best practices. We have relicensed all source code to Apache 2.0 (from MIT) to match upstream KG_RAG components, added a file to repo documenting modifications, and included CC0 1.0 dedication text for DrugMechDB benchmarks with explicit notation that CC0 provides no patent grant. After peer-review acceptance, we will archive a tagged software release and all benchmark datasets in the Software Heritage Archive to obtain persistent DOIs. We will then update the repository and manuscript with these identifiers as the version of record.

4) Error analysis and degradation cases
Please add a brief failure analysis focused on where BTE-RAG reduces accuracy relative to LLM-only. At minimum, report the total number and percent of right-to-wrong flips per task and include a small set of representative cases. For each example, show the input, expected and predicted outputs, the top retrieved evidence with identifiers

and timestamps, and a one-line diagnosis of the likely cause (for example normalization mismatch, retrieval coverage gap, ranking or filtering that hid relevant context, or long-context truncation). A short summary that groups the main causes into two or three buckets will make the results more interpretable and point to practical fixes.

We thank the reviewer for this important suggestion and have performed a detailed analysis of all degradation cases across the gene, metabolite, and drug–biological process benchmarks (for the GPT-4o-mini model). We categorized each case by underlying error mode and quantified the contributions of context dilution, evidence coverage gaps, filtering errors, and derivative mismatches. As summarized in the revised Results section (Supplementary Table S6) and detailed in our repository at https://github.com/janjoy/BTE-RAG/tree/main/error_analysis, most failures arose from context dilution (relevant mechanistic entities were retrieved but obscured by numerous less-specific entities), evidence coverage gaps (missing mechanistic edges in the federated knowledge graph), filtering errors (relevant evidence pruned during similarity-based selection), or derivative mismatch (e.g., predicting chloramphenicol palmitate instead of chloramphenicol).

5) Methodological transparency: embedding and scoring models
Please add two or three sentences in Methods explaining why S-PubMedBERT-MS-MARCO is used for filtering retrieved context while a BioBERT-based model is used for semantic similarity scoring, and what advantages each provides over plausible alternatives. A brief rationale will strengthen methodological transparency.

Thank you for this suggestion to improve methodological transparency. We have added a detailed rationale explaining our choice of S-PubMedBERT-MS-MARCO for retrieval filtering and the BioBERT-based model for semantic similarity scoring, which is now described in Section 2.1 (“Embedding and Scoring Models”). We agree that this clarification strengthens the manuscript.

6) Reproducibility workflow and archived caches
Because BTE federates live APIs, results can drift as sources update. Please archive the exact retrieval caches used in evaluation with DOIs and minimal provenance if at all possible (query identifier, subject and object identifiers, predicate, source name and version or access date, any confidence score, and a retrieval timestamp).

We thank the reviewer for this important point regarding reproducibility. Given that BTE queries federated APIs whose content may update over time, we ran all evaluations from a frozen set of retrieval caches to ensure reproducibility. These caches are publicly available in our GitHub repository at https://github.com/janjoy/BTE-RAG/tree/main/data/analysis_results. Each cache file contains: (i) the complete API context returned by BioThings Explorer, (ii) the subset of context passed to the LLM, and (iii) all analyzed outputs including similarity scores and accuracy measurements. Metadata includes query identifiers, subject and object identifiers (e.g., drug MeSH IDs, disease IDs, gene symbols), predicates (relationship types) etc. We also provide all cached LLM responses from both LLM-only and BTE-RAG conditions (temperature=0 for deterministic generation). This archiving ensures reproducibility of our reported results, even if upstream APIs change. We have added a Data Availability statement in the manuscript directing readers to these archived resources.

In summary, this is a promising and well-motivated study that could make a useful contribution once the statistical evidence, FAIR availability, and reproducibility pieces are tightened as outlined above. I recommend Major Revision and am happy to re-review a revised version.

Reviewer #2
This paper introduces BTE-RAG, a system that combines large language models with biomedical knowledge from BioThings Explorer. Tested on three benchmarks built from DrugMechDB (genes, metabolites, and drug-process links), it shows clear accuracy gains compared to using LLMs alone.

Strengths:

The work demonstrates that retrieval improves both small and large models, suggesting cost-efficiency and scalability. This paper also curated multi-scale QA datasets (gene, metabolite, drug) from DrugMechDB provide structured, reproducible evaluation.

Weaknesses:
1. This dual-route design is conceptually sound but too narrow a baseline. A stronger evaluation would compare against other RAG systems (PubMed-based retrieval, BiomedRAG, SPOKE-RAG) instead of just "LLM-only."

We thank the reviewer for this helpful suggestion. Our initial goal was to isolate the contribution of API-driven, structured knowledge graph retrieval by directly comparing BTE-RAG against LLM-only prompting, thereby establishing whether explicit mechanistic retrieval provides advantages beyond prompting alone. However, we have now expanded the evaluation to include GeneGPT (Jin et al, 2024), a representative biomedical RAG framework that integrates external knowledge via NCBI APIs and in-context learning. Specifically, we evaluated both GeneGPT-Full and GeneGPT-Slim alongside BTE-RAG and LLM-only prompting on two benchmarks where GeneGPT is directly applicable: our mechanistic gene benchmark and the GeneTuring gene–disease association dataset (Shang et al, 2025). These additional comparisons, now reported in Sections 3.1 and 3.2, provide a broader RAG baseline and help position BTE-RAG relative to existing biomedical RAG approaches while preserving a controlled comparison focused on mechanistic reasoning.

2. For Entity Recognition step, using pre-annotated entities in benchmarks artificially simplifies the problem. In real-world biomedical QA, entity recognition itself is a major challenge (e.g., ambiguous drug synonyms, rare disease names). Besides, the zero-shot extraction module is described but not evaluated. The paper should report precision/recall of entity recognition to show feasibility beyond curated inputs.

We thank the reviewer for raising this valuable point and to address this, we evaluated our zero-shot entity extraction module on the full set of benchmark questions. As reported in the new Supplementary Table S1, the model achieves high precision and recall (>0.90 across all entity types) and strong joint extraction accuracy (0.89–0.99). These results indicate that automated entity recognition is feasible for an end-to-end BTE-RAG workflow. We also note in the manuscript (Section 2.1, Entity Recognition) that integrating this module into a fully automated pipeline and evaluating it on unannotated biomedical queries is an important direction for future work.

3. No error analysis of BTE retrieval quality is provided. If BTE returns wrong or noisy triples, how often does this mislead the LLM? Adding experiment to show that would strengthen the study.

We thank the reviewer for highlighting the importance of evaluating retrieval quality and its downstream effects on model performance. We conducted a systematic error analysis of all cases in which BTE-RAG underperformed the LLM-only baseline, thereby directly quantifying how often noisy, incomplete, or misleading retrieved triples negatively influenced the LLM. This analysis revealed that most degradation cases were attributable to context dilution (noisy or overly broad retrieved evidence) or evidence coverage gaps (missing mechanistic edges), with only some contributions from filtering errors or derivative mismatches. These results are now presented in the revised Results section (Supplementary Table S6), with full case-level breakdowns provided in our repository at https://github.com/janjoy/BTE-RAG/tree/main/error_analysis.

4. Though the authors used SOTA LLMs, however, the choice of only OpenAI GPT-4o family is narrow. No comparison with open-source biomedical LLMs (e.g., BioGPT, Meditron, PubMedBERT-RAG). Comparison with these model would increase the generalizability

We appreciate the reviewer's suggestion to broaden model comparisons and agree that diversity is important for generalizability. Our model selection was guided by two goals: (i) benchmarking BTE-RAG against strong baselines for biomedical question answering, and (ii) ensuring fair comparison with systems explicitly designed for

Powered by Editorial Manager® and ProduXion Manager® from Aries Systems Corporation

mechanistic reasoning and structured retrieval.
In addition to GPT-4o and GPT-4o-mini, we therefore included GeneGPT (Full and Slim) RAG models, which is specifically designed for biomedical reasoning via structured retrieval from NCBI and BLAST resources. GeneGPT was evaluated alongside BTE-RAG on both an external benchmark (GeneTuring gene–disease association) and one of our mechanistic gene benchmarks, enabling assessment across independent datasets.
We did not include additional biomedical LLMs such as BioGPT or PubMedBERT models because prior large-scale evaluations show that these domain-specific models substantially underperform state-of-the-art general-purpose LLMs on complex biomedical reasoning tasks. For example, the GeneTuring benchmark reports near-zero or very low accuracy for several biomedical LLMs, whereas ChatGPT-based models perform substantially better (Shang et al., 2025). Similarly, MultiMedQA evaluations demonstrate that larger general-purpose models such as GPT-4 outperform smaller domain-specific models by wide margins (Singhal et al., 2025). Consistent with this literature, we selected GPT-4o family models as strong, widely used baselines and focused our evaluation on whether explicit mechanistic retrieval improves reasoning even from competitive starting points. We agree that extending BTE-RAG to additional open-source biomedical models is an important direction for future work.

5. Reliance on one source (DrugMechDB) makes evaluation narrow. The authors should demonstrate performance on at least one independent dataset (e.g., BioASQ, PubMedQA, SPOKE-based tasks) to show broader utility.

We thank the reviewer for this thoughtful comment, and we have now expanded our evaluation to include an independent benchmark, the GeneTuring gene–disease association dataset (which is distinct from DrugMechDB) and is used to assess large language models on genomics knowledge. On this benchmark, we compared LLM-only prompting, BTE-RAG, and two additional biomedical RAG approaches (GeneGPT-Full and GeneGPT-Slim). These results are now reported in Section 3.1 and Figure 2. Together, the inclusion of the GeneTuring benchmark and the DrugMechDB-derived mechanistic tasks demonstrates that BTE-RAG is effective both on an independent association-based dataset and on more complex and mechanistic reasoning problems.

6. Cosine similarity ≥0.9 is arbitrary; should provide ROC/AUC or threshold sensitivity.

We thank the reviewer for this suggestion. To address the concern about threshold selection, we have performed comprehensive threshold sensitivity analyses across 0.85, 0.90, and 0.95 cutoffs for both the metabolite and drug-to-biological-process benchmarks. These analyses, now included in the Results section (sections 3.3 and 3.4), demonstrate that BTE-RAG's relative improvements over LLM-only baselines remain consistent across all examined thresholds. For the metabolite task, we observed robust gains with effect sizes (Cliff's δ) ranging from 0.109 to 0.144 across thresholds. For the drug-to-biological-process task, minimal improvements (Cliff's δ: 0.000–0.018) were consistent across all cutoffs. We also report Wilcoxon signed-rank tests on per-item similarity differences to establish statistical significance.

7. Benchmarks enforce exactly one correct gene, metabolite, or drug per question. Real mechanisms often involve multiple parallel or interacting entities. The single-answer design hides biological complexity and creates an artificial task.

We appreciate this thoughtful comment and fully agree that biological mechanisms often involve multiple parallel or interacting entities. Our choice to use single-answer benchmarks from DrugMechDB was motivated by the need for objective, automated evaluation with clear ground truth that enables rigorous head-to-head comparison between LLM-only and BTE-RAG conditions. While this design necessarily simplifies biological complexity, it provides a reproducible and interpretable baseline for assessing whether knowledge graph context enhances mechanistic reasoning. Importantly, the BTE-RAG framework itself is not limited to single-entity outputs, and the retrieval step already surfaces multiple mechanistic candidates. We have added discussion of this limitation in the Discussion section, noting that real biomedical questions often have multiple valid answers involving pathway and network-based interactions. Future work will extend evaluation to multi-answer or network-based

Powered by Editorial Manager® and ProduXion Manager® from Aries Systems Corporation

	formats that better capture the parallelism and complexity of real biological systems.<br><br>8. Ground truth relies on exact HGNC, CHEBI, or DrugBank IDs. Why the ambiguities (synonyms, deprecated IDs, overlapping terms) are filtered out rather than addressed? This may bias the dataset toward easier, cleaner cases.<br><br>We appreciate this concern and would like to clarify our design. For the gene benchmark, we used HGNC symbols rather than IDs, as symbols are generally more natural for LLMs and reduce confusion from numerical identifiers. For metabolites and drugs, we did not rely on CHEBI or DrugBank IDs; instead, we evaluated model outputs against names using cosine similarity of BioBERT-STSB text embeddings, a metric that captures graded lexical and semantic overlap rather than strict string matching. This approach inherently accommodates synonyms and overlapping terms while avoiding penalization for minor wording differences. Our primary goal in this work was to establish a fair comparison between LLM-only and BTE-RAG, and therefore we prioritized clarity and consistency in evaluation. In future iterations, we plan to extend the framework with explicit synonym resolution and deprecated mappings to further test robustness under noisier, real-world conditions.<br><br>9. The paper cited recent biomedical RAG systems such as BiomedRAG, GeneTuring but didn't compare with them (e.g., BiomedRAG). BioRAG (2024) is also highly relevant. These works are highly relevant baselines, showing retrieval from knowledge graphs, APIs, or literature, and including them in comparison would better position BTE-RAG within the current state of the art and highlight its unique contributions.<br><br>Thank you for this suggestion, we have now expanded our empirical evaluation to include GeneGPT, a representative biomedical RAG framework that integrates structured knowledge via NCBI (National Center for Biotechnology Information) APIs and in-context learning (Jin et al., 2024). Specifically, we evaluated both the GeneGPT-Full and GeneGPT-Slim configurations alongside LLM-only prompting and BTE-RAG on the GeneTuring gene–disease association benchmark as well as on our newly introduced mechanistic gene benchmark. These results are now reported in Sections 3.1 and 3.2, with detailed statistical analyses provided in the main text and supplementary materials. We agree that broader multi-model comparisons are valuable, and incorporating additional biomedical RAG systems and related approaches is an important direction for future work.
**Additional Information:**	
**Question**	**Response**
Are you submitting this manuscript to a special series or article collection?	No
**Experimental design and statistics**<br><br>Full details of the experimental design and statistical methods used should be given in the Methods section, as detailed in our Minimum Standards Reporting Checklist. Information essential to interpreting the data presented should be made available in the figure legends.<br><br>Have you included all the information requested in your manuscript?	Yes
**Resources**	Yes

A description of all resources used, including antibodies, cell lines, animals and software tools, with enough information to allow them to be uniquely identified, should be included in the Methods section. Authors are strongly encouraged to cite Research Resource Identifiers (RRIDs) for antibodies, model organisms and tools, where possible.<br><br>Have you included the information requested as detailed in our Minimum Standards Reporting Checklist?	
**Availability of data and materials**<br><br>All datasets and code on which the conclusions of the paper rely must be either included in your submission or deposited in publicly available repositories (where available and ethically appropriate), referencing such data using a unique identifier in the references and in the "Availability of Data and Materials" section of your manuscript.<br><br>Have you have met the above requirement as detailed in our Minimum Standards Reporting Checklist?	Yes
GigaScience has policies and guidelines in place for the use of generative AI-writing tools such as ChatGPT. If you have used such writing tools to assist with writing the manuscript this must be declared and cited in the text. Authors should not list AI-writing tools and other AI-assisted technologies as an author or co-author and should acknowledge that they are fully responsible for text generated or refined by AI-writing tools.<p><br><br>A summary of use (particularly in the introduction or among methods) needs to be included at the end of the paper, and the outputs should also be included as a supplementary file hosted in GigaDB or other open repositories. Please <a href=https://academic.oup.com/gigascience/pages/editorial_policies_and_reporting_	Yes

standards target="_new" > read our guidelines for more information. <p><br><br>By submitting to GigaScience, you are aware of the journal's AI-writing tools policy, and if you have declared use of such tools below, you have acknowledged this where appropriate in your manuscript and have made a summary of use and outputs available. <p><br><b>AI-assisted writing tools have been used in the preparation of this manuscript?	

Manuscript
Click here to access/download;Manuscript;Joy_et_al_manuscript_Jan 15.docx

Federated Knowledge Retrieval Elevates Large Language Model Performance on Biomedical Benchmarks

Janet Joy[1,2], Andrew I. Su[1,2]

[1]Department of Integrative Structural and Computational Biology, Scripps Research, La Jolla, CA, USA.

[2]Correspondence: Janet Joy (jjoy@scripps.edu) and Andrew I. Su (asu@scripps.edu)

Janet Joy [0000-0002-8871-0765]

Andrew I. Su [0000-0002-9859-4104]

Main Figures: 5
Supplemental Figures: 13
Supplemental Tables: 7

Abstract

Background

Large language models (LLMs) have significantly advanced natural language processing in biomedical research, however, their reliance on implicit, statistical representations often results in factual inaccuracies or hallucinations, posing significant concerns in high-stakes biomedical contexts.

Results

To overcome these limitations, we developed BTE-RAG, a retrieval-augmented generation framework that integrates the reasoning capabilities of advanced language models with explicit mechanistic evidence sourced from BioThings Explorer, an API federation of more than sixty authoritative biomedical knowledge sources. We systematically evaluated BTE-RAG in comparison to traditional LLM-only methods across three benchmark datasets that we created from DrugMechDB. These datasets specifically targeted gene-centric mechanisms (798 questions), metabolite effects (201 questions), and drug–biological process relationships (842 questions). On the gene-centric task, BTE-RAG increased accuracy from 51% to 75.8% for GPT-4o mini and from 69.8% to 78.6% for GPT-4o. In metabolite-focused questions, the proportion of responses with cosine similarity scores of at least 0.90 rose by 82% for GPT-4o mini and 77% for GPT-4o. While overall accuracy was consistent in the drug–biological process benchmark, the retrieval method enhanced response concordance, producing a greater than 10% increase in high-agreement answers (from 129 to 144) using GPT-4o. We additionally evaluated BTE-RAG alongside GeneGPT-based models on the GeneTuring gene–disease association benchmark and on our mechanistic gene benchmark, demonstrating that the BTE-RAG layer consistently improves accuracy relative to alternative approaches.

Conclusion

Federated knowledge retrieval provides transparent improvements in accuracy for large

language models, establishing BTE-RAG as a valuable and practical tool for mechanistic exploration and translational biomedical research.

1 Introduction

Large language models (LLMs) have rapidly advanced the state of natural-language processing, reaching or surpassing expert performance across a wide range of biomedical tasks, including cell type annotation, protein-structure prediction and automated synthesis of clinical-trial results [1–6]. However, the underlying generative methodology of these models, which sequentially predict tokens based on statistical patterns learned from massive text corpora, renders them susceptible to hallucinations, defined as outputs that are syntactically fluent yet factually incorrect [7,8]. Such inaccuracies pose significant risks in biomedicine, where even minor errors can misdirect research efforts, delay critical therapeutic discoveries, or compromise patient safety [7,9–11]. Indeed, recent assessments underscore that hallucination rates remain too high for safe and effective deployment in clinical and research-intensive environments [12,13].

Efforts to mitigate these hallucinations through domain-specific pre-training and prompt engineering have yielded only incremental improvements, as these approaches continue to embed knowledge implicitly within opaque model parameters and fail to reliably surface evidence provenance [14–16]. Retrieval-augmented generation (RAG) has emerged as a promising solution, explicitly grounding model-generated responses by dynamically incorporating external, verifiable evidence into prompts [17–19]. Within biomedical question-answering contexts, RAG approaches consistently reduce hallucinations and elevate factual accuracy compared to parameter-only models. Nonetheless, the efficacy of RAG hinges critically on the precision, comprehensiveness, and currency of the retrieved contextual evidence [20–22].

Knowledge graphs (KGs) are particularly compelling resources for RAG because they explicitly represent biological entities and their relationships, support multi-hop mechanistic reasoning, and maintain persistent identifiers that simplify provenance tracking [23–26]. Yet most biomedical KGs are tuned to a narrow slice of biology (for example, protein–protein interactions) or require extensive curation to remain current, limiting their utility for cross-domain mechanistic reasoning. To address these challenges, BioThings Explorer (BTE) integrates and federates 61 authoritative biomedical APIs into a continuously updated meta-knowledge graph that encompasses genes, pathways, drugs, diseases, phenotypes, and more [27]. The API-centric framework of BTE returns structured JSON triples annotated with semantic types and evidence citations from reputable biomedical databases such as Gene Ontology, DrugBank, and Pubmed central using Translator Reasoner API (TRAPI) specification [28–30].

Here, we introduce BTE–RAG (BioThings Explorer–Retrieval-Augmented Generation), a novel framework that integrates the conversational fluency and reasoning capabilities of advanced LLMs with the explicit, multi-domain mechanistic knowledge captured by BTE. BTE–RAG dynamically executes targeted, query-focused graph traversals to retrieve concise, mechanistically pertinent evidence, formulates this evidence into declarative context statements, and augments model prompts accordingly.

To rigorously assess the performance of BTE-RAG in biomedical question answering, we systematically created three specialized benchmark datasets from DrugMechDB, a curated knowledge base containing 5,666 expert-annotated mechanistic pathways with literature validation [31]. These datasets consist of gene-centric (n = 798), metabolite-centric (n = 201), and drug-centric (n = 842) question–answer pairs, each explicitly reflecting the causal flow from drug through intermediate biological nodes to disease outcomes. Across all three DrugMechDB-derived benchmarks, BTE–RAG robustly improves factual grounding, accelerates convergence to correct responses over diverse biomedical entities relative to an LLM-only baseline.

Collectively, these findings establish BTE–RAG as a powerful, practical tool for reducing hallucination risks and enhancing mechanistic clarity, significantly advancing the transparency, reliability, and utility of language model-driven biomedical discovery and clinical decision-making.

2 Materials & Methods

2.1 BTE–RAG Framework and Baseline Comparison

The BTE–RAG framework systematically compares two distinct inference routes to evaluate the impact of structured, mechanistic context on large language model (LLM) outputs (Figure 1A). The first inference route, labeled "LLM-only," directly submits user-generated questions to the language model without external context augmentation. The second route, labeled "BTE–RAG," integrates structured mechanistic evidence retrieved from BioThings Explorer prior to submitting an enriched, evidence-supported prompt to the same language model. This dual-path design allows rigorous evaluation of how explicitly retrieved context influences both answer accuracy and the factual grounding of model-generated responses. The BTE–RAG architecture comprises three key phases: entity recognition, knowledge-graph-based retrieval via BTE, and generative inference utilizing context-augmented LLM prompting.

Entity Recognition

The retrieval phase begins with precise identification of biomedical entities mentioned within each input question. For the current benchmarks, entities such as drugs, diseases, metabolites, and biological processes were pre-annotated and standardized to established knowledge graph identifiers, enabling automated recognition at runtime. Additionally, the framework includes a zero-shot entity extraction module that leverages a specialized task-oriented prompting approach. This module is currently optimized for retrieving drugs, diseases and biological processes from queries, with potential to extend extraction capabilities to include other biomedical entities as needed. To validate the feasibility of automated entity extraction for real-world deployment, we evaluated this zero-shot GPT-3.5-based extraction module on all benchmark questions. The extractor achieved precision and recall >0.90 for all entity types, with joint accuracy (correctly extracting all required entities per question) ranging from 0.89-0.99 across the three benchmarks (Supplementary Table S1). These results show that automated entity extraction works reliably, though integrating it into the complete system and testing real-world unannotated questions remains important future work.

Knowledge Graph Retrieval

Identified biomedical entities are translated into structured queries interfacing directly with BTE. BTE integrates 61 authoritative biomedical databases under a unified knowledge graph schema, accessible via the programmatic API endpoint (/v1/query). Each query to BTE specifies an input entity (e.g., disease, drug, or biological process) along with desired output entity categories, following the TRAPI query format. In response, BTE returns structured JSON data that includes a detailed knowledge graph containing two key components: "nodes," which describe biomedical entities along with their semantic categories and standardized names; and "edges," which specify the explicit relationships (predicates) between pairs of entities, supplemented by provenance details indicating the primary knowledge sources.

For each benchmark dataset, targeted queries were structured to retrieve mechanistically relevant context. Specifically, in the gene-centric benchmark, queries separately utilized disease and drug entities to retrieve directly linked gene and protein nodes. In the metabolite-centric benchmark, disease and chemical (drug) entities were queried independently to identify connected biochemical entities. For the drug–biological process benchmark, separate queries using disease entities and biological process entities were conducted to retrieve associated chemical entities (drugs). Upon receiving the structured knowledge graph responses from BTE, both node and edge information were systematically processed. Nodes were extracted along with their semantic categories and descriptive names, while edges were parsed to identify subject-object pairs, predicates, and associated primary knowledge sources. Nodes and edges were subsequently merged to construct coherent statements that succinctly describe each mechanistic relationship (e.g., "drug X inhibits gene Y"). These concise, natural-language context statements collectively formed the mechanistic evidence provided to the language models during the generative inference phase, significantly enhancing the transparency, interpretability, and accuracy of the generated outputs. Supplementary Figure S1 provides a detailed schematic illustrating the complete BTE–RAG pipeline workflow, demonstrating a representative query and the subsequent processing and integration steps.

Context Selection

Two distinct evidence-inclusion strategies were systematically assessed for each question. The first strategy incorporates the entire set of sentences retrieved by BTE, leveraging the extensive 128,000-token context window of GPT-4o [32]. The second strategy employs sentence-level cosine similarity filtering using ‘S-PubMedBert-MS-MARCO’ embeddings, retaining only sentences whose similarity scores with the query exceed a predefined percentile threshold [33]. Running these two strategies concurrently enables a direct evaluation of the impact of comprehensive versus selectively pruned contextual evidence under identical experimental conditions.

Generative Inference

For the generative phase, selected context sentences and the original query were concatenated to form an enriched prompt submitted to both GPT-4o and GPT-4o-mini models. Models were configured deterministically (temperature set to 0) to produce

reproducible outputs. Parallel runs of the LLM-only baseline used identical questions without the BTE-derived context. To streamline downstream analyses and ensure objective comparisons, language models were instructed explicitly to output structured JSON responses devoid of extraneous explanatory text. Detailed system prompts are shown in Supplementary Table S7.

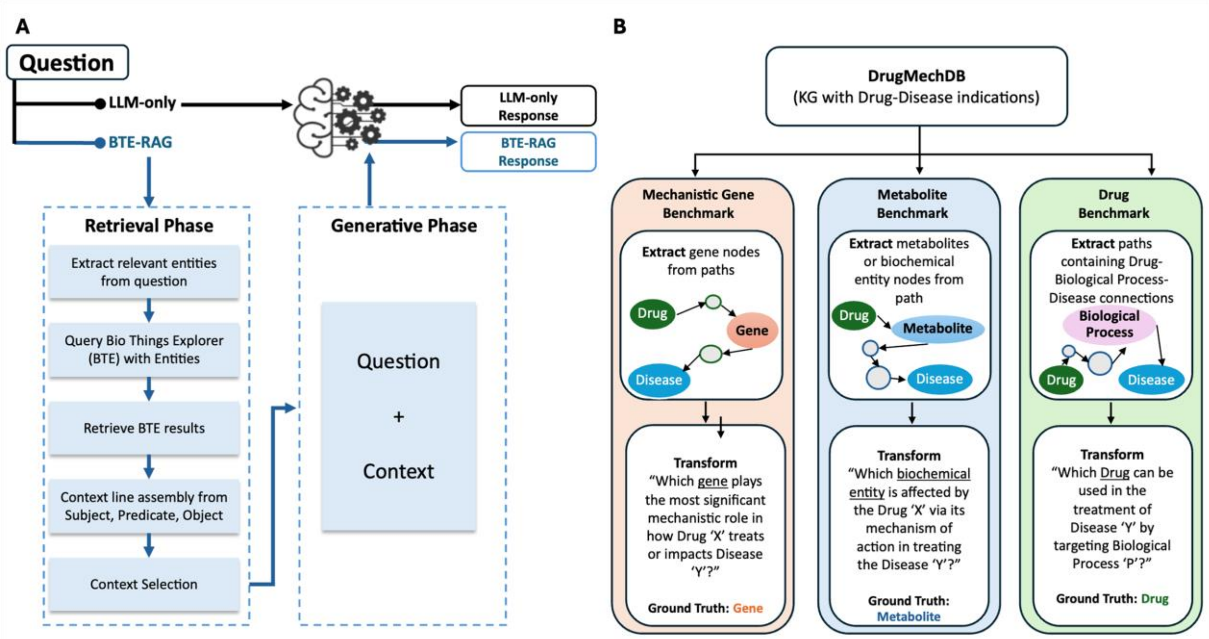

Figure 1: Retrieval-Augmented Generation workflow and derivation of mechanistic evaluation benchmarks.
(A) Schematic of the BTE-RAG pipeline, which augments large language model (LLM) responses with context retrieved from the BioThings Explorer (BTE) knowledge graph. In the LLM-only pathway, the model generates a response using only the input question. In contrast, BTE-RAG operates in two phases: a Retrieval Phase, where relevant entities are extracted from the question and queried against BTE to collect mechanistically relevant subject–predicate–object triples, and a Generative Phase, where this curated context is appended to the input question and passed to the LLM. The resulting outputs: LLM-only or BTE-RAG, can be directly compared to assess the impact of knowledge-augmented generation.
(B) Construction of benchmark datasets from DrugMechDB, a curated biomedical knowledge graph of drug–disease mechanisms. Directed paths connecting a drug to a disease were mined and transformed into structured questions targeting different mechanistic facets: (i) gene nodes (Mechanistic Gene Benchmark), (ii) biochemical entities or metabolites (Metabolite Benchmark), and (iii) drug–biological process–disease paths (Drug Benchmark). Each benchmark provides paired questions and gold-standard labels for rigorous, domain-specific evaluation of retrieval-augmented generation.

Evaluation

Generated outputs were benchmarked against curated ground-truth annotations from the constructed mechanistic datasets. For entity-specific tasks (e.g., gene identification), correctness was evaluated via exact, case-insensitive string matching. For semantically

nuanced responses (e.g., metabolites and drugs), BioBERT-based embeddings ('BioBERT-mnli-snli-scinli-scitail-mednli-stsb') quantified the semantic similarity between model-generated outputs and reference answers [34]. Answers surpassing a predetermined similarity threshold were classified as accurate. Collectively, these standardized evaluation methodologies ensure scalable, objective, and reproducible assessment of the fidelity and biological coherence of model predictions, rigorously testing the utility and impact of knowledge graph-enhanced prompting in biomedical reasoning contexts.

Embedding and Scoring Models

We use pritamdeka/S-PubMedBert-MS-MARCO, a PubMedBERT model fine-tuned on the MS MARCO passage-ranking task [35]. This model is optimized for high-recall biomedical information retrieval, having been trained on large-scale query-passage pairs to identify relevant content across lexically diverse API outputs. Its passage-ranking specialization makes it well-suited for the initial filtering stage where we prioritize recall over precision to avoid prematurely discarding potentially relevant knowledge graph paths.

Similarity scoring: For semantic similarity evaluation, we use pritamdeka/BioBERT-mnli-snli-scinli-scitail-mednli-stsb, which extends BioBERT with additional fine-tuning on natural language inference (SNLI, MNLI, SciNLI, MedNLI) and semantic textual similarity (STS-B, SciTail) datasets [36]. This multi-task training enhances its ability to capture nuanced semantic relationships including synonymy, paraphrasing, and entailment, critical for accurately assessing whether generated answers match gold-standard mechanistic explanations despite surface-level differences in phrasing.

Using distinct models for retrieval and evaluation aligns each component with its functional requirements: S-PubMedBERT-MS-MARCO prioritizes finding all potentially relevant passages (high recall), while the BioBERT-based model provides precise semantic equivalence judgments (high precision).

2.2 External Baseline Methods and Benchmarks

In addition to evaluating BTE–RAG, we compared against established biomedical reasoning frameworks and benchmarks commonly used to assess large language models in genomics.

2.2.1 GeneGPT Models

We evaluated GeneGPT, a biomedical reasoning framework that enables large language models to interact with structured biomedical knowledge through NCBI (National Center for Biotechnology Information) Web APIs using in-context learning [37]. GeneGPT couples curated API documentations and usage demonstrations with an inference procedure that integrates API calls directly into the model's decoding process, allowing external knowledge to be retrieved and incorporated during answer generation.

In this study, we evaluated two GeneGPT configurations as described in the original GeneGPT framework. The GeneGPT-Full configuration (denoted as 111111) includes all available API documentations (Dc.1–Dc.2) and usage demonstrations (Dm.1–Dm.4)

within the in-context prompt. The GeneGPT-Slim configuration (denoted as 001001) uses a reduced prompt consisting only of Demonstrations Dm.1 and Dm.4, thereby limiting contextual information while retaining core API usage examples.

To ensure a fair comparison with other methods evaluated using GPT-4o family models, all GeneGPT variants were adapted to use GPT-4o-mini or GPT-4o as the underlying language model, and the context window was expanded to 100,000 tokens, exceeding the original Codex-based implementation (18k tokens).

2.2.2 GeneTuring Gene–Disease Association Benchmark

To benchmark performance on a standardized genomics task, we used the GeneTuring benchmark, a curated question-answering dataset designed to evaluate large language models on genomics knowledge [38]. From GeneTuring, we selected the functional analysis category, specifically focusing on the gene–disease association module, which assesses whether a model can correctly identify genes associated with a given disease.

Each query was reformatted into a standardized question template of the form: "What is the gene related to [disease]?" Model outputs were evaluated by comparing the predicted gene against the corresponding ground-truth gene annotations provided by GeneTuring. Predictions were scored as correct if the model-generated gene matched any of the accepted ground-truth answers for a given question, and incorrect otherwise, yielding a binary outcome suitable for accuracy-based evaluation.

2.3 Datasets from DrugMech Data Base

Construction of Mechanistic Question–Answer Benchmarks from DrugMechDB

DrugMechDB is a rigorously curated biomedical knowledge graph designed to represent therapeutic mechanisms through explicit stepwise paths. These pathways originate from drug nodes, traverse biologically meaningful intermediate entities, and culminate at disease nodes, collectively delineating mechanisms underlying drug–disease interactions [31]. The current version of DrugMechDB contains 5,666 curated mechanistic pathways, providing comprehensive coverage for 4,583 distinct drug–disease indications. Each node within DrugMechDB is systematically mapped to a standardized Biolink category and anchored to stable identifiers, while each relationship (edge) is annotated with a controlled predicate [39]. This structured, granular, and provenance-rich resource enables robust benchmarking of computational models focused on mechanistic inference rather than simple associative or co-occurrence patterns.

To comprehensively evaluate the BTE–RAG framework across multiple levels of biological resolution, DrugMechDB was systematically transformed into three complementary mechanistic question–answer (QA) benchmarks, each highlighting a distinct biological focus: genes, metabolites, and drugs (Figure 1B).

Gene-Centric Benchmark: Mechanistic pathways were initially filtered to retain those containing exactly one internal node annotated as a Gene entity. Gene identifiers were resolved into standardized HGNC symbols using MyGene.info services; pathways containing deprecated or ambiguous identifiers were systematically excluded [40]. Each remaining mechanistic pathway was converted into a structured question of the form:

"Which gene plays the most significant mechanistic role in how Drug 'X' treats or impacts Disease 'Y'?" The corresponding HGNC gene symbol served as the definitive ground truth. Following deduplication across different indications, this dataset comprised 798 unique QA pairs.

Metabolite-Centric Benchmark: To capture downstream biochemical effects, pathways exclusively containing taxonomic relationships (such as “subclass” predicates) were removed to ensure mechanistic specificity. Selected pathways included exactly one metabolite node, identified specifically by filtering node identifiers prefixed with "CHEBI:" to denote biochemical entities. Records containing multiple mechanistic pathways were excluded to maintain dataset simplicity and clarity. Each qualifying pathway was formulated into the structured question: "Which biochemical entity is affected by Drug 'X' via its mechanism of action in treating Disease 'Y'?" The metabolite node identified via CHEBI identifiers served as the ground truth answer, yielding a final dataset of 201 unique QA pairs.

Drug-Centric Benchmark: A third benchmark dataset was developed to evaluate the ability of computational models to infer therapeutic agents when provided with a disease and a mediating biological process. Pathways were selected specifically if they included exactly one BiologicalProcess node, and drugs lacking resolvable identifiers from DrugBank or MESH databases were excluded to ensure accurate and standardized identification. Each qualifying path was structured into the question: "Which drug can be used in the treatment of Disease 'Y' by targeting Biological Process 'P'?" The corresponding drug node served as the ground truth. After thorough harmonization and stringent quality control measures, this benchmark comprised 842 unique QA pairs.

The resulting benchmarks thus offer a robust, multiscale evaluation platform specifically designed to probe the mechanistic inference capabilities of knowledge-graph-augmented language models comprehensively and rigorously.

2.4 Use of Large Language Models:

All natural-language processing steps were carried out with two OpenAI models, GPT-4o-mini (snapshot 2024-07-18) and GPT-4o (snapshot 2024-08-06) [32,41]. Both models were invoked through the OpenAI API. The temperature parameter was fixed at 0.0 for every request, thereby forcing deterministic decoding and facilitating reproducible evaluation. Each model accepts up to 128,000 input tokens and can return a maximum of 16,384 completion tokens. Although GPT-4o-mini is substantially smaller in parameter count than GPT-4o, both models share the same context window size, permitting a controlled comparison of model capacity while holding prompt length constant [32,41]. At the time the experiments were executed, GPT-4o-mini was priced at 0.15 USD per million input tokens and 0.60 USD per million output tokens [42]. The corresponding prices for GPT-4o were 2.50 USD and 10.00 USD, respectively [43]. Model versions were pinned by explicit snapshot identifiers to eliminate the possibility of version drift during the study period. ChatGPT was used to assist with grammar correction and to improve conciseness in the manuscript.

Prompt engineering

Each request began with a concise system prompt defining the model's role [44–46]. Two distinct system prompts were prepared per dataset: one for the standalone LLM baseline, and one tailored for the retrieval-augmented BTE–RAG workflow. Queries were provided directly to the model without additional contextual examples, employing a zero-shot prompting approach. To facilitate efficient and accurate downstream processing, the model was instructed to produce responses strictly in a predefined JSON format, omitting supplementary explanatory text.

3 Results

We developed BTE–RAG, a retrieval-augmented generation framework designed to enhance large language models (LLMs) by integrating mechanistic evidence from BioThings Explorer (BTE), a federated biomedical knowledge graph. BTE–RAG embeds structured, graph-derived context into prompts to improve mechanistic accuracy, ensure explicit provenance, and facilitate higher-order reasoning. We benchmarked the performance of BTE–RAG across four distinct biomedical reasoning tasks: (1) GeneTuring's gene-disease task, (2) gene identification from drug-disease mechanisms, (3) drug–metabolite-disease interactions, and (4) drug–biological-process-disease relationships. For gene-focused tasks (benchmarks 1 and 2), we compared BTE-RAG against three baseline approaches: LLM-only prompting, GeneGPT-Full, and GeneGPT-Slim. For metabolite and drug-biological process benchmarks we compared BTE-RAG against LLM-only prompting.

3.1 GeneTuring Gene-Disease Association Benchmark

To establish baseline performance on a standardized biomedical association task, we evaluated BTE-RAG on the GeneTuring gene-disease association dataset alongside existing biomedical RAG and prompting-based approaches [38]. This benchmark focuses on identifying associations between genes and diseases, providing a complementary evaluation to our mechanistic reasoning benchmarks. We compared four approaches: (1) LLM-only prompting, (2) BTE-RAG, (3) GeneGPT-Full, and (4) GeneGPT-Slim.

Across both model scales, BTE-RAG achieved the highest accuracy, substantially outperforming all baselines (Figure 2). On GPT-4o-mini, BTE-RAG increased accuracy from 33 % to 77 %, while on GPT-4o performance rose from 56 % to 80 %, despite the stronger baseline of the larger model. In both cases, GeneGPT-Full and GeneGPT-Slim achieved intermediate accuracy, indicating that API-level retrieval alone provides only partial gains.

All pairwise comparisons between BTE-RAG and baseline methods were statistically significant (Supplementary Table S2). With GPT-4o-mini, BTE-RAG's advantage over LLM-only was 44 percentage points , over GeneGPT-Full was 48 points and over GeneGPT-Slim was 45 points .With GPT-4o, the corresponding advantages were 24 points, 21 points, and 16 points.

Contingency-table analysis further revealed that these improvements were driven by asymmetric error correction (Supplementary Figure S2). For GPT-4o-mini, BTE-RAG corrected 45–49 gene–disease associations that baseline methods failed, while introducing only 1–2 errors on questions answered correctly by the baselines. For GPT-

4o, BTE-RAG corrected 21–27 additional associations with only 3–5 reversals. These results indicate that federated knowledge graph retrieval provides substantially more relevant and accurate biomedical context than either unstructured prompting or API-based retrieval alone for gene–disease association tasks.

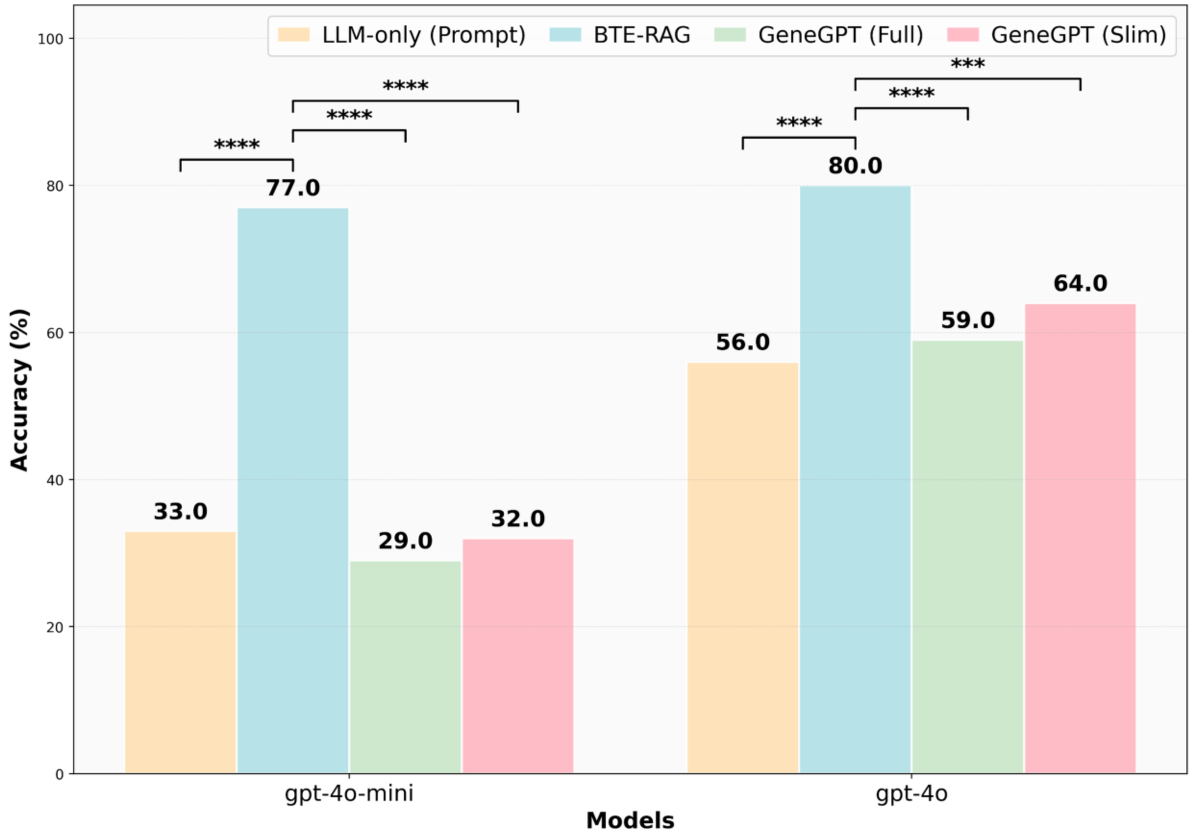

Figure 2: Method comparison on the GeneTuring gene–disease association benchmark.
Accuracy is shown for four approaches: LLM-only prompting, BTE-RAG, GeneGPT-Full, and GeneGPT-Slim, evaluated on the set of GenTuring's gene–disease association benchmark. Results are reported separately for the compact gpt-4o-mini and the larger gpt-4o models. For both the models, incorporating the BTE-RAG retrieval layer substantially increased accuracy relative to the LLM-only baseline. Bars indicate percentage accuracy. Statistical significance was assessed using paired McNemar's tests, comparing BTE-RAG against each baseline within the same model. Significance levels are indicated as * $p < 0.05$, ** $p < 0.01$, *** $p < 0.001$, and **** $p < 1 \times 10^{-4}$. (exact statistics and flip counts reported in Supplementary Table S2 and Figure S2).

3.2 Mechanistic Gene Prediction

We next assessed the impact of retrieval-augmented and structured knowledge-based inference on gene-level mechanistic reasoning, using 798 curated mechanistic drug–disease associations from DrugMechDB. Each query was formulated as: "Which gene plays the most significant mechanistic role in how Drug X treats or impacts Disease Y?" Both models, GPT-4o-mini and GPT-4o were evaluated across four approaches.

Under the LLM-only condition, GPT-4o-mini achieved an accuracy of 51.0% on the mechanistic gene benchmark (Figure 3). Incorporating BTE-RAG substantially increased accuracy to 75.8%, corresponding to an absolute improvement of 24.8 percentage points, which was highly significant by paired analysis (McNemar $p < 0.0001$; Supplementary Table S3). In contrast, GeneGPT-Full and GeneGPT-Slim achieved lower accuracies of 37.2% and 45.2%, respectively. Direct pairwise comparisons confirmed that BTE-RAG significantly outperformed both GeneGPT variants across all tests (Supplementary Table S3).

A similar pattern was observed for the larger GPT-4o model. LLM-only prompting achieved an accuracy of 69.8%, which increased to 78.6% with BTE-RAG, yielding an absolute gain of 8.8 percentage points (McNemar $p < 0.0001$; Figure 3, Supplementary Table S3). As with GPT-4o-mini, GeneGPT-Full and GeneGPT-Slim exhibited substantially lower performance (42.7% and 44.0%, respectively), and BTE-RAG significantly outperformed both structured GeneGPT configurations in all pairwise

comparisons (Supplementary Table S3).

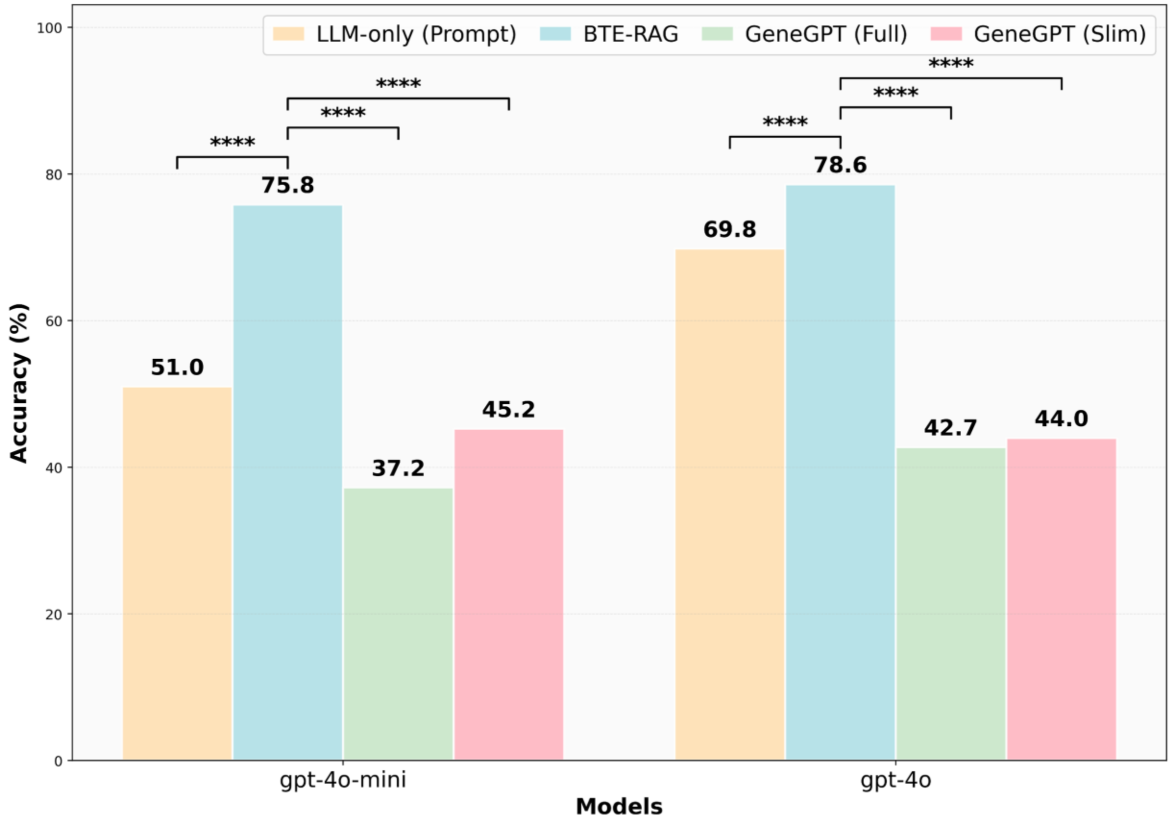

Figure 3: Retrieval-augmented generation with BTE-RAG markedly improves factual accuracy of gene-centric benchmark using GPT-4o models. Shown is the accuracy of four inference strategies applied to a mechanistic gene benchmark using gpt-4o-mini and gpt-4o. Across both model scales, BTE-RAG outperforms LLM-only prompting and GeneGPT-based approaches, demonstrating the advantage of query-conditioned retrieval for gene-level mechanistic reasoning. Bars indicate percentage accuracy. Statistical significance was assessed using paired McNemar's tests, comparing BTE-RAG against each baseline within the same model. Significance levels are indicated as * $p < 0.05$, ** $p < 0.01$, *** $p < 0.001$, and **** $p < 1 \times 10^{-4}$. (Supplementary Table S3 and Figure S3).

Contingency-table analyses revealed that these gains were driven by strongly asymmetric error corrections. For GPT-4o-mini, BTE-RAG converted 245 previously incorrect predictions to correct, with only 47 reversals from correct to incorrect, while for GPT-4o, 119 incorrect predictions were corrected with 49 reversals (Supplementary Figure S3). Similar asymmetries were observed when comparing BTE-RAG against both GeneGPT-Full and GeneGPT-Slim, accounting for the highly significant McNemar statistics across all pairwise comparisons.

Because knowledge-graph queries can return superfluous triples, we evaluated a simple similarity-based pruning strategy. Specifically, both the user queries and the context statements were embedded using the sentence embedding model 'S-PubMedBert-MS-MARCO' [33]. Context statements were then ranked based on cosine

similarity scores relative to the embedded query, and those statements falling within the lowest 10% similarity scores were removed to retain only the most relevant context lines. This lightweight filtering strategy preserved, and in some cases slightly enhanced performance across all evaluated accuracy metrics (Supplementary Figure S4A, S5A), suggesting that excluding the least relevant context statements can beneficially impact the accuracy of gene-level reasoning tasks.

Together, these results demonstrate that retrieval-augmented mechanistic context provided by BTE-RAG consistently and substantially improves gene-level reasoning, outperforming both LLM-only prompting and structured GeneGPT-based approaches across base model scales. These gains are particularly pronounced for smaller models such as GPT-4o-mini, where retrieval markedly amplifies mechanistic inference capabilities, but remain evident even for the larger GPT-4o model. The observed improvements indicate that state-of-the-art language models retain latent mechanistic knowledge gaps that can be effectively bridged through the integration of curated biomedical knowledge graphs and the selective inclusion of relevant contextual evidence.

3.3 Prediction of Drug–Metabolite Relationships

To gauge whether retrieval augments the mechanistic fidelity of metabolite-level reasoning, we posed 201 queries of the form "Which biochemical entity is affected by Drug X via its mechanism of action in treating Disease Y?" using the DrugMechDB-derived Drug → Metabolite → Disease paths. Because metabolite names are much less standardized than gene names, we scored the answer quality by computing a semantic concordance between each model answer and the gold standard metabolite. Semantic concordance was based on cosine similarity of text embeddings using the BioBERT-STSB text embedding model, a metric that rewards graded lexical and semantic overlap rather than exact string identity [34].

Rank-ordered similarity curves in Figure 4A immediately reveal the effect of augmentation: for both gpt-4o-mini (orange) and gpt-4o (blue), the BTE-RAG trace (solid line) departs from the prompt-only baseline (dashed line) after ~130 ranked questions (cosine ≈ 0.70) and widens steadily, nearly doubling the number of answers that reach the high-fidelity zone (cosine ≥ 0.90). Paired per-item similarity analyses demonstrated a consistent improvement with retrieval augmentation across both model scales. BTE-RAG increased mean similarity by 0.066 (95% CI: 0.031–0.099) for GPT-4o-mini and by 0.063 (95% CI: 0.030–0.095) for GPT-4o. These improvements were statistically significant by Wilcoxon signed-rank tests ($p < 0.001$ for both models). Effect size estimation using Cliff's delta (δ) indicated a small effect for GPT-4o-mini (δ = 0.239, 95% CI: 0.139–0.338) and a negligible effect for GPT-4o (δ = 0.065, 95% CI: −0.040–0.169), indicating that the smaller model benefited more substantially from retrieval augmentation.

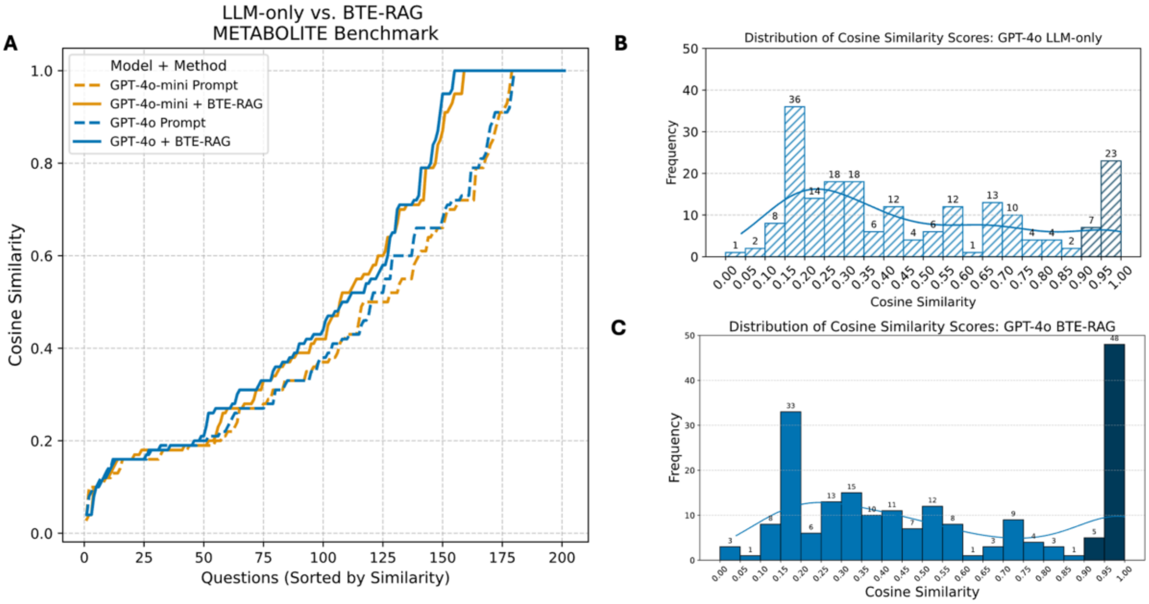

Figure 4: Retrieval-augmented context increases semantic concordance with ground-truth metabolites.
(A) Cosine-similarity scores between each generated answer and the corresponding reference metabolite (sentence-transformer embeddings; see Methods) are plotted for all 201 questions in the Metabolite Benchmark, ordered from lowest to highest similarity. Dashed traces represent the LLM-only baseline, whereas solid traces include BioThings Explorer (BTE) retrieval-augmented context. Orange curves denote gpt-4o-mini; blue curves denote gpt-4o. For both model sizes, BTE-RAG systematically shifts the similarity distribution upward, indicating improved semantic alignment with the curated biochemical ground truth.
(B) Score distribution GPT-4o, LLM-only. Histogram of cosine-similarity scores for GPT-4o answers generated without external context. Bar heights and numeric labels denote the number of questions (n = 201) falling in each bin; the overlaid KDE line summarizes the distribution.
(C) Score distribution GPT-4o + BTE-RAG. Same format as panel B but for GPT-4o answers generated with BTE-RAG's context. The right-shifted, more peaked distribution highlights the improvement in semantic alignment achieved by retrieval-augmented generation.

Histograms for the prompt-only condition (Figure 4B, gpt4o; Supplementary Figure S6, gpt-4o-mini) reveal a pronounced left-skew: both gpt-4o-mini and gpt-4o peak in the 0.15–0.30 similarity bins, with medians below 0.30. Only 15 % of answers fall in the high-similarity regime (≥ 0.90), indicating that the LLMs frequently retrieve metabolites that are semantically distant from the curated ground truth.

To validate this threshold choice, we examined sensitivity across nearby cutoffs (0.85, 0.90, and 0.95) (Supplementary Table S4). For GPT-4o-mini, BTE-RAG increased high-fidelity answers from 15.4% to 26.4% at the 0.85 threshold, from 13.9% to 25.4% at 0.90 and from 11.9% to 23.4% at 0.95. GPT-4o showed similar patterns: 16.4% to

27.4% at 0.85, 14.9% to 26.4% at 0.90, and 11.4% to 25.9% at 0.95. The consistent gains across all thresholds confirm that retrieval augmentation robustly enhances high-fidelity predictions regardless of the specific cutoff selected.

Appending BTE evidence shifts the distributions rightward across similarity bins (Figure 4C (gpt-4o), Supplementary Figure S7, S8). For GPT-4o-mini, applying a stringent context similarity threshold (>80th percentile) increased the number of high-fidelity answers (cosine similarity 0.90–1.00) from 28 to 51 (+82%). Similarly, GPT-4o exhibited an increase from 30 to 53 (+77%) under the same conditions. Simultaneously, counts in the mid-similarity interval (0.40–0.70) contract (Supplementary Figure S7, S8), confirming that retrieval largely converts borderline predictions into highly concordant hits rather than merely redistributing low-score failures.

Because voluminous context can inflate token budgets, we assessed performance when progressively discarding lower-ranked context lines (10th to 90th percentile cut-offs). Rank-ordered similarity traces (Supplementary Figure S9) show that the BTE-RAG curves remain above or coincide with the prompt-only baseline throughout the distribution even when 90 % of context is withheld. Histograms (Supplementary Figure S7, S8) reinforce this observation: the ≥ 0.90 similarity bin consistently retains ≥ 40 hits for both models across all pruning levels, demonstrating that a concise subset of top-ranked evidence lines is sufficient to drive the bulk of the performance gains.

3.4 Drug–Biological Process Reasoning

We next asked 842 DrugMechDB questions of the form “Which drug can be used in the treatment of Disease Y by targeting Biological Process P?”. Answer fidelity was again scored with BioBERT-STSB cosine similarity [34].

In rank-ordered plots (Figure 5A), the prompt-only (dashed) and BTE-RAG (solid) curves for both gpt-4o-mini (orange) and gpt-4o (blue) are nearly super-imposable through the first ≈ 600 ranked queries (cosine < 0.70). Beyond this inflection point, the BTE-augmented traces bend upward more steeply, yielding a clear margin in the high-fidelity zone (cosine ≥ 0.80). Thus, retrieval does not alter overall parity but selectively boosts the most mechanistically demanding subset of questions.

Prompt-only histograms (Figure 5B; Supplementary Figure S10, gpt-4o-mini) peak in the 0.20–0.35 range, with ~15 % of answers falling in the ≥ 0.90 bin. Appending the full BTE context nudges the entire distribution rightward (Figure 5C; Supplementary Figure S11-top-left panels). The ≥ 0.90 bin increases by ≈ 5–10 % for both model sizes. These shifts, though smaller than those seen for gene- and metabolite tasks, account for the late-stage separation observed in Figure 5A.

Unlike the previous tasks, performance here depends on retaining a broad evidentiary window. When the lowest-ranked 10–20 % of context lines are removed, the uplift in the ≥ 0.90 bin attenuates, and the rank-ordered curves progressively converge toward the baseline (Supplementary Figure S11-S13). Deeper cuts (> 40 %) essentially erase the retrieval advantage. This suggests that pathway-level questions draw on a more diffuse set of graph triples than gene or metabolite queries, and aggressive trimming can discard critical relational clues. For drug → biological-process reasoning, BTE-RAG

delivers targeted gains in the top decile of similarity scores, provided the complete knowledge-graph context is supplied.

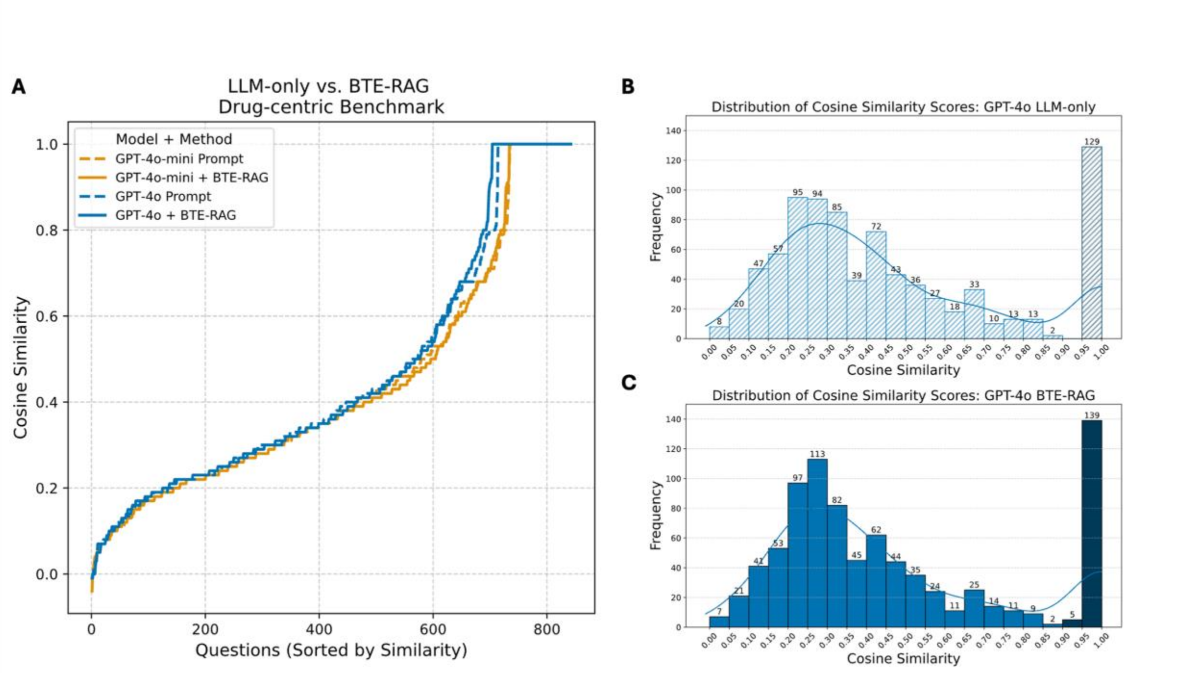

Figure 5: Retrieval-augmented generation maintains overall parity yet excels in the high-fidelity regime of drug-centric mechanistic answers.
(A) Cosine-similarity scores (sentence-transformer embeddings; see *Methods*) between each generated answer and the reference drug→biological-process pathway are plotted for all 842 questions in the Drug Benchmark, ordered from lowest to highest similarity. Dashed traces (LLM-only) and solid traces (BTE-RAG) follow nearly overlapping trajectories across most of the distribution, indicating broadly comparable performance between the two inference modes. However, above a cosine similarity threshold of ≈ 0.7, both *gpt-4o-mini* (orange) and *gpt-4o* (blue) curves generated with BTE context surge ahead of their prompt-only counterparts, revealing a marked advantage in producing highly concordant mechanistic explanations.
(B) Score distribution GPT-4o, LLM-only. Histogram of cosine-similarity scores for GPT-4o answers generated without external context. The hatched bar at 0.90–1.00 marks the high-fidelity zone, capturing 129 near-perfect matches produced by the baseline model.
(C) Score distribution GPT-4o + BTE-RAG. Same format as panel B but for GPT-4o answers produced with BTE-RAG's context. The distribution is right-shifted, and the solid bar in the 0.90–1.00 high-fidelity zone now contains 144 answers, highlighting the enrichment of top-tier mechanistic concordance achieved through retrieval-augmented generation.

To assess sensitivity to similarity-threshold selection, we evaluated performance at cutoffs of 0.85, 0.90, and 0.95 (Supplementary Table S5). For GPT-4o-mini, BTE-RAG produced minimal changes across thresholds, with accuracy increasing from 13.3% to 13.8% at 0.85, from 13.1% to 13.7% at 0.90, and showing no change at 0.95. GPT-4o exhibited modest improvements, with accuracy increasing from 15.6% to 17.3% at 0.85, from 15.3% to 17.1% at 0.90, and from 15.3% to 16.5% at 0.95. The modest and inconsistent gains across thresholds underscore the inherent difficulty of this task,

where linking drugs to biological processes requires more abstract mechanistic reasoning than gene- or metabolite-level queries.

Paired per-item similarity analysis showed no systematic advantage of BTE-RAG over the baseline model for drug-to-biological-process reasoning. Performance differences were small and not statistically distinguishable under paired nonparametric testing, with effect sizes indicating negligible practical impact.

These findings suggest that drug-to-biological-process reasoning represents a more challenging inference regime in which retrieval augmentation provides limited overall benefit.

We conducted a detailed error analysis of all instances in which BTE-RAG underperformed the LLM-only baseline across the three benchmarks for the GPT-4o-mini model. As summarized in Supplementary Table S6, most degradations arose from context dilution (relevant mechanistic entities retrieved but obscured by numerous less-specific entities) or evidence coverage gaps (missing or incomplete mechanistic edges in the federated knowledge graph), with only some contributions from filtering errors (relevant evidence pruned during similarity-based selection) or derivative mismatches (e.g., chloramphenicol palmitate returned instead of chloramphenicol).

These findings reinforce that optimal evidence granularity is task-dependent: concise, high-relevance snippets suffice for gene- and metabolite-level inference, whereas pathway-level queries benefit from a richer contextual fabric. The variation in retrieval effectiveness across tasks, from substantial improvements in gene-level binary classification to modest gains in drug-to-biological-process similarity, highlights that different query types pose distinct mechanistic reasoning challenges. By grounding LLM outputs within curated, biologically meaningful pathways, BTE–RAG consistently accelerates accurate inference, reduces residual errors, and demonstrates considerable promise for advancing automated biomedical hypothesis generation and therapeutic repurposing workflows.

4 Discussion

The rapid advancement of large language models (LLMs) has profoundly reshaped biomedical natural language processing [47]. Despite these advances, current LLMs predominantly operate as opaque systems with implicit knowledge representation, rendering their factual accuracy challenging to verify and limiting their applicability in high-stakes biomedical environments. Recent efforts, such as the knowledge-graph augmented retrieval approach [21], have successfully enhanced biomedical reasoning by integrating disease-specific embeddings from specialized knowledge graphs such as SPOKE [48]. We developed BTE–RAG, a novel retrieval-augmented generation pipeline that strategically incorporates explicit mechanistic evidence from BTE [27]. By leveraging the extensive and federated biomedical knowledge graph of BTE, our method substantially broadens the applicability of knowledge-graph augmented strategies to address diverse query types, including those involving genes, proteins, metabolites, biological processes, diseases and chemical substances. This capability allows BTE–RAG to support complex, multi-domain biomedical inquiries, significantly extending beyond disease-centric queries alone. Our comparative analysis, utilizing a direct "LLM-

only" approach versus the BTE-augmented strategy (Figure 1A) across three rigorously constructed DrugMechDB benchmarks (Figure 1B), demonstrates that incorporating explicit, structured context significantly elevates answer accuracy, enhances transparency, and allows smaller, more computationally efficient models to perform competitively with leading-edge systems. The granularity, explicit mechanistic grounding, and high-quality source attribution inherent in these benchmarks uniquely position them for probing the causal inference capabilities of language models. Comparable mechanistically focused datasets remain scarce in the biomedical domain, as existing resources like PubMedQA or Natural Questions predominantly target document-level retrieval or summarization rather than deep mechanistic inference [49,50].

Traditional LLMs accumulate domain-specific knowledge implicitly during pre-training by statistically modeling large collections of biomedical texts. Although this method yields linguistically coherent responses, it inherently exposes models to the risk of hallucinations, particularly in scenarios involving sparse biomedical facts or multi-step mechanistic reasoning. By contrast, retrieval-augmented generation explicitly anchors model predictions in verifiable external sources, constraining generation to well-substantiated evidence. BTE–RAG advances this paradigm by dynamically federating 61 authoritative biomedical APIs into a single cohesive meta-graph, thereby enabling real-time inclusion of newly curated knowledge in generated responses and ensuring reproducible benchmarking through cached retrievals.

Four critical design principles underpin the efficacy of the BTE–RAG framework. First, the framework leverages an API-centric federation layer that integrates trusted biomedical data sources, including MyGene.info, Gene Ontology, CTDbase, Pubmed central, CHEBI, disease-ontology, DrugBank and more, through unified interface of BTE [29,40,51–53]. Second, it employs semantic query templates aligned with the Translator Reasoner API (TRAPI) standard, selectively retrieving only the most relevant relationships for each question, thereby avoiding extraneous contextual noise. Third, retrieved knowledge graph triples are translated into succinct, directionally explicit declarative statements, seamlessly integrating structured knowledge with natural-language prompts. Fourth, BTE–RAG incorporates flexible context-selection strategies; full-context utilization and cosine similarity-based pruning for scenarios requiring concise, highly relevant context subsets.

Across diverse mechanistic tasks, including gene-centric, metabolite-centric, and drug-centric benchmarks derived from DrugMechDB [31]. BTE-augmented prompting consistently outperformed the LLM-only approach. Notably, the smaller GPT-4o-mini model achieved over sixty-percentage improvement in accuracy on the gene-centric task and eighty-two percent improvement on the metabolite task, when provided with structured BTE evidence. Even GPT-4o, the larger flagship model, demonstrated substantial accuracy gains, underscoring that high-quality, explicit mechanistic context can effectively mitigate the need for extremely large model sizes, suggesting a cost-efficient pathway toward domain-specific accuracy.

While BTE offers comprehensive coverage across numerous biomedical domains, certain areas such as single-cell data, epigenomic profiles, and microbiome interactions remain sparsely represented. Furthermore, variations in curation quality across

federated APIs could inadvertently propagate erroneous edges into model-generated contexts. Although our evaluation leveraged the meticulously curated, high-confidence knowledge graph of DrugMechDB, real-world applications may require strategies for managing lower-confidence or conflicting evidence. A limitation of our current evaluation is that the benchmarks define a single ground-truth mechanistic entity per question, whereas real biological mechanisms often involve multiple interacting components; extending BTE-RAG to multi-entity mechanistic reasoning represents an important direction for future work. Our study employed deterministic prompting to maintain comparability; exploring guided, chain-of-thought prompting strategies could further enhance complex reasoning capabilities but may simultaneously reintroduce hallucinatory risks.

Future developments of BTE–RAG may involve integration into autonomous agent systems capable of iterative querying, generation, self-critiquing, and re-querying, thus facilitating automated self-verification workflows. Expanding the underlying knowledge graph to incorporate resources such as LINCS transcriptomic signatures, tissue-specific interaction networks, and multi-omics datasets would further enrich the mechanistic coverage and broaden applicability [54]. Expanding benchmarking efforts beyond DrugMechDB to encompass open-world biomedical queries could rigorously evaluate and strengthen the capacity BTE–RAG for reliable, contextually grounded inference. Furthermore, adopting frameworks like the Model Context Protocol could harmonize comparisons across diverse generative models, facilitate rigorous auditing, and support real-time decision-making in clinical and regulatory contexts.

In conclusion, BTE–RAG demonstrates the substantial value derived from strategically integrating explicit mechanistic evidence into biomedical language modeling workflows. By significantly improving answer accuracy, interpretability, and computational efficiency, this approach provides a scalable, transparent, and robust foundation for future biomedical AI systems, effectively balancing accuracy, affordability, and trustworthiness.

Supplementary Files:

Supplementary Figure S1: Detailed pipeline for BTE-RAG

Supplementary Figure S2: Contingency table analysis of BTE-RAG performance gains on the GeneTuring benchmark.

Supplementary Figure S3: Contingency table analysis of BTE-RAG performance gains on the Mechanistic Gene benchmark.

Supplementary Figure S4: Performance of BTE-RAG versus an LLM-only baseline on the gene-centric benchmark using gpt-4o-mini.

Supplementary Figure S5: Performance of BTE-RAG versus an LLM-only baseline on the gene-centric benchmark using gpt-4o.

Supplementary Figure S6: Cosine-similarity profile for the metabolite-centric benchmark using GPT-4o-mini in LLM-only mode.

Supplementary Figure S7: Distribution of answer similarities for the metabolite-centric benchmark using GPT-4o-mini in BTE-RAG mode.

Supplementary Figure S8: Distribution of answer similarities for the metabolite-centric benchmark using GPT-4o in BTE-RAG mode.

Supplementary Figure S9: Rank-ordered cosine similarities between model predictions and ground-truth answers on the metabolite-centric benchmark, across context filtering thresholds.

Supplementary Figure S10: Cosine-similarity profile for the drug-centric benchmark using GPT-4o-mini in LLM-only mode.

Supplementary Figure S11: Distribution of answer similarities for the drug-centric benchmark using GPT-4o-mini in BTE-RAG mode.

Supplementary Figure S12: Distribution of answer similarities for the drug-centric benchmark using GPT-4o in BTE-RAG mode.

Supplementary Figure S13: Rank-ordered cosine similarities between model predictions and ground-truth answers on the drug-centric benchmark, across context filtering thresholds.

Supplementary Table S1: Zero-Shot Entity Recognition Performance

Supplementary Table S2: Statistical comparison of BTE-RAG with baseline methods on the GeneTuring gene–disease association benchmark.

Supplementary Table S3: Statistical comparison of BTE-RAG with baseline methods on the Mechanistic Gene benchmark.

Supplementary Table S4: Sensitivity analysis of similarity thresholds for high-fidelity drug–metabolite predictions.

Supplementary Table S5: Sensitivity analysis of similarity thresholds for high-fidelity drug–biological process-disease predictions.

Supplementary Table S6: Analysis of Degradation Cases Across Gene, Metabolite, and Drug–Biological Process Benchmarks

Supplementary Table S7: System Prompts

Availability of Source Code and Requirements

Project name: BTE-RAG

Project home page: https://github.com/janjoy/BTE-RAG

Operating system(s): Linux (Ubuntu)

Programming language: Python 3.10.9

Other requirements: OpenAI Python SDK (openai==1.61.0); additional dependencies listed in requirements file in the repository.

License: Apache-2.0 license

RRID: SCR_027297

Data Availability

All BTE retrieval caches, LLM outputs, and evaluation metrics used in this study are archived in our public GitHub repository [55]. Cached files include complete API responses with query identifiers, entity identifiers, predicates, source names, and all intermediate analysis results.

Author contributions

J.J. and A.S. conceived the project and proposed the benchmark curation. J.J. implemented the code, created the benchmarks and wrote the manuscript. A.S. supervised the study. All authors read and approved of the final manuscript.

Acknowledgements

We thank Willow Callaghan, Mikhael Astorga, and Karthik Soman for insightful discussions, and Everaldo Rodolpho for technical support with high-performance computing resources and server infrastructure.

Competing Interests

The authors declare no competing interests.

Funding

Support for this work was provided by the National Institute on Aging (award R01AG066750), and by the National Center for Advancing Translational Sciences through the Biomedical Data Translator program (awards 1OT2TR003427 and 1OT2TR005710) and the Scripps Research Translational Institute (UM1TR004407). Any opinion expressed in this document does not necessarily reflect the views of NIA, NCATS, SRTI, NIH, individual Translator team members, or affiliated organizations and institutions.

References

1. Hou, W. & Ji, Z. Assessing GPT-4 for cell type annotation in single-cell RNA-seq analysis. *Nat. Methods* **21**, 1462–1465 (2024).

2. Rives, A. *et al.* Biological structure and function emerge from scaling unsupervised learning to 250 million protein sequences. *Proc. Natl. Acad. Sci.* **118**, e2016239118 (2021).
3. Lin, Z. *et al.* Evolutionary-scale prediction of atomic-level protein structure with a language model. *Science* https://doi.org/10.1126/science.ade2574 (2023) doi:10.1126/science.ade2574.
4. Meier, J. *et al.* Language models enable zero-shot prediction of the effects of mutations on protein function. 2021.07.09.450648 Preprint at https://doi.org/10.1101/2021.07.09.450648 (2021).
5. Zheng, Y. *et al.* Large Language Models in Drug Discovery and Development: From Disease Mechanisms to Clinical Trials. Preprint at https://doi.org/10.48550/arXiv.2409.04481 (2024).
6. Miller, K. *et al.* Dynamic few-shot prompting for clinical note section classification using lightweight, open-source large language models. *J. Am. Med. Inform. Assoc.* **32**, 1164–1173 (2025).
7. Ji, Z. *et al.* Survey of Hallucination in Natural Language Generation. *ACM Comput. Surv.* **55**, 1–38 (2023).
8. Vaswani, A. *et al.* Attention Is All You Need. Preprint at https://doi.org/10.48550/arXiv.1706.03762 (2023).
9. Kim, Y. *et al.* Medical Hallucinations in Foundation Models and Their Impact on Healthcare. Preprint at https://doi.org/10.48550/arXiv.2503.05777 (2025).
10. Idnay, B. *et al.* Environment scan of generative AI infrastructure for clinical and translational science. *Npj Health Syst.* **2**, 4 (2025).

11. Ibrahim, M. *et al.* Generative AI for synthetic data across multiple medical modalities: A systematic review of recent developments and challenges. *Comput. Biol. Med.* **189**, 109834 (2025).

12. Maynez, J., Narayan, S., Bohnet, B. & McDonald, R. On Faithfulness and Factuality in Abstractive Summarization. Preprint at https://doi.org/10.48550/arXiv.2005.00661 (2020).

13. Yang, Y., Jin, Q., Huang, F. & Lu, Z. Adversarial Attacks on Large Language Models in Medicine. Preprint at https://doi.org/10.48550/arXiv.2406.12259 (2024).

14. Luo, R. *et al.* BioGPT: generative pre-trained transformer for biomedical text generation and mining. *Brief. Bioinform.* **23**, bbac409 (2022).

15. Kojima, T., Gu, S. S., Reid, M., Matsuo, Y. & Iwasawa, Y. Large Language Models are Zero-Shot Reasoners. Preprint at https://doi.org/10.48550/arXiv.2205.11916 (2023).

16. Brown, T. B. *et al.* Language Models are Few-Shot Learners. Preprint at https://doi.org/10.48550/arXiv.2005.14165 (2020).

17. Lewis, P. *et al.* Retrieval-Augmented Generation for Knowledge-Intensive NLP Tasks. Preprint at https://doi.org/10.48550/arXiv.2005.11401 (2021).

18. Izacard, G. & Grave, E. Leveraging Passage Retrieval with Generative Models for Open Domain Question Answering. Preprint at https://doi.org/10.48550/arXiv.2007.01282 (2021).

19. Karpukhin, V. *et al.* Dense Passage Retrieval for Open-Domain Question Answering. Preprint at https://doi.org/10.48550/arXiv.2004.04906 (2020).

20. Zhang, G. *et al.* Leveraging long context in retrieval augmented language models for medical question answering. *Npj Digit. Med.* **8**, 239 (2025).
21. Soman, K. *et al.* Biomedical knowledge graph-optimized prompt generation for large language models. *Bioinformatics* **40**, btae560 (2024).
22. Hou, W. & Ji, Z. GeneTuring tests GPT models in genomics. 2023.03.11.532238 Preprint at https://doi.org/10.1101/2023.03.11.532238 (2023).
23. Bizon, C. *et al.* ROBOKOP KG and KGB: Integrated Knowledge Graphs from Federated Sources. *J. Chem. Inf. Model.* **59**, 4968–4973 (2019).
24. Mungall, C. J. *et al.* The Monarch Initiative: an integrative data and analytic platform connecting phenotypes to genotypes across species. *Nucleic Acids Res.* **45**, D712–D722 (2017).
25. Pan, S. *et al.* Unifying Large Language Models and Knowledge Graphs: A Roadmap. *IEEE Trans. Knowl. Data Eng.* **36**, 3580–3599 (2024).
26. Evangelista, J. E. *et al.* Toxicology knowledge graph for structural birth defects. *Commun. Med.* **3**, 98 (2023).
27. Callaghan, J. *et al.* BioThings Explorer: a query engine for a federated knowledge graph of biomedical APIs. *Bioinformatics* **39**, btad570 (2023).
28. Gene Ontology Consortium. The Gene Ontology resource: enriching a GOld mine. *Nucleic Acids Res.* **49**, D325–D334 (2021).
29. Knox, C. *et al.* DrugBank 6.0: the DrugBank Knowledgebase for 2024. *Nucleic Acids Res.* **52**, D1265–D1275 (2024).
30. Fecho, K. *et al.* Progress toward a universal biomedical data translator. *Clin. Transl. Sci.* **15**, 1838–1847 (2022).

31. Gonzalez-Cavazos, A. C. *et al.* DrugMechDB: A Curated Database of Drug Mechanisms. *Sci. Data* **10**, 632 (2023).

32. GPT-4o System Card. https://openai.com/index/gpt-4o-system-card/.

33. Deka, P., Jurek-Loughrey, A. & P., D. Improved Methods to Aid Unsupervised Evidence-Based Fact Checking for Online Heath News. *J. Data Intell.* **3**, 474–504 (2022).

34. Deka, P., Jurek-Loughrey, A. & P, D. Evidence Extraction to Validate Medical Claims in Fake News Detection. in *Health Information Science* (eds Traina, A. et al.) vol. 13705 3–15 (Springer Nature Switzerland, Cham, 2022).

35. pritamdeka/S-PubMedBert-MS-MARCO · Hugging Face. https://huggingface.co/pritamdeka/S-PubMedBert-MS-MARCO.

36. pritamdeka/BioBERT-mnli-snli-scinli-scitail-mednli-stsb · Hugging Face. https://huggingface.co/pritamdeka/BioBERT-mnli-snli-scinli-scitail-mednli-stsb.

37. Jin, Q., Yang, Y., Chen, Q. & Lu, Z. GeneGPT: augmenting large language models with domain tools for improved access to biomedical information. *Bioinformatics* **40**, btae075 (2024).

38. Shang, X., Liao, X., Ji, Z. & Hou, W. Benchmarking large language models for genomic knowledge with GeneTuring. 2023.03.11.532238 Preprint at https://doi.org/10.1101/2023.03.11.532238 (2025).

39. Unni, D. R. *et al.* Biolink Model: A universal schema for knowledge graphs in clinical, biomedical, and translational science. *Clin. Transl. Sci.* **15**, 1848–1855 (2022).

40. Wu, C., Macleod, I. & Su, A. I. BioGPS and MyGene.info: organizing online, gene-centric information. *Nucleic Acids Res.* **41**, D561-565 (2013).

41. OpenAI *et al.* GPT-4 Technical Report. Preprint at https://doi.org/10.48550/arXiv.2303.08774 (2024).

42. GPT-4o mini Model | OpenAI API. https://platform.openai.com.

43. GPT-4o Model | OpenAI API. https://platform.openai.com.

44. Chen, Y., Gao, J., Petruc, M., Popescu, M. & Xu, D. Iterative Prompt Refinement for Mining Gene Relationships from ChatGPT.

45. Marvin, G., Hellen Raudha, N., Jjingo, D. & Nakatumba-Nabende, J. Prompt Engineering in Large Language Models. in 387–402 (2024). doi:10.1007/978-981-99-7962-2_30.

46. Sahoo, P. *et al.* A Systematic Survey of Prompt Engineering in Large Language Models: Techniques and Applications. Preprint at https://doi.org/10.48550/arXiv.2402.07927 (2025).

47. Li, M., Kilicoglu, H., Xu, H. & Zhang, R. BiomedRAG: A retrieval augmented large language model for biomedicine. *J. Biomed. Inform.* **162**, 104769 (2025).

48. Morris, J. H. *et al.* The scalable precision medicine open knowledge engine (SPOKE): a massive knowledge graph of biomedical information. *Bioinforma. Oxf. Engl.* **39**, btad080 (2023).

49. Jin, Q., Dhingra, B., Liu, Z., Cohen, W. W. & Lu, X. PubMedQA: A Dataset for Biomedical Research Question Answering. Preprint at https://doi.org/10.48550/arXiv.1909.06146 (2019).

50. Kwiatkowski, T. *et al.* Natural Questions: A Benchmark for Question Answering Research. *Trans. Assoc. Comput. Linguist.* **7**, 452–466 (2019).

51. Davis, A. P. *et al.* Comparative Toxicogenomics Database (CTD): update 2023. *Nucleic Acids Res.* **51**, D1257–D1262 (2023).

52. Schriml, L. M. *et al.* Disease Ontology: a backbone for disease semantic integration. *Nucleic Acids Res.* **40**, D940-946 (2012).

53. UniProt Consortium. UniProt: the Universal Protein Knowledgebase in 2025. *Nucleic Acids Res.* **53**, D609–D617 (2025).

54. Pilarczyk, M. *et al.* Connecting omics signatures and revealing biological mechanisms with iLINCS. *Nat. Commun.* **13**, 4678 (2022).

55. BTE-RAG/data/analysis_results at main · janjoy/BTE-RAG. *GitHub* https://github.com/janjoy/BTE-RAG/tree/main/data/analysis_results.

Click here to access/download
Supplementary Material
Joy_et_al_Supplementary_info_v2.pdf

Thank you for the thoughtful comments and suggestions. We have addressed all points and incorporated the corresponding revisions into the manuscript, with changes highlighted in blue in the relevant sections.

Reviewer's Comments

Reviewer #1

Thank you for the opportunity to review "Federated Knowledge Retrieval Elevates Large Language Model Performance on Biomedical Benchmarks." The paper tackles a timely and important problem: grounding large language models in mechanistic evidence to reduce unsupported claims. It does so with a thoughtful design that layers BTE-RAG over a federation of approximately 60 biomedical APIs and evaluates three complementary DrugMechDB-derived benchmarks (gene, metabolite, drug to process). The manuscript is clearly written, the technical contribution is meaningful, and the experimental results are promising.

Recommendation: Major revision.

Below are concrete, actionable changes that would bring the work in line with GigaScience's standards for FAIR availability, licensing, documentation, testing, and reproducibility. Many are straightforward, but together they matter for long-term reuse and auditability.

1) Statistical rigor: paired inference, uncertainty, variance

The manuscript reports compelling descriptive gains. Because each benchmark item is answered under both conditions (LLM-only and BTE-RAG), the study is a paired design. In paired settings, descriptive plots and point estimates are not sufficient to establish that improvements exceed sampling noise or threshold tuning. Please add paired statistical evidence that quantifies: (i) whether the gains are reliable, (ii) how large they are in practical terms, and (iii) how stable they are under repeated runs or under a fully deterministic pipeline.

Gene task (binary): Report McNemar's test on the existing 2×2 tables, along with 95 percent Wilson confidence intervals for each condition and a Newcombe confidence interval for the accuracy difference. Keep the flip counts in the text.

Metabolite and drug-to-process tasks (similarity): Report paired bootstrap confidence intervals or Wilcoxon signed-rank tests on per-item similarity differences (BTE-RAG minus baseline). Include a nonparametric effect size such as Cliff's delta with its confidence interval.

Threshold validation: Treat the greater-than-or-equal-to 0.90 "high-fidelity" threshold as a choice that should be validated. Show sensitivity across nearby cutoffs such as 0.85, 0.90, and 0.95, and add a small blinded expert adjudication (about 50 to 100 items) to confirm that the high-cosine band corresponds to acceptable correctness.

Variance or determinism: Either document end-to-end determinism (frozen retrieval caches, fixed ordering, pinned embeddings) or run at least three replicates and report mean and standard deviation.

We thank the reviewer for this helpful comment on statistical rigor and have substantially revised the analysis to incorporate paired inference, uncertainty quantification, and robustness checks, with new results added to the Results section. For the gene task, these analyses demonstrate statistically significant and practically meaningful improvements for all models (updated results in Section 3.2, Figure 3, Supplementary Table S3).

The metabolite task showed statistically significant improvements for both models (small effect for GPT-4o-mini; negligible for GPT-4o), whereas the drug–biological process task showed no significant improvements and negligible effects. Threshold sensitivity analyses yielded consistent conclusions across both tasks, supporting robustness to threshold choice (Sections 3.3–3.4; Supplementary Tables S4–S5). We acknowledge that blinded expert analysis would strengthen validation of the similarity-based thresholds but extend beyond the current revision scope and is a good suggestion for future analysis. Our pipeline is fully deterministic and reproducible (temperature = 0, cached retrievals, fixed embedding models, and saved intermediate outputs), ensuring all reported results are exactly reproducible. The evaluation can be reproduced by re-running analysis on these cached outputs without re-querying APIs or re-generating text. This deterministic design ensures that our reported improvements are not subject to stochastic variation and would be reproduced in independent verification.

2) Benchmark scope and generalizability

All three evaluations are derived from DrugMechDB, which makes the study internally consistent but also couples the tasks to a single curation philosophy and evidence distribution. Please acknowledge this limitation explicitly in the Discussion and, ideally, add an external validation on at least one independent source to demonstrate generalizability. Options include CTD (drug-gene-process links), Reactome or GO (pathway and process grounding), DisGeNET (gene-disease associations), or a lightweight question answering set sourced outside DrugMechDB. Even a modest external set of about 100 to 200 items, evaluated with the same paired protocols and identifier-based scoring, would strengthen the claim. If full external validation is not

feasible for this revision, please include robustness checks such as a date-based split, entity-family holdouts, and per-source ablations.

Thank you for this suggestion, we have now added an additional evaluation using the GeneTuring gene–disease association benchmark and analyzed four approaches on this dataset: LLM-only prompting, BTE-RAG, GeneGPT-Full, and GeneGPT-Slim. These results are reported in Section 3.1 and Figure 2 and demonstrate that the relative performance trends observed on DMDB generalize to an independent dataset.

We introduced the DrugMechDB (DMDB)–derived benchmarks to specifically probe mechanistic and nontrivial relationships between drug–disease pairs, where LLM-only prompting is often insufficient because it primarily captures surface-level associations rather than explicit mechanistic reasoning.

3) Licensing, attribution, and persistent identifiers

The project is MIT-licensed and adapts components from BaranziniLab/KG_RAG (Apache-2.0) and SuLab/DrugMechDB (CC0-1.0). To meet license obligations and align with FAIR and the Joint Declaration of Data Citation Principles, please:

(i) keep Apache-licensed code under Apache with the upstream LICENSE and NOTICE files, noting any modifications;

(ii) include the CC0 dedication text for any DrugMechDB artifacts and note that CC0 provides no patent grant;

(iii) archive with DOIs (GigaDB preferred?) the three benchmarks, the exact evaluation caches used in the paper, and a tagged software release of the repository;

(iv) license datasets under CC0 or CC BY while keeping the code MIT;

(v) add a short Data and Software Availability table listing artifact, DOI or URL, license, and version or date.

Thank you for these recommendations on licensing compliance and best practices. We have relicensed all source code to Apache 2.0 (from MIT) to match upstream KG_RAG components, added a file to repo documenting modifications, and included CC0 1.0 dedication text for DrugMechDB benchmarks with explicit notation that CC0 provides no patent grant. After peer-review acceptance, we will archive a tagged software release and all benchmark datasets in the Software Heritage Archive to obtain persistent DOIs. We will then update the repository and manuscript with these identifiers as the version of record.

4) Error analysis and degradation cases

Please add a brief failure analysis focused on where BTE-RAG reduces accuracy relative to LLM-only. At minimum, report the total number and percent of right-to-wrong flips per task and include a small set of representative cases. For each example, show the input, expected and predicted outputs, the top retrieved evidence with identifiers and timestamps, and a one-line diagnosis of the likely cause (for example normalization

mismatch, retrieval coverage gap, ranking or filtering that hid relevant context, or long-context truncation). A short summary that groups the main causes into two or three buckets will make the results more interpretable and point to practical fixes.

We thank the reviewer for this important suggestion and have performed a detailed analysis of all degradation cases across the gene, metabolite, and drug–biological process benchmarks (for the GPT-4o-mini model). We categorized each case by underlying error mode and quantified the contributions of context dilution, evidence coverage gaps, filtering errors, and derivative mismatches. As summarized in the revised Results section (Supplementary Table S6) and detailed in our repository at https://github.com/janjoy/BTE-RAG/tree/main/error_analysis, most failures arose from context dilution (relevant mechanistic entities were retrieved but obscured by numerous less-specific entities), evidence coverage gaps (missing mechanistic edges in the federated knowledge graph), filtering errors (relevant evidence pruned during similarity-based selection), or derivative mismatch (e.g., predicting chloramphenicol palmitate instead of chloramphenicol).

5) Methodological transparency: embedding and scoring models

Please add two or three sentences in Methods explaining why S-PubMedBERT-MS-MARCO is used for filtering retrieved context while a BioBERT-based model is used for semantic similarity scoring, and what advantages each provides over plausible alternatives. A brief rationale will strengthen methodological transparency.

Thank you for this suggestion to improve methodological transparency. We have added a detailed rationale explaining our choice of S-PubMedBERT-MS-MARCO for retrieval filtering and the BioBERT-based model for semantic similarity scoring, which is now described in Section 2.1 (“Embedding and Scoring Models”). We agree that this clarification strengthens the manuscript.

6) Reproducibility workflow and archived caches

Because BTE federates live APIs, results can drift as sources update. Please archive the exact retrieval caches used in evaluation with DOIs and minimal provenance if at all possible (query identifier, subject and object identifiers, predicate, source name and version or access date, any confidence score, and a retrieval timestamp).

We thank the reviewer for this important point regarding reproducibility. Given that BTE queries federated APIs whose content may update over time, we ran all evaluations from a frozen set of retrieval caches to ensure reproducibility. These caches are publicly available in our GitHub repository at https://github.com/janjoy/BTE-RAG/tree/main/data/analysis_results. Each cache file contains: (i) the complete API context returned by BioThings Explorer, (ii) the subset of context passed to the LLM, and (iii) all analyzed outputs including similarity scores and accuracy measurements. Metadata includes query identifiers, subject and object identifiers (e.g., drug MeSH IDs, disease IDs, gene symbols), predicates (relationship types) etc. We also provide all

cached LLM responses from both LLM-only and BTE-RAG conditions (temperature=0 for deterministic generation). This archiving ensures reproducibility of our reported results, even if upstream APIs change. We have added a Data Availability statement in the manuscript directing readers to these archived resources.

In summary, this is a promising and well-motivated study that could make a useful contribution once the statistical evidence, FAIR availability, and reproducibility pieces are tightened as outlined above. I recommend Major Revision and am happy to re-review a revised version.

Reviewer #2

This paper introduces BTE-RAG, a system that combines large language models with biomedical knowledge from BioThings Explorer. Tested on three benchmarks built from DrugMechDB (genes, metabolites, and drug-process links), it shows clear accuracy gains compared to using LLMs alone.

Strengths:

The work demonstrates that retrieval improves both small and large models, suggesting cost-efficiency and scalability. This paper also curated multi-scale QA datasets (gene, metabolite, drug) from DrugMechDB provide structured, reproducible evaluation.

Weaknesses:

1. This dual-route design is conceptually sound but too narrow a baseline. A stronger evaluation would compare against other RAG systems (PubMed-based retrieval, BiomedRAG, SPOKE-RAG) instead of just "LLM-only."

We thank the reviewer for this helpful suggestion. Our initial goal was to isolate the contribution of API-driven, structured knowledge graph retrieval by directly comparing BTE-RAG against LLM-only prompting, thereby establishing whether explicit mechanistic retrieval provides advantages beyond prompting alone. However, we have now expanded the evaluation to include GeneGPT (Jin et al, 2024), a representative biomedical RAG framework that integrates external knowledge via NCBI APIs and in-context learning. Specifically, we evaluated both GeneGPT-Full and GeneGPT-Slim alongside BTE-RAG and LLM-only prompting on two benchmarks where GeneGPT is directly applicable: our mechanistic gene benchmark and the GeneTuring gene–disease association dataset (Shang et al, 2025). These additional comparisons, now reported in Sections 3.1 and 3.2, provide a broader RAG baseline and help position BTE-RAG

relative to existing biomedical RAG approaches while preserving a controlled comparison focused on mechanistic reasoning.

2. For Entity Recognition step, using pre-annotated entities in benchmarks artificially simplifies the problem. In real-world biomedical QA, entity recognition itself is a major challenge (e.g., ambiguous drug synonyms, rare disease names). Besides, the zero-shot extraction module is described but not evaluated. The paper should report precision/recall of entity recognition to show feasibility beyond curated inputs.

We thank the reviewer for raising this valuable point and to address this, we evaluated our zero-shot entity extraction module on the full set of benchmark questions. As reported in the new Supplementary Table S1, the model achieves high precision and recall (>0.90 across all entity types) and strong joint extraction accuracy (0.89–0.99). These results indicate that automated entity recognition is feasible for an end-to-end BTE-RAG workflow. We also note in the manuscript (Section 2.1, Entity Recognition) that integrating this module into a fully automated pipeline and evaluating it on unannotated biomedical queries is an important direction for future work.

3. No error analysis of BTE retrieval quality is provided. If BTE returns wrong or noisy triples, how often does this mislead the LLM? Adding experiment to show that would strengthen the study.

We thank the reviewer for highlighting the importance of evaluating retrieval quality and its downstream effects on model performance. We conducted a systematic error analysis of all cases in which BTE-RAG underperformed the LLM-only baseline, thereby directly quantifying how often noisy, incomplete, or misleading retrieved triples negatively influenced the LLM. This analysis revealed that most degradation cases were attributable to context dilution (noisy or overly broad retrieved evidence) or evidence coverage gaps (missing mechanistic edges), with only some contributions from filtering errors or derivative mismatches. These results are now presented in the revised Results section (Supplementary Table S6), with full case-level breakdowns provided in our repository at https://github.com/janjoy/BTE-RAG/tree/main/error_analysis.

4. Though the authors used SOTA LLMs, however, the choice of only OpenAI GPT-4o family is narrow. No comparison with open-source biomedical LLMs (e.g., BioGPT, Meditron, PubMedBERT-RAG). Comparison with these model would increase the generalizability

We appreciate the reviewer’s suggestion to broaden model comparisons and agree that diversity is important for generalizability. Our model selection was guided by two goals: (i) benchmarking BTE-RAG against strong baselines for biomedical question answering,

and (ii) ensuring fair comparison with systems explicitly designed for mechanistic reasoning and structured retrieval.

In addition to GPT-4o and GPT-4o-mini, we therefore included GeneGPT (Full and Slim) RAG models, which is specifically designed for biomedical reasoning via structured retrieval from NCBI and BLAST resources. GeneGPT was evaluated alongside BTE-RAG on both an external benchmark (GeneTuring gene–disease association) and one of our mechanistic gene benchmarks, enabling assessment across independent datasets.

We did not include additional biomedical LLMs such as BioGPT or PubMedBERT models because prior large-scale evaluations show that these domain-specific models substantially underperform state-of-the-art general-purpose LLMs on complex biomedical reasoning tasks. For example, the GeneTuring benchmark reports near-zero or very low accuracy for several biomedical LLMs, whereas ChatGPT-based models perform substantially better (Shang et al., 2025). Similarly, MultiMedQA evaluations demonstrate that larger general-purpose models such as GPT-4 outperform smaller domain-specific models by wide margins (Singhal et al., 2025).

Consistent with this literature, we selected GPT-4o family models as strong, widely used baselines and focused our evaluation on whether explicit mechanistic retrieval improves reasoning even from competitive starting points. We agree that extending BTE-RAG to additional open-source biomedical models is an important direction for future work.

5. Reliance on one source (DrugMechDB) makes evaluation narrow. The authors should demonstrate performance on at least one independent dataset (e.g., BioASQ, PubMedQA, SPOKE-based tasks) to show broader utility.

We thank the reviewer for this thoughtful comment, and we have now expanded our evaluation to include an independent benchmark, the GeneTuring gene–disease association dataset (which is distinct from DrugMechDB) and is used to assess large language models on genomics knowledge. On this benchmark, we compared LLM-only prompting, BTE-RAG, and two additional biomedical RAG approaches (GeneGPT-Full and GeneGPT-Slim). These results are now reported in Section 3.1 and Figure 2. Together, the inclusion of the GeneTuring benchmark and the DrugMechDB-derived mechanistic tasks demonstrates that BTE-RAG is effective both on an independent association-based dataset and on more complex and mechanistic reasoning problems.

6. Cosine similarity ≥0.9 is arbitrary; should provide ROC/AUC or threshold sensitivity.

We thank the reviewer for this suggestion. To address the concern about threshold selection, we have performed comprehensive threshold sensitivity analyses across 0.85, 0.90, and 0.95 cutoffs for both the metabolite and drug-to-biological-process benchmarks. These analyses, now included in the Results section (sections 3.3 and

3.4), demonstrate that BTE-RAG's relative improvements over LLM-only baselines remain consistent across all examined thresholds. For the metabolite task, we observed robust gains with effect sizes (Cliff's δ) ranging from 0.109 to 0.144 across thresholds. For the drug-to-biological-process task, minimal improvements (Cliff's δ: 0.000–0.018) were consistent across all cutoffs. We also report Wilcoxon signed-rank tests on per-item similarity differences to establish statistical significance.

7. Benchmarks enforce exactly one correct gene, metabolite, or drug per question. Real mechanisms often involve multiple parallel or interacting entities. The single-answer design hides biological complexity and creates an artificial task.

We appreciate this thoughtful comment and fully agree that biological mechanisms often involve multiple parallel or interacting entities. Our choice to use single-answer benchmarks from DrugMechDB was motivated by the need for objective, automated evaluation with clear ground truth that enables rigorous head-to-head comparison between LLM-only and BTE-RAG conditions. While this design necessarily simplifies biological complexity, it provides a reproducible and interpretable baseline for assessing whether knowledge graph context enhances mechanistic reasoning. Importantly, the BTE-RAG framework itself is not limited to single-entity outputs, and the retrieval step already surfaces multiple mechanistic candidates. We have added discussion of this limitation in the Discussion section, noting that real biomedical questions often have multiple valid answers involving pathway and network-based interactions. Future work will extend evaluation to multi-answer or network-based formats that better capture the parallelism and complexity of real biological systems.

8. Ground truth relies on exact HGNC, CHEBI, or DrugBank IDs. Why the ambiguities (synonyms, deprecated IDs, overlapping terms) are filtered out rather than addressed? This may bias the dataset toward easier, cleaner cases.

We appreciate this concern and would like to clarify our design. For the gene benchmark, we used HGNC symbols rather than IDs, as symbols are generally more natural for LLMs and reduce confusion from numerical identifiers. For metabolites and drugs, we did not rely on CHEBI or DrugBank IDs; instead, we evaluated model outputs against names using cosine similarity of BioBERT-STSB text embeddings, a metric that captures graded lexical and semantic overlap rather than strict string matching. This approach inherently accommodates synonyms and overlapping terms while avoiding penalization for minor wording differences. Our primary goal in this work was to establish a fair comparison between LLM-only and BTE-RAG, and therefore we prioritized clarity and consistency in evaluation. In future iterations, we plan to extend the framework with explicit synonym resolution and deprecated mappings to further test robustness under noisier, real-world conditions.

9. The paper cited recent biomedical RAG systems such as BiomedRAG, GeneTuring but didn't compare with them (e.g., BiomedRAG). BioRAG (2024) is also highly relevant. These works are highly relevant baselines, showing retrieval from knowledge graphs, APIs, or literature, and including them in comparison would better position BTE-RAG within the current state of the art and highlight its unique contributions.

Thank you for this suggestion, we have now expanded our empirical evaluation to include GeneGPT, a representative biomedical RAG framework that integrates structured knowledge via NCBI (National Center for Biotechnology Information) APIs and in-context learning (Jin et al., 2024). Specifically, we evaluated both the GeneGPT-Full and GeneGPT-Slim configurations alongside LLM-only prompting and BTE-RAG on the GeneTuring gene–disease association benchmark as well as on our newly introduced mechanistic gene benchmark. These results are now reported in Sections 3.1 and 3.2, with detailed statistical analyses provided in the main text and supplementary materials. We agree that broader multi-model comparisons are valuable, and incorporating additional biomedical RAG systems and related approaches is an important direction for future work.
