# Supplementary material for: Federated knowledge retrieval elevates large language model performance on biomedical benchmarks
Source: Gigascience. 2026 Jan 19;15:giag007. doi: 10.1093/gigascience/giag007 (PMC12888809; doi:10.1093/gigascience/giag007)
Supplement: giag007_Authors_Response_To_Reviewer_Comments_original_submission [file giag007_authors_response_to_reviewer_comments_original_submission.pdf]

Author's Response To Reviewer Comments

The response is also included in the joy_reviewer_response.pdf

----

Thank you for the thoughtful comments and suggestions. We have addressed all points and incorporated the corresponding revisions into the manuscript, with changes highlighted in blue in the relevant sections.
Reviewer’s Comments

Reviewer #1
Thank you for the opportunity to review "Federated Knowledge Retrieval Elevates Large Language Model Performance on Biomedical Benchmarks." The paper tackles a timely and important problem: grounding large language models in mechanistic evidence to reduce unsupported claims. It does so with a thoughtful design that layers BTE-RAG over a federation of approximately 60 biomedical APIs and evaluates three complementary DrugMechDB-derived benchmarks (gene, metabolite, drug to process). The manuscript is clearly written, the technical contribution is meaningful, and the experimental results are promising.

Recommendation: Major revision.

Below are concrete, actionable changes that would bring the work in line with GigaScience's standards for FAIR availability, licensing, documentation, testing, and reproducibility. Many are straightforward, but together they matter for long-term reuse and auditability.

1) Statistical rigor: paired inference, uncertainty, variance
The manuscript reports compelling descriptive gains. Because each benchmark item is answered under both conditions (LLM-only and BTE-RAG), the study is a paired design. In paired settings, descriptive plots and point estimates are not sufficient to establish that improvements exceed sampling noise or threshold tuning. Please add paired statistical evidence that quantifies: (i) whether the gains are reliable, (ii) how large they are in practical terms, and (iii) how stable they are under repeated runs or under a fully deterministic pipeline.

Gene task (binary): Report McNemar's test on the existing 2×2 tables, along with 95 percent Wilson confidence intervals for each condition and a Newcombe confidence interval for the accuracy difference. Keep the flip counts in the text.
Metabolite and drug-to-process tasks (similarity): Report paired bootstrap confidence intervals or Wilcoxon signed-rank tests on per-item similarity differences (BTE-RAG minus baseline). Include a nonparametric effect size such as Cliff's delta with its confidence interval.
Threshold validation: Treat the greater-than-or-equal-to 0.90 "high-fidelity" threshold as a choice that should be validated. Show sensitivity across nearby cutoffs such as 0.85, 0.90, and 0.95, and add a small blinded expert adjudication (about 50 to 100 items) to confirm that the high-cosine band corresponds to acceptable correctness.
Variance or determinism: Either document end-to-end determinism (frozen retrieval caches, fixed ordering, pinned embeddings) or run at least three replicates and report mean and standard deviation.

We thank the reviewer for this helpful comment on statistical rigor and have substantially revised the analysis to incorporate paired inference, uncertainty quantification, and robustness checks, with new results added to the Results section. For the gene task, these analyses demonstrate statistically significant and practically meaningful improvements for all models (updated results in Section 3.2, Figure 3, Supplementary Table S3).
The metabolite task showed statistically significant improvements for both models (small effect for GPT-4o-mini; negligible for GPT-4o), whereas the drug–biological process task showed no significant improvements and negligible effects. Threshold sensitivity analyses yielded consistent conclusions across both tasks, supporting robustness to threshold choice (Sections 3.3–3.4; Supplementary Tables S4–S5). We acknowledge that blinded expert analysis would strengthen validation of the similarity-based thresholds but

extend beyond the current revision scope and is a good suggestion for future analysis. Our pipeline is fully deterministic and reproducible (temperature = 0, cached retrievals, fixed embedding models, and saved intermediate outputs), ensuring all reported results are exactly reproducible. The evaluation can be reproduced by re-running analysis on these cached outputs without re-querying APIs or re-generating text. This deterministic design ensures that our reported improvements are not subject to stochastic variation and would be reproduced in independent verification.

2) Benchmark scope and generalizability
All three evaluations are derived from DrugMechDB, which makes the study internally consistent but also couples the tasks to a single curation philosophy and evidence distribution. Please acknowledge this limitation explicitly in the Discussion and, ideally, add an external validation on at least one independent source to demonstrate generalizability. Options include CTD (drug-gene-process links), Reactome or GO (pathway and process grounding), DisGeNET (gene-disease associations), or a lightweight question answering set sourced outside DrugMechDB. Even a modest external set of about 100 to 200 items, evaluated with the same paired protocols and identifier-based scoring, would strengthen the claim. If full external validation is not feasible for this revision, please include robustness checks such as a date-based split, entity-family holdouts, and per-source ablations.

Thank you for this suggestion, we have now added an additional evaluation using the GeneTuring gene–disease association benchmark and analyzed four approaches on this dataset: LLM-only prompting, BTE-RAG, GeneGPT-Full, and GeneGPT-Slim. These results are reported in Section 3.1 and Figure 2 and demonstrate that the relative performance trends observed on DMDB generalize to an independent dataset.
We introduced the DrugMechDB (DMDB)–derived benchmarks to specifically probe mechanistic and nontrivial relationships between drug–disease pairs, where LLM-only prompting is often insufficient because it primarily captures surface-level associations rather than explicit mechanistic reasoning.

3) Licensing, attribution, and persistent identifiers
The project is MIT-licensed and adapts components from BaranziniLab/KG_RAG (Apache-2.0) and SuLab/DrugMechDB (CC0-1.0). To meet license obligations and align with FAIR and the Joint Declaration of Data Citation Principles, please:
(i) keep Apache-licensed code under Apache with the upstream LICENSE and NOTICE files, noting any modifications;
(ii) include the CC0 dedication text for any DrugMechDB artifacts and note that CC0 provides no patent grant;
(iii) archive with DOIs (GigaDB preferred?) the three benchmarks, the exact evaluation caches used in the paper, and a tagged software release of the repository;
(iv) license datasets under CC0 or CC BY while keeping the code MIT;
(v) add a short Data and Software Availability table listing artifact, DOI or URL, license, and version or date.

Thank you for these recommendations on licensing compliance and best practices. We have relicensed all source code to Apache 2.0 (from MIT) to match upstream KG_RAG components, added a file to repo documenting modifications, and included CC0 1.0 dedication text for DrugMechDB benchmarks with explicit notation that CC0 provides no patent grant. After peer-review acceptance, we will archive a tagged software release and all benchmark datasets in the Software Heritage Archive to obtain persistent DOIs. We will then update the repository and manuscript with these identifiers as the version of record.

4) Error analysis and degradation cases
Please add a brief failure analysis focused on where BTE-RAG reduces accuracy relative to LLM-only. At minimum, report the total number and percent of right-to-wrong flips per task and include a small set of representative cases. For each example, show the input, expected and predicted outputs, the top retrieved evidence with identifiers and timestamps, and a one-line diagnosis of the likely cause (for example normalization mismatch, retrieval coverage gap, ranking or filtering that hid relevant context, or long-context truncation). A short summary that groups the main causes into two or three buckets will make the results more interpretable and point to practical fixes.

We thank the reviewer for this important suggestion and have performed a detailed analysis of all degradation cases across the gene, metabolite, and drug–biological process benchmarks (for the GPT-4o-mini model). We categorized each case by underlying error mode and quantified the contributions of context dilution, evidence coverage gaps, filtering errors, and derivative mismatches. As summarized in the revised Results section (Supplementary Table S6) and detailed in our repository at https://github.com/janjoy/BTE-RAG/tree/main/error_analysis, most failures arose from context dilution (relevant mechanistic entities were retrieved but obscured by numerous less-specific entities), evidence coverage gaps (missing mechanistic edges in the federated knowledge graph), filtering errors (relevant evidence pruned during similarity-based selection), or derivative mismatch (e.g., predicting chloramphenicol palmitate instead of chloramphenicol).

5) Methodological transparency: embedding and scoring models
Please add two or three sentences in Methods explaining why S-PubMedBERT-MS-MARCO is used for filtering retrieved context while a BioBERT-based model is used for semantic similarity scoring, and what advantages each provides over plausible alternatives. A brief rationale will strengthen methodological transparency.

Thank you for this suggestion to improve methodological transparency. We have added a detailed rationale explaining our choice of S-PubMedBERT-MS-MARCO for retrieval filtering and the BioBERT-based model for semantic similarity scoring, which is now described in Section 2.1 ("Embedding and Scoring Models"). We agree that this clarification strengthens the manuscript.

6) Reproducibility workflow and archived caches
Because BTE federates live APIs, results can drift as sources update. Please archive the exact retrieval caches used in evaluation with DOIs and minimal provenance if at all possible (query identifier, subject and object identifiers, predicate, source name and version or access date, any confidence score, and a retrieval timestamp).

We thank the reviewer for this important point regarding reproducibility. Given that BTE queries federated APIs whose content may update over time, we ran all evaluations from a frozen set of retrieval caches to ensure reproducibility. These caches are publicly available in our GitHub repository at https://github.com/janjoy/BTE-RAG/tree/main/data/analysis_results. Each cache file contains: (i) the complete API context returned by BioThings Explorer, (ii) the subset of context passed to the LLM, and (iii) all analyzed outputs including similarity scores and accuracy measurements. Metadata includes query identifiers, subject and object identifiers (e.g., drug MeSH IDs, disease IDs, gene symbols), predicates (relationship types) etc. We also provide all cached LLM responses from both LLM-only and BTE-RAG conditions (temperature=0 for deterministic generation). This archiving ensures reproducibility of our reported results, even if upstream APIs change. We have added a Data Availability statement in the manuscript directing readers to these archived resources.

In summary, this is a promising and well-motivated study that could make a useful contribution once the statistical evidence, FAIR availability, and reproducibility pieces are tightened as outlined above. I recommend Major Revision and am happy to re-review a revised version.

Reviewer #2
This paper introduces BTE-RAG, a system that combines large language models with biomedical knowledge from BioThings Explorer. Tested on three benchmarks built from DrugMechDB (genes, metabolites, and drug-process links), it shows clear accuracy gains compared to using LLMs alone.

Strengths:
The work demonstrates that retrieval improves both small and large models, suggesting cost-efficiency and scalability. This paper also curated multi-scale QA datasets (gene, metabolite, drug) from DrugMechDB provide structured, reproducible evaluation.

Weaknesses:
1. This dual-route design is conceptually sound but too narrow a baseline. A stronger evaluation would

compare against other RAG systems (PubMed-based retrieval, BiomedRAG, SPOKE-RAG) instead of just "LLM-only."

We thank the reviewer for this helpful suggestion. Our initial goal was to isolate the contribution of API-driven, structured knowledge graph retrieval by directly comparing BTE-RAG against LLM-only prompting, thereby establishing whether explicit mechanistic retrieval provides advantages beyond prompting alone. However, we have now expanded the evaluation to include GeneGPT (Jin et al, 2024), a representative biomedical RAG framework that integrates external knowledge via NCBI APIs and in-context learning. Specifically, we evaluated both GeneGPT-Full and GeneGPT-Slim alongside BTE-RAG and LLM-only prompting on two benchmarks where GeneGPT is directly applicable: our mechanistic gene benchmark and the GeneTuring gene–disease association dataset (Shang et al, 2025). These additional comparisons, now reported in Sections 3.1 and 3.2, provide a broader RAG baseline and help position BTE-RAG relative to existing biomedical RAG approaches while preserving a controlled comparison focused on mechanistic reasoning.

2. For Entity Recognition step, using pre-annotated entities in benchmarks artificially simplifies the problem. In real-world biomedical QA, entity recognition itself is a major challenge (e.g., ambiguous drug synonyms, rare disease names). Besides, the zero-shot extraction module is described but not evaluated. The paper should report precision/recall of entity recognition to show feasibility beyond curated inputs.

We thank the reviewer for raising this valuable point and to address this, we evaluated our zero-shot entity extraction module on the full set of benchmark questions. As reported in the new Supplementary Table S1, the model achieves high precision and recall (>0.90 across all entity types) and strong joint extraction accuracy (0.89–0.99). These results indicate that automated entity recognition is feasible for an end-to-end BTE-RAG workflow. We also note in the manuscript (Section 2.1, Entity Recognition) that integrating this module into a fully automated pipeline and evaluating it on unannotated biomedical queries is an important direction for future work.

3. No error analysis of BTE retrieval quality is provided. If BTE returns wrong or noisy triples, how often does this mislead the LLM? Adding experiment to show that would strengthen the study.

We thank the reviewer for highlighting the importance of evaluating retrieval quality and its downstream effects on model performance. We conducted a systematic error analysis of all cases in which BTE-RAG underperformed the LLM-only baseline, thereby directly quantifying how often noisy, incomplete, or misleading retrieved triples negatively influenced the LLM. This analysis revealed that most degradation cases were attributable to context dilution (noisy or overly broad retrieved evidence) or evidence coverage gaps (missing mechanistic edges), with only some contributions from filtering errors or derivative mismatches. These results are now presented in the revised Results section (Supplementary Table S6), with full case-level breakdowns provided in our repository at https://github.com/janjoy/BTE-RAG/tree/main/error_analysis.

4. Though the authors used SOTA LLMs, however, the choice of only OpenAI GPT-4o family is narrow. No comparison with open-source biomedical LLMs (e.g., BioGPT, Meditron, PubMedBERT-RAG). Comparison with these model would increase the generalizability

We appreciate the reviewer’s suggestion to broaden model comparisons and agree that diversity is important for generalizability. Our model selection was guided by two goals: (i) benchmarking BTE-RAG against strong baselines for biomedical question answering, and (ii) ensuring fair comparison with systems explicitly designed for mechanistic reasoning and structured retrieval.
In addition to GPT-4o and GPT-4o-mini, we therefore included GeneGPT (Full and Slim) RAG models, which is specifically designed for biomedical reasoning via structured retrieval from NCBI and BLAST resources. GeneGPT was evaluated alongside BTE-RAG on both an external benchmark (GeneTuring gene–disease association) and one of our mechanistic gene benchmarks, enabling assessment across independent datasets.
We did not include additional biomedical LLMs such as BioGPT or PubMedBERT models because prior large-scale evaluations show that these domain-specific models substantially underperform state-of-the-art general-purpose LLMs on complex biomedical reasoning tasks. For example, the GeneTuring benchmark

reports near-zero or very low accuracy for several biomedical LLMs, whereas ChatGPT-based models perform substantially better (Shang et al., 2025). Similarly, MultiMedQA evaluations demonstrate that larger general-purpose models such as GPT-4 outperform smaller domain-specific models by wide margins (Singhal et al., 2025).
Consistent with this literature, we selected GPT-4o family models as strong, widely used baselines and focused our evaluation on whether explicit mechanistic retrieval improves reasoning even from competitive starting points. We agree that extending BTE-RAG to additional open-source biomedical models is an important direction for future work.

5. Reliance on one source (DrugMechDB) makes evaluation narrow. The authors should demonstrate performance on at least one independent dataset (e.g., BioASQ, PubMedQA, SPOKE-based tasks) to show broader utility.

We thank the reviewer for this thoughtful comment, and we have now expanded our evaluation to include an independent benchmark, the GeneTuring gene–disease association dataset (which is distinct from DrugMechDB) and is used to assess large language models on genomics knowledge. On this benchmark, we compared LLM-only prompting, BTE-RAG, and two additional biomedical RAG approaches (GeneGPT-Full and GeneGPT-Slim). These results are now reported in Section 3.1 and Figure 2. Together, the inclusion of the GeneTuring benchmark and the DrugMechDB-derived mechanistic tasks demonstrates that BTE-RAG is effective both on an independent association-based dataset and on more complex and mechanistic reasoning problems.

6. Cosine similarity ≥0.9 is arbitrary; should provide ROC/AUC or threshold sensitivity.

We thank the reviewer for this suggestion. To address the concern about threshold selection, we have performed comprehensive threshold sensitivity analyses across 0.85, 0.90, and 0.95 cutoffs for both the metabolite and drug-to-biological-process benchmarks. These analyses, now included in the Results section (sections 3.3 and 3.4), demonstrate that BTE-RAG's relative improvements over LLM-only baselines remain consistent across all examined thresholds. For the metabolite task, we observed robust gains with effect sizes (Cliff's δ) ranging from 0.109 to 0.144 across thresholds. For the drug-to-biological-process task, minimal improvements (Cliff's δ: 0.000–0.018) were consistent across all cutoffs. We also report Wilcoxon signed-rank tests on per-item similarity differences to establish statistical significance.

7. Benchmarks enforce exactly one correct gene, metabolite, or drug per question. Real mechanisms often involve multiple parallel or interacting entities. The single-answer design hides biological complexity and creates an artificial task.

We appreciate this thoughtful comment and fully agree that biological mechanisms often involve multiple parallel or interacting entities. Our choice to use single-answer benchmarks from DrugMechDB was motivated by the need for objective, automated evaluation with clear ground truth that enables rigorous head-to-head comparison between LLM-only and BTE-RAG conditions. While this design necessarily simplifies biological complexity, it provides a reproducible and interpretable baseline for assessing whether knowledge graph context enhances mechanistic reasoning. Importantly, the BTE-RAG framework itself is not limited to single-entity outputs, and the retrieval step already surfaces multiple mechanistic candidates. We have added discussion of this limitation in the Discussion section, noting that real biomedical questions often have multiple valid answers involving pathway and network-based interactions. Future work will extend evaluation to multi-answer or network-based formats that better capture the parallelism and complexity of real biological systems.

8. Ground truth relies on exact HGNC, CHEBI, or DrugBank IDs. Why the ambiguities (synonyms, deprecated IDs, overlapping terms) are filtered out rather than addressed? This may bias the dataset toward easier, cleaner cases.

We appreciate this concern and would like to clarify our design. For the gene benchmark, we used HGNC symbols rather than IDs, as symbols are generally more natural for LLMs and reduce confusion from numerical identifiers. For metabolites and drugs, we did not rely on CHEBI or DrugBank IDs; instead, we evaluated model outputs against names using cosine similarity of BioBERT-STSB text embeddings, a metric

that captures graded lexical and semantic overlap rather than strict string matching. This approach inherently accommodates synonyms and overlapping terms while avoiding penalization for minor wording differences. Our primary goal in this work was to establish a fair comparison between LLM-only and BTE-RAG, and therefore we prioritized clarity and consistency in evaluation. In future iterations, we plan to extend the framework with explicit synonym resolution and deprecated mappings to further test robustness under noisier, real-world conditions.

9. The paper cited recent biomedical RAG systems such as BiomedRAG, GeneTuring but didn't compare with them (e.g., BiomedRAG). BioRAG (2024) is also highly relevant. These works are highly relevant baselines, showing retrieval from knowledge graphs, APIs, or literature, and including them in comparison would better position BTE-RAG within the current state of the art and highlight its unique contributions.

Thank you for this suggestion, we have now expanded our empirical evaluation to include GeneGPT, a representative biomedical RAG framework that integrates structured knowledge via NCBI (National Center for Biotechnology Information) APIs and in-context learning (Jin et al., 2024). Specifically, we evaluated both the GeneGPT-Full and GeneGPT-Slim configurations alongside LLM-only prompting and BTE-RAG on the GeneTuring gene–disease association benchmark as well as on our newly introduced mechanistic gene benchmark. These results are now reported in Sections 3.1 and 3.2, with detailed statistical analyses provided in the main text and supplementary materials. We agree that broader multi-model comparisons are valuable, and incorporating additional biomedical RAG systems and related approaches is an important direction for future work.

Editorial Manager®

Cookie Preference Center

We use cookies which are necessary to make our site work. We may also use additional cookies to analyze, improve and personalize our content and your digital experience. For more information, see our Cookie Policy.

You may choose not to allow some types of cookies. However, blocking some types may impact your experience of our site and the services we are able to offer. See the different category headings below to find out more or change your settings.

Allow all

Manage Consent Preferences

Strictly Necessary Cookies

Always active

These cookies are necessary for the website to function and cannot be switched off in our systems. They are usually only set in response to actions made by you which amount to a request for services, such as setting your privacy preferences, logging in or filling in forms. You can set your browser to block or alert you about these cookies, but some parts of the site will not then work.

Functional Cookies

These cookies enable the website to provide enhanced functionality and personalisation. They may be set by us or by third party providers whose services we have added to our pages. If you do not allow these cookies then some or all of these services may not function properly.

Performance Cookies

These cookies allow us to count visits and traffic sources so we can measure and improve the performance of our site. They help us to know which pages are the most and least popular and see how visitors move around the site.

Targeting Cookies

These cookies may be set through our site by our advertising partners. They may be used by those companies to build a profile of your interests and show you relevant adverts on other sites. If you do not allow these cookies, you will experience less targeted advertising.

Cookie List

Clear

Apply Cancel
Consent Leg.Interest

Confirm my choices

Powered by onetrust
