# Supplementary material for: Federated knowledge retrieval elevates large language model performance on biomedical benchmarks
Source: Gigascience. 2026 Jan 19;15:giag007. doi: 10.1093/gigascience/giag007 (PMC12888809; doi:10.1093/gigascience/giag007)
Supplement: giag007_Reviewer_1_Report_Original_Submission — Christopher Tabone, Ph.D. -- 9/3/2025 [file giag007_reviewer_1_report_original_submission.pdf]

Reviewer Report

Title: Federated Knowledge Retrieval Elevates Large Language Model Performance on Biomedical Benchmarks

Version: Original Submission **Date:** 9/3/2025

Reviewer name: Christopher Tabone, Ph.D.

Reviewer Comments to Author:

Dear Authors,
Thank you for the opportunity to review "Federated Knowledge Retrieval Elevates Large Language Model Performance on Biomedical Benchmarks." The paper tackles a timely and important problem: grounding large language models in mechanistic evidence to reduce unsupported claims. It does so with a thoughtful design that layers BTE-RAG over a federation of approximately 60 biomedical APIs and evaluates three complementary DrugMechDB-derived benchmarks (gene, metabolite, drug to process). The manuscript is clearly written, the technical contribution is meaningful, and the experimental results are promising.
Recommendation: Major revision.
Below are concrete, actionable changes that would bring the work in line with GigaScience's standards for FAIR availability, licensing, documentation, testing, and reproducibility. Many are straightforward, but together they matter for long-term reuse and auditability.
1) Statistical rigor: paired inference, uncertainty, variance
The manuscript reports compelling descriptive gains. Because each benchmark item is answered under both conditions (LLM-only and BTE-RAG), the study is a paired design. In paired settings, descriptive plots and point estimates are not sufficient to establish that improvements exceed sampling noise or threshold tuning. Please add paired statistical evidence that quantifies: (i) whether the gains are reliable, (ii) how large they are in practical terms, and (iii) how stable they are under repeated runs or under a fully deterministic pipeline.
Gene task (binary): Report McNemar's test on the existing 2Ã—2 tables, along with 95 percent Wilson confidence intervals for each condition and a Newcombe confidence interval for the accuracy difference. Keep the flip counts in the text.
Metabolite and drug-to-process tasks (similarity): Report paired bootstrap confidence intervals or Wilcoxon signed-rank tests on per-item similarity differences (BTE-RAG minus baseline). Include a nonparametric effect size such as Cliff's delta with its confidence interval.
Threshold validation: Treat the greater-than-or-equal-to 0.90 "high-fidelity" threshold as a choice that should be validated. Show sensitivity across nearby cutoffs such as 0.85, 0.90, and 0.95, and add a small blinded expert adjudication (about 50 to 100 items) to confirm that the high-cosine band corresponds to acceptable correctness.
Variance or determinism: Either document end-to-end determinism (frozen retrieval caches, fixed ordering, pinned embeddings) or run at least three replicates and report mean and standard deviation. These additions convert the current descriptive story into paired inference with uncertainty and effect

sizes and clarify robustness around thresholding and reproducibility.
2) Benchmark scope and generalizability
All three evaluations are derived from DrugMechDB, which makes the study internally consistent but also couples the tasks to a single curation philosophy and evidence distribution. Please acknowledge this limitation explicitly in the Discussion and, ideally, add an external validation on at least one independent source to demonstrate generalizability. Options include CTD (drug-gene-process links), Reactome or GO (pathway and process grounding), DisGeNET (gene-disease associations), or a lightweight question answering set sourced outside DrugMechDB. Even a modest external set of about 100 to 200 items, evaluated with the same paired protocols and identifier-based scoring, would strengthen the claim. If full external validation is not feasible for this revision, please include robustness checks such as a date-based split, entity-family holdouts, and per-source ablations.
3) Licensing, attribution, and persistent identifiers
The project is MIT-licensed and adapts components from BaranziniLab/KG_RAG (Apache-2.0) and SuLab/DrugMechDB (CC0-1.0). To meet license obligations and align with FAIR and the Joint Declaration of Data Citation Principles, please:
(i) keep Apache-licensed code under Apache with the upstream LICENSE and NOTICE files, noting any modifications;
(ii) include the CC0 dedication text for any DrugMechDB artifacts and note that CC0 provides no patent grant;
(iii) archive with DOIs (GigaDB preferred?) the three benchmarks, the exact evaluation caches used in the paper, and a tagged software release of the repository;
(iv) license datasets under CC0 or CC BY while keeping the code MIT;
(v) add a short Data and Software Availability table listing artifact, DOI or URL, license, and version or date.
4) Error analysis and degradation cases
Please add a brief failure analysis focused on where BTE-RAG reduces accuracy relative to LLM-only. At minimum, report the total number and percent of right-to-wrong flips per task and include a small set of representative cases. For each example, show the input, expected and predicted outputs, the top retrieved evidence with identifiers and timestamps, and a one-line diagnosis of the likely cause (for example normalization mismatch, retrieval coverage gap, ranking or filtering that hid relevant context, or long-context truncation). A short summary that groups the main causes into two or three buckets will make the results more interpretable and point to practical fixes.
5) Methodological transparency: embedding and scoring models
Please add two or three sentences in Methods explaining why S-PubMedBERT-MS-MARCO is used for filtering retrieved context while a BioBERT-based model is used for semantic similarity scoring, and what advantages each provides over plausible alternatives. A brief rationale will strengthen methodological transparency.
6) Reproducibility workflow and archived caches
Because BTE federates live APIs, results can drift as sources update. Please archive the exact retrieval caches used in evaluation with DOIs and minimal provenance if at all possible (query identifier, subject and object identifiers, predicate, source name and version or access date, any confidence score, and a retrieval timestamp).

In summary, this is a promising and well-motivated study that could make a useful contribution once the statistical evidence, FAIR availability, and reproducibility pieces are tightened as outlined above. I recommend Major Revision and am happy to re-review a revised version.

Methods

Are the methods appropriate to the aims of the study, are they well described, and are necessary controls included? Choose an item.

Conclusions

Are the conclusions adequately supported by the data shown? Choose an item.

Reporting Standards

Does the manuscript adhere to the journal's guidelines on minimum standards of reporting? Choose an item.

Choose an item.

Statistics

Are you able to assess all statistics in the manuscript, including the appropriateness of statistical tests used? Choose an item.

Quality of Written English

Please indicate the quality of language in the manuscript: Choose an item.

Declaration of Competing Interests

Please complete a declaration of competing interests, considering the following questions:

- Have you in the past five years received reimbursements, fees, funding, or salary from an organisation that may in any way gain or lose financially from the publication of this manuscript, either now or in the future?
- Do you hold any stocks or shares in an organisation that may in any way gain or lose financially from the publication of this manuscript, either now or in the future?
- Do you hold or are you currently applying for any patents relating to the content of the manuscript?
- Have you received reimbursements, fees, funding, or salary from an organization that holds or has applied for patents relating to the content of the manuscript?
- Do you have any other financial competing interests?
- Do you have any non-financial competing interests in relation to this paper?

If you can answer no to all of the above, write 'I declare that I have no competing interests' below. If your reply is yes to any, please give details below.

I declare that I have no competing interests.

I agree to the open peer review policy of the journal. I understand that my name will be included on my report to the authors and, if the manuscript is accepted for publication, my named report including any attachments I upload will be posted on the website along with the authors' responses. I agree for my report to be made available under an Open Access Creative Commons CC-BY license (http://creativecommons.org/licenses/by/4.0/). I understand that any comments which I do not wish to be included in my named report can be included as confidential comments to the editors, which will not be published.

Choose an item.

To further support our reviewers, we have joined with Publons, where you can gain additional credit to further highlight your hard work (see: https://publons.com/journal/530/gigascience). On publication of this paper, your review will be automatically added to Publons, you can then choose whether or not to claim your Publons credit. I understand this statement.

Yes Choose an item.
