# Supplementary material for: Federated knowledge retrieval elevates large language model performance on biomedical benchmarks
Source: Gigascience. 2026 Jan 19;15:giag007. doi: 10.1093/gigascience/giag007 (PMC12888809; doi:10.1093/gigascience/giag007)
Supplement: giag007_Reviewer_1_Report_Revision_1 — Christopher Tabone, Ph.D. -- 12/27/2025 [file giag007_reviewer_1_report_revision_1.pdf]

Reviewer Report

Title: Federated Knowledge Retrieval Elevates Large Language Model Performance on Biomedical Benchmarks

Version: Revision 1 **Date:** 12/27/2025

Reviewer name: Christopher Tabone, Ph.D.

Reviewer Comments to Author:

Dear Authors,

Thank you for your thorough and thoughtful revision of this manuscript. The scope of work undertaken to address the reviewers' concerns is substantial and reflects a genuine commitment to scientific rigor and reproducibility. You have implemented comprehensive statistical analyses across all benchmarks, integrated an entirely new external validation dataset, conducted systematic error analyses, restructured your licensing framework, and made extensive reproducibility materials publicly available. You accomplished all of this while maintaining clarity and coherence throughout the manuscript. This level of responsiveness to peer review is exemplary.

Below I summarize my assessment of the key revisions.

Comparative Baseline Expansion

The integration of GeneGPT (both Full and Slim configurations) as baseline comparators meaningfully positions BTE-RAG within the existing landscape of biomedical retrieval-augmented generation systems. The results presented in Sections 3.1 and 3.2 clearly demonstrate consistent improvements across model scales. Your literature-based justification for the model selection is well-reasoned and scientifically sound.

Statistical Rigor

The statistical framework is rigorous and appropriate. The application of McNemar's tests with Wilson and Newcombe confidence intervals for binary tasks, and Wilcoxon signed-rank tests with Cliff's delta for similarity-based tasks, represents current best practices for paired study designs. The contingency table analyses (e.g., 245 corrections versus 47 reversals for GPT-4o-mini) provide compelling evidence that the observed improvements are systematic. I particularly appreciate the transparent reporting of negligible effect sizes for the drug-biological process task, which strengthens the manuscript's credibility.

The threshold sensitivity analyses across 0.85, 0.90, and 0.95 cutoffs adequately address concerns about arbitrary threshold selection and demonstrate robust findings.

Technical and FAIR Compliance

The transition to Apache 2.0 licensing for source code and CC0 1.0 dedication for benchmarks satisfies licensing obligations and aligns with FAIR principles. The comprehensive archival of BTE retrieval caches, LLM outputs, and evaluation metrics substantially strengthens reproducibility. Please ensure that DOI-backed deposition through Software Heritage or an equivalent archive is completed prior to publication.

Error Analysis

The failure analysis in Supplementary Table S6 is a valuable addition. The categorization into context

dilution, evidence coverage gaps, filtering errors, and derivative mismatches provides actionable diagnostic information that will guide future improvements in retrieval-augmented biomedical reasoning systems.

Entity Recognition Evaluation

The zero-shot entity recognition evaluation in Supplementary Table S1 provides sufficient evidence that automated extraction is technically feasible for end-to-end deployment. Your acknowledgment that validation on unannotated real-world queries remains future work is appropriately measured.

Technical Observations

The dual-query TRAPI architecture and the principled separation of retrieval and evaluation models (Section 2.1) are methodologically sound. Including a representative TRAPI query structure in the Supplementary Materials could further facilitate replication by other research groups, though the existing code repository provides sufficient detail.

Optional Suggestion

At your discretion, a brief clarification in Section 2.2.1 noting that the GeneGPT comparison uses an adapted implementation (GPT-4o backend) rather than the original Codex-based system would add precision. This is not required but would preempt potential reader questions about direct comparability to the published GeneGPT results.

Summary

The manuscript now presents a robust statistical framework, meaningful baseline comparisons, transparent error analysis, and comprehensive archival of all materials necessary for reproduction. The contribution to retrieval-augmented biomedical reasoning is both methodologically sound and practically valuable, and will be of interest to researchers working at the intersection of knowledge graphs and large language models.

Thank you for your dedication to producing high-quality, reproducible science.

Methods

Are the methods appropriate to the aims of the study, are they well described, and are necessary controls included? Choose an item.

Conclusions

Are the conclusions adequately supported by the data shown? Choose an item.

Reporting Standards

Does the manuscript adhere to the journal’s guidelines on [minimum standards of reporting?]() Choose an item.

Choose an item.

Statistics

Are you able to assess all statistics in the manuscript, including the appropriateness of statistical tests used? Choose an item.

Quality of Written English

Please indicate the quality of language in the manuscript: Choose an item.

Declaration of Competing Interests

Please complete a declaration of competing interests, considering the following questions:

- Have you in the past five years received reimbursements, fees, funding, or salary from an organisation that may in any way gain or lose financially from the publication of this manuscript, either now or in the future?
- Do you hold any stocks or shares in an organisation that may in any way gain or lose financially from the publication of this manuscript, either now or in the future?
- Do you hold or are you currently applying for any patents relating to the content of the manuscript?
- Have you received reimbursements, fees, funding, or salary from an organization that holds or has applied for patents relating to the content of the manuscript?
- Do you have any other financial competing interests?
- Do you have any non-financial competing interests in relation to this paper?

If you can answer no to all of the above, write 'I declare that I have no competing interests' below. If your reply is yes to any, please give details below.

I declare that I have no competing interests.

I agree to the open peer review policy of the journal. I understand that my name will be included on my report to the authors and, if the manuscript is accepted for publication, my named report including any attachments I upload will be posted on the website along with the authors' responses. I agree for my report to be made available under an Open Access Creative Commons CC-BY license (http://creativecommons.org/licenses/by/4.0/). I understand that any comments which I do not wish to be included in my named report can be included as confidential comments to the editors, which will not be published.

Choose an item.

To further support our reviewers, we have joined with Publons, where you can gain additional credit to further highlight your hard work (see: https://publons.com/journal/530/gigascience). On publication of this paper, your review will be automatically added to Publons, you can then choose whether or not to claim your Publons credit. I understand this statement.

Yes Choose an item.
