# Supplementary material for: Federated knowledge retrieval elevates large language model performance on biomedical benchmarks
Source: Gigascience. 2026 Jan 19;15:giag007. doi: 10.1093/gigascience/giag007 (PMC12888809; doi:10.1093/gigascience/giag007)
Supplement: giag007_Reviewer_2_Report_Original_Submission — Sajib Acharjee Dip -- 9/4/2025 [file giag007_reviewer_2_report_original_submission.pdf]

Reviewer Report

Title: Federated Knowledge Retrieval Elevates Large Language Model Performance on Biomedical Benchmarks

Version: Original Submission **Date:** 9/4/2025

Reviewer name: Sajib Acharjee Dip

Reviewer Comments to Author:

This paper introduces BTE-RAG, a system that combines large language models with biomedical knowledge from BioThings Explorer. Tested on three benchmarks built from DrugMechDB (genes, metabolites, and drug-process links), it shows clear accuracy gains compared to using LLMs alone.
Strengths:
The work demonstrates that retrieval improves both small and large models, suggesting cost-efficiency and scalability. This paper also curated multi-scale QA datasets (gene, metabolite, drug) from DrugMechDB provide structured, reproducible evaluation.
Weaknesses:
1. This dual-route design is conceptually sound but too narrow a baseline. A stronger evaluation would compare against other RAG systems (PubMed-based retrieval, BiomedRAG, SPOKE-RAG) instead of just "LLM-only."
2. For Entity Recognition step, using pre-annotated entities in benchmarks artificially simplifies the problem. In real-world biomedical QA, entity recognition itself is a major challenge (e.g., ambiguous drug synonyms, rare disease names). Besides, the zero-shot extraction module is described but not evaluated. The paper should report precision/recall of entity recognition to show feasibility beyond curated inputs.
3. No error analysis of BTE retrieval quality is provided. If BTE returns wrong or noisy triples, how often does this mislead the LLM? Adding experiment to show that would strengthen the study.
4. Though the authors used SOTA LLMs, however, the choice of only OpenAI GPT-4o family is narrow. No comparison with open-source biomedical LLMs (e.g., BioGPT, Meditron, PubMedBERT-RAG). Comparison with these model would increase the generalizability
5. Reliance on one source (DrugMechDB) makes evaluation narrow. The authors should demonstrate performance on at least one independent dataset (e.g., BioASQ, PubMedQA, SPOKE-based tasks) to show broader utility.
6. Cosine similarity â‰¥0.9 is arbitrary; should provide ROC/AUC or threshold sensitivity.
7. Benchmarks enforce exactly one correct gene, metabolite, or drug per question. Real mechanisms often involve multiple parallel or interacting entities. The single-answer design hides biological complexity and creates an artificial task.
8. Ground truth relies on exact HGNC, CHEBI, or DrugBank IDs. Why the ambiguities (synonyms, deprecated IDs, overlapping terms) are filtered out rather than addressed? This may bias the dataset toward easier, cleaner cases.
9. The paper cited recent biomedical RAG systems such as BiomedRAG, GeneTuring but didn't compare

with them (e.g., BiomedRAG). BioRAG (2024) is also highly relevant. These works are highly relevant baselines, showing retrieval from knowledge graphs, APIs, or literature, and including them in comparison would better position BTE-RAG within the current state of the art and highlight its unique contributions.

Methods

Are the methods appropriate to the aims of the study, are they well described, and are necessary controls included? Choose an item.

Conclusions

Are the conclusions adequately supported by the data shown? Choose an item.

Reporting Standards

Does the manuscript adhere to the journal's guidelines on [minimum standards of reporting?] Choose an item.

Choose an item.

Statistics

Are you able to assess all statistics in the manuscript, including the appropriateness of statistical tests used? Choose an item.

Quality of Written English

Please indicate the quality of language in the manuscript: Choose an item.

Declaration of Competing Interests

Please complete a declaration of competing interests, considering the following questions:

- Have you in the past five years received reimbursements, fees, funding, or salary from an organisation that may in any way gain or lose financially from the publication of this manuscript, either now or in the future?
- Do you hold any stocks or shares in an organisation that may in any way gain or lose financially from the publication of this manuscript, either now or in the future?
- Do you hold or are you currently applying for any patents relating to the content of the manuscript?
- Have you received reimbursements, fees, funding, or salary from an organization that holds or has applied for patents relating to the content of the manuscript?
- Do you have any other financial competing interests?
- Do you have any non-financial competing interests in relation to this paper?

If you can answer no to all of the above, write 'I declare that I have no competing interests' below. If your reply is yes to any, please give details below.

I declare that I have no competing interests.

I agree to the open peer review policy of the journal. I understand that my name will be included on my report to the authors and, if the manuscript is accepted for publication, my named report including any attachments I upload will be posted on the website along with the authors' responses. I agree for my report to be made available under an Open Access Creative Commons CC-BY license (http://creativecommons.org/licenses/by/4.0/). I understand that any comments which I do not wish to be included in my named report can be included as confidential comments to the editors, which will not be published.

Choose an item.

To further support our reviewers, we have joined with Publons, where you can gain additional credit to further highlight your hard work (see: https://publons.com/journal/530/gigascience). On publication of this paper, your review will be automatically added to Publons, you can then choose whether or not to claim your Publons credit. I understand this statement.

Yes Choose an item.
