# Supplementary material for: Federated knowledge retrieval elevates large language model performance on biomedical benchmarks
Source: Gigascience. 2026 Jan 19;15:giag007. doi: 10.1093/gigascience/giag007 (PMC12888809; doi:10.1093/gigascience/giag007)
Supplement: giag007_Reviewer_2_Report_Revision_1 — Sajib Acharjee Dip -- 1/2/2026 [file giag007_reviewer_2_report_revision_1.pdf]

Reviewer Report

Title: Federated Knowledge Retrieval Elevates Large Language Model Performance on Biomedical Benchmarks

Version: Revision 1 **Date:** 1/2/2026

Reviewer name: Sajib Acharjee Dip

Reviewer Comments to Author:

The authors have carefully and substantively addressed the major concerns raised in the initial review. In particular, the added error analysis of BTE retrieval failures, the evaluation of the zero-shot entity extraction module, the inclusion of paired statistical testing, and the expansion to an independent benchmark (GeneTuring) significantly strengthen the rigor and credibility of the study. These additions clarify when and why retrieval helps, when it can hurt, and demonstrate that the reported gains are robust rather than anecdotal.

While the evaluation could be further strengthened in the future by comparisons to additional biomedical RAG systems (e.g., literature-based or KG-only approaches) or by testing on open-source biomedical language models, these are now clearly framed as scope limitations rather than methodological gaps. The current experimental design is coherent, well-justified, and sufficient to support the paper's core claims.

Overall, this work provides a clear and useful contribution to the study of retrieval-augmented generation for biomedical reasoning, with strong emphasis on mechanistic grounding, reproducibility, and transparency. I believe it meets the standards for publication, and the remaining concerns are minor and appropriately acknowledged by the authors.

Methods

Are the methods appropriate to the aims of the study, are they well described, and are necessary controls included? Choose an item.

Conclusions

Are the conclusions adequately supported by the data shown? Choose an item.

Reporting Standards

Does the manuscript adhere to the journal’s guidelines on minimum standards of reporting? Choose an item.

Choose an item.

Statistics

Are you able to assess all statistics in the manuscript, including the appropriateness of statistical tests used? Choose an item.

Quality of Written English

Please indicate the quality of language in the manuscript: Choose an item.

Declaration of Competing Interests

Please complete a declaration of competing interests, considering the following questions:

- Have you in the past five years received reimbursements, fees, funding, or salary from an organisation that may in any way gain or lose financially from the publication of this manuscript, either now or in the future?
- Do you hold any stocks or shares in an organisation that may in any way gain or lose financially from the publication of this manuscript, either now or in the future?
- Do you hold or are you currently applying for any patents relating to the content of the manuscript?
- Have you received reimbursements, fees, funding, or salary from an organization that holds or has applied for patents relating to the content of the manuscript?
- Do you have any other financial competing interests?
- Do you have any non-financial competing interests in relation to this paper?

If you can answer no to all of the above, write 'I declare that I have no competing interests' below. If your reply is yes to any, please give details below.

I declare that I have no competing interests.

I agree to the open peer review policy of the journal. I understand that my name will be included on my report to the authors and, if the manuscript is accepted for publication, my named report including any attachments I upload will be posted on the website along with the authors' responses. I agree for my report to be made available under an Open Access Creative Commons CC-BY license (http://creativecommons.org/licenses/by/4.0/). I understand that any comments which I do not wish to be included in my named report can be included as confidential comments to the editors, which will not be published.

Choose an item.

To further support our reviewers, we have joined with Publons, where you can gain additional credit to further highlight your hard work (see: https://publons.com/journal/530/gigascience). On publication of this paper, your review will be automatically added to Publons, you can then choose whether or not to claim your Publons credit. I understand this statement.

Yes Choose an item.
